# Zooplankton Growth, Respiration and Grazing on the Australian Margins of the Tropical Indian and Pacific Oceans

**DOI:** 10.1371/journal.pone.0140012

**Published:** 2015-10-15

**Authors:** A. David McKinnon, Jason Doyle, Samantha Duggan, Murray Logan, Christian Lønborg, Richard Brinkman

**Affiliations:** 1 Australian Institute of Marine Science, P.M.B. No. 3, Townsville M.C. 4810, Queensland, Australia; 2 The Western Australian Marine Science Institution, Perth, Western Australia, Australia; 3 School of Marine and Tropical Biology, James Cook University, Townsville 4810, Queensland, Australia; University of Shiga Prefecture, JAPAN

## Abstract

The specific activity of aminoacyl-tRNA synthetases (spAARS), an index of growth rate, and of the electron transport system (spETS), an index of respiration, was measured in three size fractions (73–150 μm, >150 μm and >350 μm) of zooplankton during five cruises to tropical coastal waters of the Kimberley coast (North West Australia) and four cruises to waters of the Great Barrier Reef (GBR; North East Australia). The N-specific biomass of plankton was 3–4-fold higher in the Kimberley than on the GBR in all 3 size classes: Kimberley 1.27, 3.63, 1.94 mg m^-3^; GBR 0.36, 0.88 and 0.58 mg m^-3^ in the 73–150 μm, >150 μm and >350 μm size classes, respectively. Similarly, spAARS activity in the Kimberley was greater than that of the GBR: 88.4, 132.2, and 147.6 nmol PPi hr^-1^ mg protein ^-1^ in the Kimberley compared with 71.7, 82.0 and 83.8 nmol PPi hr^-1^ mg protein ^-1^ in the GBR, for the 73–150 μm, >150 μm and >350 μm size classes, respectively. Specific ETS activity showed similar differences in scale between the two coasts: 184.6, 148.8 and 92.2 μL O_2_ hr^-1^ mg protein^-1^ in the Kimberley, against 86.5, 88.3 and 71.3 μL O_2_ hr^-1^ mg protein^-1^ in the GBR. On the basis of these measurements, we calculated that >150 μm zooplankton grazing accounted for 7% of primary production in the Kimberley and 8% in GBR waters. Area-specific respiration by >73 μm zooplankton was 7-fold higher in the Kimberley than on the GBR and production by >150 μm zooplankton was of the order of 278 mg C m^-2^ d^-1^ in the Kimberley and 42 mg C m^-2^ d^-1^ on the GBR. We hypothesize that the much stronger physical forcing on the North West shelf is the principal driver of higher rates in the west than in the east of the continent.

## Introduction

The continent of Australia separates the Indian and Pacific Oceans by up to 40 degrees of longitude, and the tropical seas of Australia extend over 13 degrees of latitude. The highest primary productivity per unit chlorophyll *a* occurs on the North West (NW) shelf of Australia (Indian Ocean), but Pacific Ocean waters on the North East (NE) shelf adjacent to the Great Barrier Reef (GBR) are comparatively oligotrophic [1]. Though primary production in northern Australian waters can be episodically high [1,2], fish production is uniformly low. For example, in NW Australia, low fish production (~4,000 tonnes p.a. in the North Coast bioregion) has been attributed to low values of primary and secondary production [3]. Fisheries production on the GBR is also low–finfish production is <6,000 tonnes p.a. [4]. The relationship between primary production and fisheries production in Australian waters is poorly understood, and highlights the lack of empirical data on the fate of primary production, particularly the role of zooplankton as intermediaries between primary producers and higher trophic levels. Generally, fishery production in tropical marginal seas correlates with primary production estimated from remotely sensed chlorophyll *a* concentration [5], but primary production was found to be a poor predictor of fishery yields in 52 large marine ecosystems, and chlorophyll *a* concentration, particle-export ratio, and the ratio of secondary to primary production were better predictors [6]. By necessity, biogeochemical models in Australia have applied theoretical grazing rates to primary production in order to balance fluxes with observed standing stocks (usually expressed in terms of chlorophyll *a*). There are, however, few measurements of zooplankton production in Australian waters to better parameterize the relationship between primary production and fish production and even fewer measurements of grazing rates to constrain biogeochemical models.

Globally, ~40% of the primary production of coastal phytoplankton is consumed by herbivores, ~40% is decomposed within the ecosystem, ~16% is exported (by horizontal transport) and only ~4% is stored within sediments [7]. Within the GBR ecosystem, pelagic microbial respiration rates (= decomposition in the parlance of [7]) accounts for ~63% of gross pelagic primary production [8], but sedimentation rates are not particularly high and there is negligible net burial of C and N in subtidal sediments [9]. Consequently, the balance of GBR production not respired (~40%) must be split between export and grazing. Export of pelagic production is dependent on physical oceanographic processes. Within the GBR “lagoon”—the body of open water between the reef matrix and the coastline—there is debate about water residence times, but estimates converge on a time scale of ~one month (see discussion in [8]). In the macrotidal environments of NW Australia this is likely to be less for open water areas but may be comparable for semi enclosed embayments. Grazing is likely to account for a large proportion of the 40% of primary production not respired, but there are presently very few data to better constrain this term.

There are few direct measurements of primary production in northern Australia (summarised in [8]). In the El Niňo summer of 1997–98, primary production at the shelf edge near Australia’s NW Cape averaged 3.1 g C m^-2^ d^-1^ (with a maximum of 8 g C m^-2^ d^-1^), but in the more typical La Niňa conditions of 1998–99, primary production averaged 1.3 g C m^-2^ d^-1^ [2]. The few comparable measurements available from the inshore waters of the Kimberley region of NW Australia suggest rates of 1.5–3.5 g C m^-2^ d^-1^, from a very shallow (<10 m) but highly productive euphotic zone (Furnas, pers. comm.). By contrast, primary production in the waters of the GBR is about 0.7 g C m^-2^ d^-1^ [8,10], though it can be considerably higher during episodes of freshwater input or upwelling of sub-thermocline waters from the Coral Sea. Since the phytoplankton communities of northern Australian waters are dominated by picoplankton [11,12], the most important grazers of pelagic primary producers are protists [13], though the contribution of pelagic tunicates is yet to be quantified and may be substantial. Mesozooplankton are the critical link to fishes, especially their larvae (e.g. [14]). Copepods are the dominant mesozooplankton in tropical Australian waters [15] and are important predators of microzooplankton [16]. Consequently, the transfer of carbon from primary producers to upper trophic levels via mesozooplankton involves many microbial linkages at the lower end of the food web [17], all of which are poorly documented in Australian waters (but see [18–20]). Mesozooplankton grazing, likewise, has only been directly measured a few times (e.g. [21,22]). One of the problems facing grazing experiments in tropical waters is that the standing stock of phytoplankton is usually very low, meaning that the main trophic resources available to mesozooplankton are either protistan grazers or detrital flocs or aggregates. Unfortunately, these are hard to quantify in experimental manipulations. Alternative approaches to estimating grazing rates, for example calculating ingestion rates on the basis of directly measured rates such as somatic growth [23,24] or copepod egg production [25] using widely applied conversion factors, have yielded rates that are so low that they do not adequately account for either the fate of primary production or for the metabolic needs of the zooplankton.

Copepod growth is usually measured using cohort development or egg production methods. To estimate grazing of copepods, a number of methods are available, such as bottle feeding experiments, gut fluorescence and radioisotope techniques [26]. Most of these methods require experimental manipulation of the animals and an incubation period during which the food environment experienced by the animals may differ from that *in situ*. Biochemical indices related to increases in structural growth and respiration of plankton are an attractive alternative to more widely applied methods specific to copepods and are based on *in situ* conditions prior to the time of collection, thereby eliminating both the necessity for incubations in the field and the problem of bottle effects [27]. Moreover, biochemical indices represent instantaneous rates in a period shorter than that estimated by traditional methods. In this contribution we measure specific activities of aminoacyl-tRNA synthetases (spAARS) and the electron transport system (spETS) in zooplankton collected from continental shelf waters of both NW and NE Australia (inshore waters of the Kimberley region and the waters of the GBR lagoon and adjacent Coral Sea). Our goal is to compare growth, respiration and production of tropical zooplankton from waters of NW and NE Australia. We then use these estimates of growth and respiration to calculate grazing rates to better understand the fate of primary production in Australian tropical waters and the nature of pelagic food chains in these regions.

## Material and Methods

### Study areas

**Fig 1:**
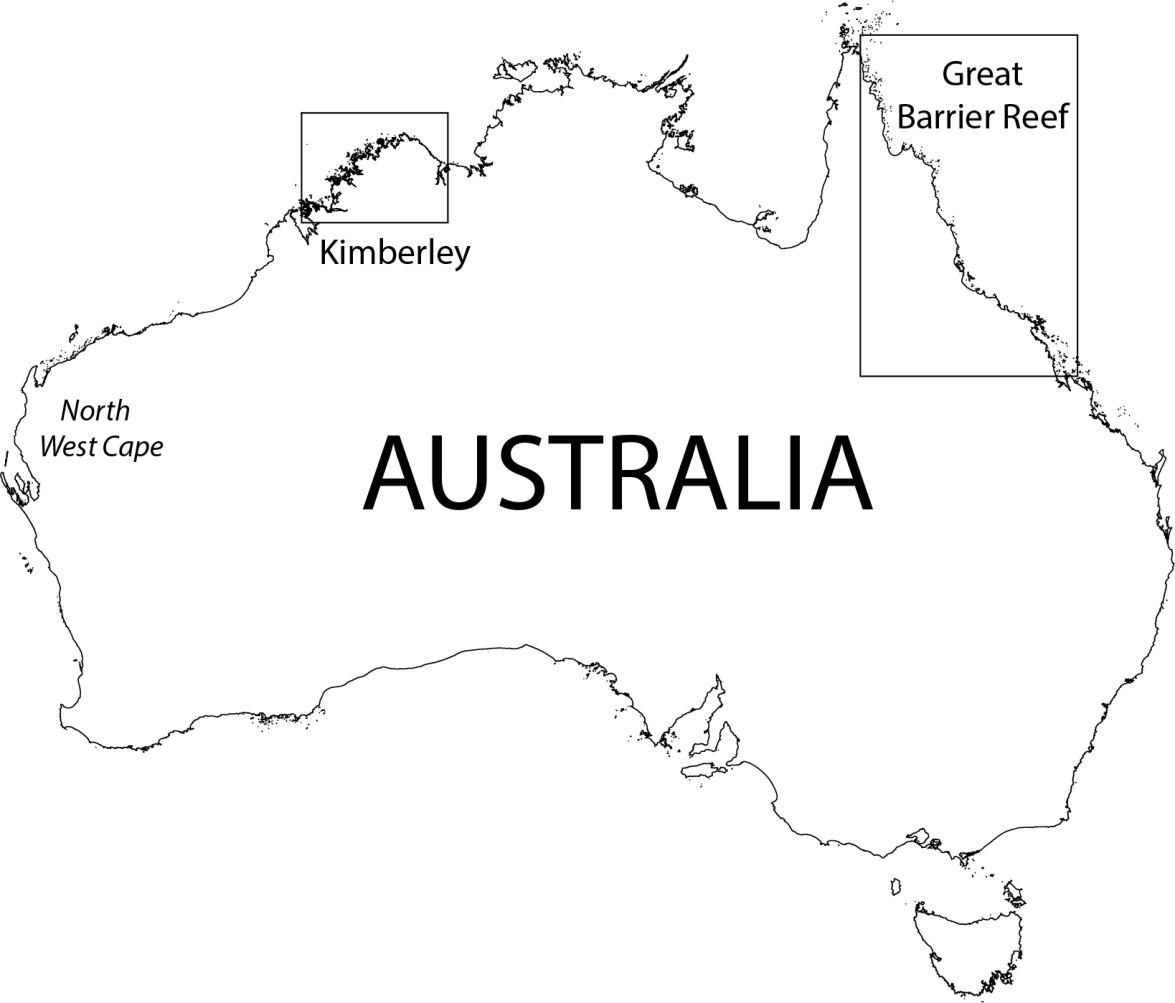
Map of Australia, showing the two different regions in this study–the Great Barrier Reef on the North East coast, and the Kimberley region on the North West coast.

Australia’s tropical seas comprise extensive continental shelf ecosystems extending into Indonesian seas in the NW, the Timor and Arafura Seas in the North, and the waters of the Great Barrier Reef (GBR), the world’s largest coral reef ecosystem, in the NE. In this study, plankton was sampled from the Kimberley coast (NW Australia) and from the GBR (Fig 1). The Kimberley coast is predominantly macrotidal (tides up to 11.7 m) but tidal range diminishes to <4 m at the eastern extent of our study area, whereas tides on the GBR vary according to latitude and shelf location, but for the most part have a range <4 m. Kimberley coastal waters are highly turbid and well-mixed, with a shallow euphotic zone, whereas on the GBR waters are predominantly very clear (except on the coast and the mangrove fringe) with the euphotic zone generally extending to the bottom. Currents on both shelves are influenced by monsoonal wind patterns. On the GBR, SE trade winds are prevalent between April and November, but these relax during the summer months (December to March) and winds are variable and generally weaker. In the central GBR the trade winds force northward flowing coastal currents, whereas further offshore the southward flowing East Australian Current results in a net southward flow [28]. On the NW shelf, flow is toward the NE during the summer, but the predominant currents reverse toward the SW after relaxation of the NW monsoon [29].

Our GBR samples were all taken during the dry season (April–November), but the Kimberley samples were collected on discrete summer wet and winter dry season cruises (Table 1). All collections in the GBR were covered by a permit from the Great Barrier Reef Marine Park Authority (G12/35236.1). No specific permissions were required for the collection of plankton from the Kimberley region.

**Table 1:** Stations occupied on each cruise.

Area	Stations	Dates
**Great Barrier Reef**	CSC046 –CSC050	16–19 Aug. 2012
	CSC051 –CSC070	17–23 Nov. 2012
	CSC072 –CSC080	25–28 Sep. 2013
	CSC081 –CSC098	6–18 Jun. 2014
**Kimberley coast**	KIM002 –KIM045	20–30 Jan. 2011
	KIM053 –KIM107	11–22 Oct. 2011
	KIM115 –KIM194	28 Feb–10 Mar. 2013
	KIM197 –KIM272	23–30 Oct. 2013
	KIM273 –KIM370	11–22 Mar. 2014

### Environmental data

CTD (Seabird SBE19 on east coast, SB911 on west coast) casts were made to near-bottom at each station. Water samples, collected at 3–5 depths through the water column for chlorophyll *a* concentration, were filtered onto GF/F filters, which were then frozen (-10°C) and stored until subsequent grinding and extraction in 90% acetone and analyzed by fluorometry [30]. Chlorophyll *a* values were used to calibrate data from a WetLabs Wetstar fluorometer fitted to the CTD.

### Zooplankton size-fractionation

Plankton samples were collected by oblique tows throughout the water column with a purpose-built bongo net that size-fractionates the zooplankton community *in situ* [24]. One side of the bongo net was fitted with a WP-2 net of 350 μm plankton mesh, and the other side with a 150 μm plankton net of 34 cm diameter nested inside a 73 μm net of 50 cm diameter. This net array allowed us to collect zooplankton samples fractionated into >350 μm, >150 μm and 73–150 μm size ranges. Hydrobios electronic flowmeters were mounted off-centre in the mouths of the 150 μm and 350 μm nets [31]. Each net sample was split into three portions: half was transferred into cryovials and frozen in liquid nitrogen for subsequent enzymatic assays, one quarter was preserved in formaldehyde for analysis of community composition (reported in [32] and McKinnon unpublished), and one quarter filtered onto a pre-weighed disk of 73 μm mesh and frozen. The frozen mesh was subsequently dried (65°C) and re-weighed to estimate zooplankton community biomass as dry weight. The dried plankton was then ground and analyzed for C and N content on a Shimadzu CN analyzer.

Complementary samples were collected at five stations adjacent to the outer margin of the GBR (CSC046, CSC074, CSC075 off Elusive Reef, CSC056 off Mantis Reef and CSC089 off Hicks Reef) to determine plankton vertical distribution using a Hydrobios multinet system with a mouth area of 0.25 m^2^ and a mesh size of 100 μm. Sampling depth intervals were set to be nominally 250–200 m, 200–150 m, 150–100 m, 100–50 m (containing the chlorophyll *a* maximum), and 50 m to the surface, though depths were modified on 2 occasions (CSC046 and CSC056). These samples were processed using the same protocols as for the bongo net samples.

### Aminoacyl-tRNA synthetases (AARS) assay

Aminoacyl-tRNA synthetases (AARS) catalyze the first step of protein synthesis. Their activity is correlated with somatic growth in freshwater and marine crustaceans, and can be used as an index of copepod somatic growth [33].

Frozen zooplankton samples from each size fraction were removed from -80°C storage and homogenized via sonication (2 x 1 min with cooling between sonication) on ice in 4–8 volumes of ice-cold Tris pH 7.8. An aliquot was taken for biomass determination and the remaining homogenate centrifuged at 3000 x g, 0°C for 15 min to remove cell debris. The clarified homogenate was serially diluted and dilutions assayed for spAARS activity following the method of Chang et al. [34] and Yebra and Hernández-León [33] with modifications for a 96-well plate format. In detail, 120 μl water and 80 μl pyrophosphate reagent (Product No. P7275, Sigma, St Louis, MO, USA) were added to the wells of a 96-well plate and incubated in a plate reader at 30°C for 5–10 min. The reaction was initiated by adding 100 μl of clarified homogenate dilutions with careful mixing using a multichannel pipette, ensuring no bubbles formed within the wells that would interfere with the absorbance readings. It was necessary to test various dilutions of the homogenate as this ensured at least two dilutions were within assay linearity. A blank measurement using 100 μl Tris pH 7.8 only was included in each assay plate. The incubation temperature was set to 30°C for kinetic readings with measurements at 340 nm made every 30 secs for a total of 35 min. The first 10 min of the kinetic read was ignored due to assay temperature equilibration. Protein concentration (mg ml^-1^) was determined using the Lowry [35] method (Biorad, CA, USA) and samples that fell below the detection limit of this technique were analyzed via the micro bicinchoninic acid method (Thermo, VIC, Australia). Specific AARS activity measured as the rate of pyrophosphate production (nmol PPi hr^-1^ mg protein^-1^) was determined according to the equation:

$$\text{spAARS}\;({\text{nmol PPi hr}}^{\text{-}1}\;{\text{mg protein}}^{\text{-}1})=(\text{slope}\times 60\times 0.3)/(6.22\times 0.82\times 2\times 0.1\times \text{P})$$ (1)

where slope is the rate of change (in milli absorbance units min^-1^) at 340 nm in undiluted homogenate calculated from at least 2 dilutions, 60 converts minutes to hours, 0.3 is the reaction mixture volume (ml), 6.22 is the millimolar absorbtivity of β-nicotinamide adenine dinucleotide reduced form (NADH) at 340 nm, 0.82 is a path-length correction factor, 2 is the moles NADH oxidised per mole PPi consumed, 0.1 is the homogenate volume (ml) and P is the protein concentration of the homogenate (mg ml^-1^). Specific AARS activity was corrected for *in situ* temperature using the Arrhenius equation [36] with an activation energy of 35.8 kJ mole^-1^ [37].

### Electron Transport System (ETS) assay

The electron transport system (ETS) is nearly ubiquitous in mitochondrial membranes, and can be used as an indicator of organic matter remineralisation, as it consists of a complex chain of cytochromes, flavo proteins and metabolic ions that transport electrons from catabolised food to oxygen. ETS activity is correlated to *in vivo* respiration (e.g. [38]), so that ETS activity can be used as an estimate of mesozooplankton respiration rate.

The ETS assay was conducted according to the methods of Kenner and Ahmed [39], and Owens and King [40] and adapted for a 96-well plate. Reagents used were: homogenization buffer (0.05 M phosphate buffer pH 8.0, 0.2% v/v triton x-100, 0.15% w/v polyvinylpyrrolidone, 75 μM MgSO4), substrate buffer (0.05M phosphate buffer pH 8.0, 0.2% v/v triton x-100), substrate solution (0.25mM β-nicotinamide adenine dinucleotide 2’phosphate, reduced form [NADPH], 0.835mM NADH and 133mM succinate dissolved in substrate buffer) and INT solution (2 mg/ml 2-(*p*-iodophenyl)-3-(*p*-nitrophenyl)-5-phenyltetrazolium chloride dissolved in water). Frozen zooplankton samples were removed from -80°C storage and homogenized via sonication (1 x 1 minute and 1 x 30 seconds with cooling between sonication) on ice in 50 volumes of ice-cold homogenization buffer. The homogenate was centrifuged at 3000 x g, 0°C for 15 minutes to remove cell debris and the clarified homogenate serially diluted prior to assay for ETS activity. Substrate solution (150 μl) and INT solution (50 μl) were added to the wells of a 96-well plate and incubated in a plate reader at 30xC for 5–10 minutes. The reaction was initiated by adding 50 μl of clarified homogenate dilutions with careful mixing using a multichannel pipette, ensuring no bubbles formed within the wells which would interfere with the absorbance readings. A blank measurement using 50 μl homogenization buffer only was included in each assay plate. Absorbances at 490 nm were measured as per the AARS assay and protein concentrations (mg.ml^-1^) were determined by the micro bicinchoninic acid method (Thermo, VIC, Australia). Specific ETS activity (spETS) measured as equivalent oxygen utilization (μl O_2_ hr^-1^ mg protein^-1^) was determined using the equation:

$$\text{spETS}\;(\mu \text{l O}2\;{\text{hr}}^{\text{-}1}\;{\text{mg protein}}^{\text{-}1})=(\text{slope}\times 60\times 0.25)/(1.42\times 0.68\times 0.05\times \text{P})$$ (2)

where slope is the rate of change (in absorbance units min^-1^) at 490 nm in undiluted homogenate calculated from at least two dilutions, 60 converts from minutes to hours, 0.25 is the reaction mixture volume (ml), 1.42 is the molar equivalent conversion of INT to μl O_2_, 0.68 is a path length correction factor, 0.05 is the homogenate volume (ml) and P is the protein concentration of the homogenate (mg ml^-1^). The conversion factor of 1.42 is derived from the term 15.9/(22.4/2) where 15.9 is the mM absorptivity of INT in the reaction mixture, 22.4 is the volume (μl) of 1 μmol O_2_ and 2 is the μmol INT-formazan produced equivalent to 1 μmol O_2_. Specific ETS activity (μl O_2_ hr^-1^ mg protein^-1^) was corrected for *in situ* temperature using the Arrhenius equation [36] with an *E*_*a*_ of 62.8 kj mol^-1^ [40].

### Zooplankton respiration rates

To convert spETS to daily respiration rates, R (mg C m^-3^ day^-1^), we used the equation:

R=([spETS]×PROT×0.5×0.97×[12/22.4]×24×DW)/1000 (3)

where PROT is the ratio of protein:dry weight as determined from equivalent AARS samples, 0.5 is the respiration rate (R): ETS ratio [41], 0.97 is the respiratory quotient [42], 12 is the weight (in μg) of 1 μmol C, 22.4 is the volume (μL) of 1 μmol O_2_, 24 converts from hours to days, DW is the dry weight of zooplankton (mg DW m^-3^) and 1000 converts from μg C to mg C.

### Zooplankton growth rates

Our samples were dominated by small copepods of the calanoid family Paracalanidae and the cyclopoid family Oithonidae [32]. To convert spAARS to growth rate (*G*), we applied two published relationships between spAARS and directly measured *G*, one of which applied to a calanoid and one to a cyclopoid copepod, both conducted at temperatures between 12 and 28°C. The calanoid relationship was established on the basis of experiments using nauplii of the copepod *Paracartia grani*, Eq (6) of Herrera et al. [43]:

G=0.13+0.007×spAARS (4)

For comparison, we also applied Equation (10) of Yebra et al. [44] developed for nauplii and copepodites of the cyclopoid *Oithona davisae*:

$$\text{spAARS}=24.35\times {\text{e}}^{5.51\text{G}}$$ (5)

For the spETS assays, we first calculated R (mg C m^-3^ d^-1^) according to Eq (3), and then *G* according to Ikeda and Motoda [45]:

G=0.75×R (6)

### Zooplankton production and grazing rates

We reasoned that the > 150 μm fraction best represented the mesozooplankton component of the zooplankton, since the 73–150 μm fraction would contain mostly copepod nauplii generally considered part of the microzooplankton. We also consider the > 150 μm fraction less contaminated by non-living seston and phytoplankton, and that the microzooplankton component of the samples would be unrealistically skewed toward more robust forms (i.e. that more delicate protistan microzooplankton would be undersampled in our net samples compared to, for example, crustacean nauplii). We did not include the multinet samples in these calculations because of the difference in mesh size (100 μm).

For the AARS assays we calculated *G* on the basis of Eqs (4) and (5). Volume-specific zooplankton production (ZP, mg C m^-3^ d^-1^) was then calculated as the product of each estimate of *G* and the directly measured C-specific biomass of zooplankton (mg C m^-3^). Similarly, for the ETS assays we calculated ZP as the product of C-specific biomass and *G* according to Eq (6).

We assessed the community potential ingestion (I; mg C m^-3^ d^-1^) from respiration rates (R), assuming an assimilation efficiency of 70% and a gross growth efficiency of 30% [45]:

I=0.75×R/(70-30)-2.5×R (7)

### Statistical methods

For each location, the effects of mesh size, cruise and their interactions on spETS and spAARS were explored via linear models in a Bayesian framework using JAGS [46] interfaced through R [47] and the R2jags [48] package (S1 Appendix). Models also included the covariates of protein (log transformed), chlorophyll (log transformed) and temperature in an attempt to reduce residual uncertainty. Non-informative normal priors were specified for the intercept and non-informative multivariate normal priors were specified for both the main effect and covariate parameters. Half-Cauchy (scale = 25) priors were specified for the variance [49]. A total of 10,000 Gibbs sampling iterations were performed across 3 chains with a burnin of 1,000 and thinning rate of 10, resulting in a total of 2,700 collected samples. Chain mixing and convergence were assessed via traceplots, autocorrelation and Gelman-Rubin diagnostics (all scale reduction factors less than 1.005).

The relative influences of the main effects of mesh, cruise and their interactions as well as the covariates (protein, chlorophyll *a* and temperature) were assessed via finite-population standard deviations [50]. Pairwise comparisons of mesh within each cruise and vice versa were derived from specific contrasts on the posteriors and all inferences about specific differences (effects) were based on 95% Bayesian UIs for modelled higher posterior density (HPD) median effects.

## Results

### Environmental context

**Fig 2:**
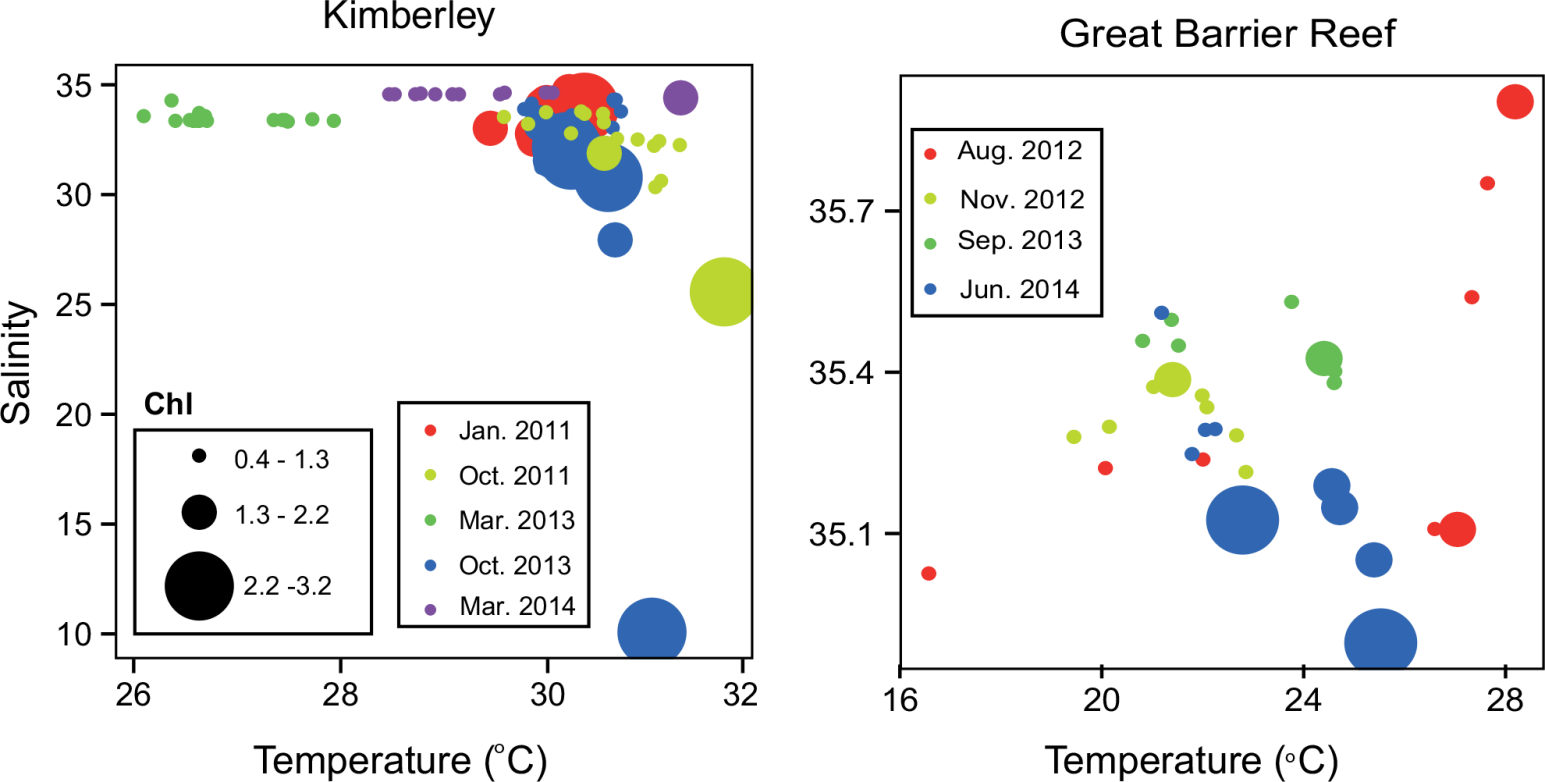
Temperature-Salinity-Chlorophyll diagrams for stations where enzyme assays were conducted. The numbers in this figure represent the means of temperature, salinity and fluorescence from the CTD casts over the full range of the water column. The hydrography for the Kimberley samples is further described in McKinnon et al. [32].

Water temperatures in the Kimberley ranged from 26.1°C to 31.7xC, generally exceeding those in the GBR (16.6°–28.2°C; Fig 2). Some GBR samples had low mean temperatures as a result of casts that extended below the thermocline in deeper water at multinet stations. The range of salinities was also wider in the Kimberley than in the GBR (10.1–34.7 vs 34.9–35.9), since that study spanned the full extent of coastal inlets, including three cruises in the wet season in conditions of high freshwater input. Similarly, chlorophyll *a* concentrations ranged between 0.39 and 3.17 xμg L^-1^ in the Kimberley, but were generally lower in the GBR (0.11–0.58 μg L^-1^; Fig 2).

### Plankton biomass

The dry weight per unit volume of 73–150 μm Kimberley samples was ~9-fold higher than in the GBR, but only 5–6-fold higher in the >150 μm and >350 μm fractions (Table 2). However, in terms of N, which is possibly the best indicator of living plankton biomass, the ratios of all size fractions were 3-4-fold higher in the Kimberley than in the GBR. The C:N ratio of plankton in each size fraction in both locations were remarkably similar. We interpret these results as indicative of higher zooplankton abundance in Kimberley waters, but that the higher values of dry weight of seston in the Kimberley indicates a greater load of inorganic seston than in the GBR. Protein concentrations were on average 3-fold higher in the Kimberley than in the GBR, and the >350 μm fraction was anomalously high in the Kimberley.

**Table 2:** Mean values (± standard deviations) of zooplankton dry weight (DW, mg m^-3^), Carbon (C, mg m^-3^), Nitrogen (N, mg m^-3^) and Protein (mg m^-3^) aggregated over all stations in each of the three size fractions sampled by the bongo net, with their ratios by weight.

Mesh	DW	C	N	Protein	C:DW	N:DW	C:N	Protein:DW
**Great Barrier Reef**								
**73–150μm**	7.62 (±8.42)	1.65 (±1.67)	0.36 (±0.37)	0.326 (±0.33)	0.217	0.047	4.601	0.043
**>150 μm**	13.06 (±12.47)	3.48 (±2.45)	0.88 (±0.66)	1.057 (±1.16)	0.267	0.067	3.957	0.081
**>350 μm**	8.99 (±12.12)	2.31 (±2.54)	0.58 (±0.58)	0.557 (±0.52)	0.257	0.065	3.968	0.062
**Kimberley Coast**								
**73–150μm**	66.25 (±86.25)	5.79 (±5.99)	1.27 (±1.43)	1.080 (±1.44)	0.087	0.019	4.542	0.016
**>150 μm**	81.99 (±271.85)	14.62 (±42.03)	3.63 (±11.45)	3.509 (±9.15)	0.178	0.044	4.025	0.043
**>350 μm**	41.36 (±265.99)	7.26 (±31.11)	1.94 (±8.53)	3.055 (±17.16)	0.175	0.047	3.749	0.074

### Kimberley zooplankton enzyme assays

Over all cruises and stations, spAARS activity ranged between 25.64–292.84 (mean 88.44) nmol PPi hr^-1^ mg protein^-1^ in the 73–150 μm size fraction, 11.34–278.71 (mean 132.22) nmol PPi hr^-1^ mg protein^-1^ in the >150 μm fraction, and 24.96–355.12 (mean 147.62) nmol PPi hr^-1^ mg protein^-1^ in the >350 μm fraction. Specific ETS activity ranged between 29.2 and 634.4 (mean 184.6) μL O_2_ hr^-1^ mg protein^-1^ in the 73–150 μm size fraction, 18.7–548.0 (mean 148.8) μL O_2_ hr^-1^ mg protein^-1^ in the >150 μm fraction, and 5.7–182.5 (mean 92.2) μL O_2_ hr^-1^ mg protein^-1^ in the >350 μm fraction.

**Fig 3:**
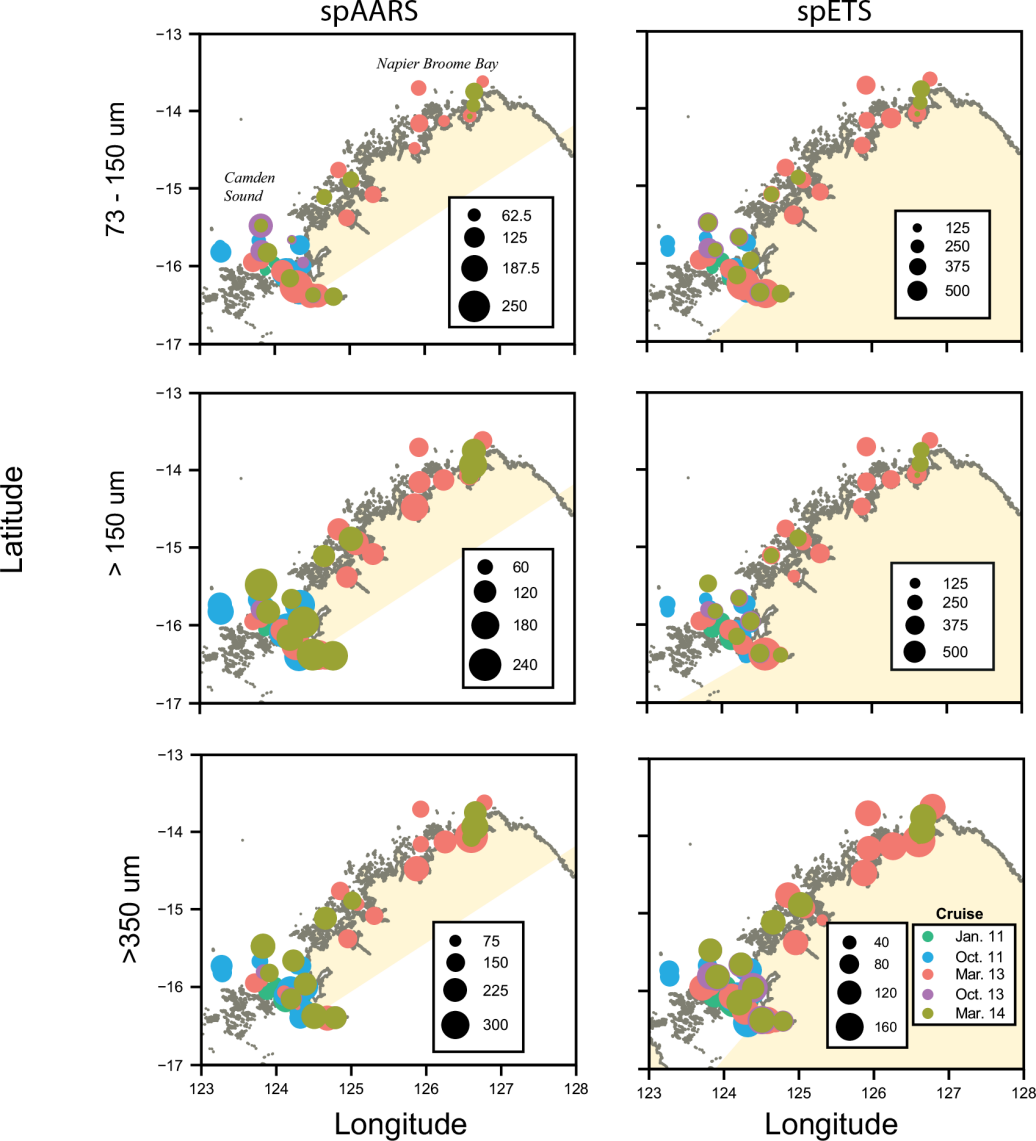
Enzyme activities (spAARS: nmol PPi hr^-1^ mg protein^-1^; spETS: μL O_2_ hr^-1^ mg protein^-1^) in the Kimberley region (see Table 1).

Highest values of both spAARS and spETS activity in the 73–150 μm and >150 μm fractions were observed in the macrotidal Camden Sound region, whereas there appeared to be little spatial difference in enzyme activity in the >350 μm fraction (Fig 3). Tidal range varied across our study region, from macro to mesotidal towards the east, and our sampling occurred during the spectrum of the spring-neap cycle. Therefore our initial impression of these data was that highest values of enzyme activity occurred in the areas of most tidal energy, but statistical comparisons of tidal range at the time of sampling with enzyme activities demonstrated this not to be the case.

**Fig 4:**
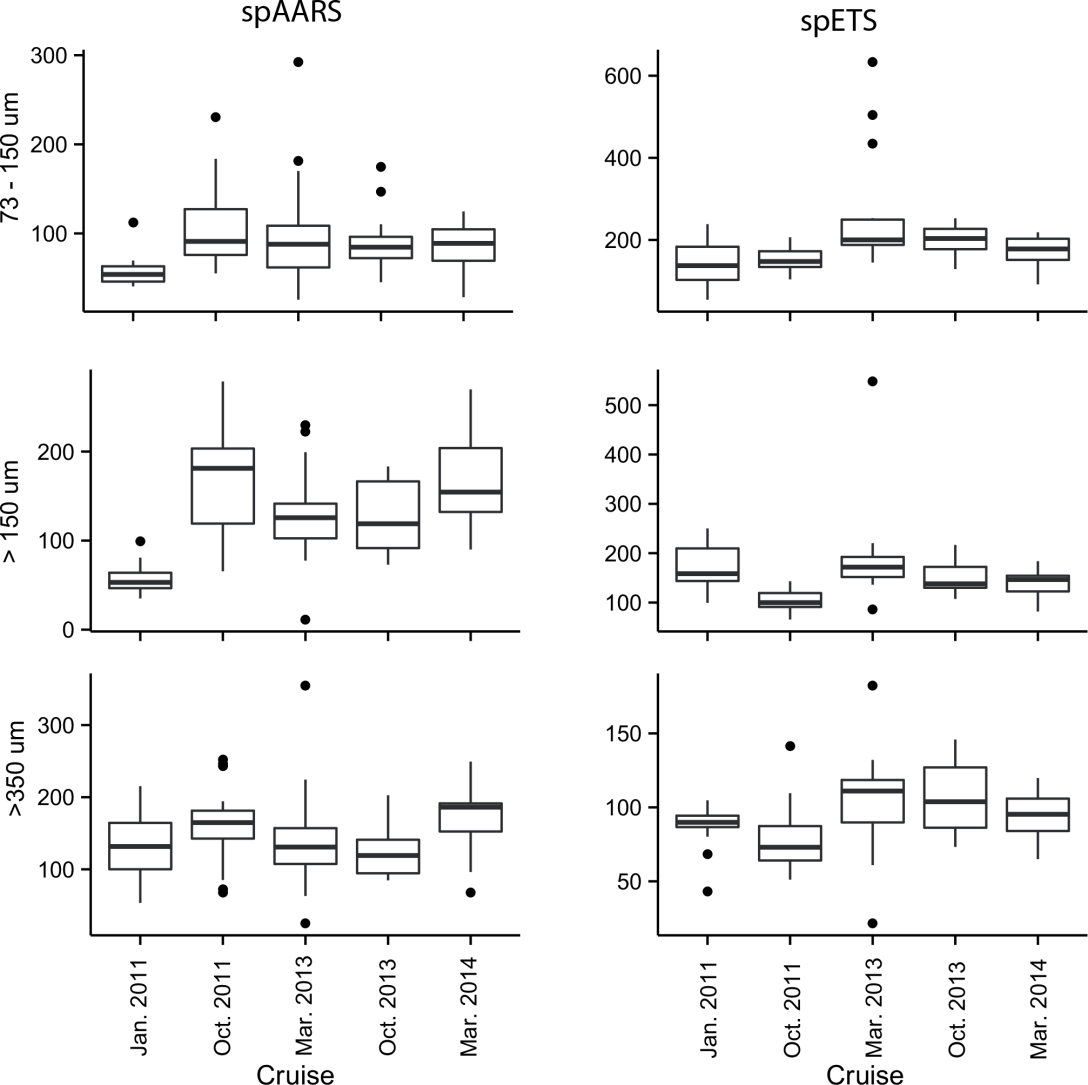
spAARS activity (nmol PPi hr^-1^mg protein^-1^) and spETS activity (μL O_2_ hr^-1^ mg protein^-1^) in 3 size fractions (73–150 μm, >150 μm and >350 μm) of zooplankton samples collected from 5 cruises to the Kimberley coast, contrasting cruise differences.

Enzyme activities on each cruise were quasi-normally distributed, but most cruises had some anomalously high outliers (Fig 4). We interpret these outlying values as real values rather than analytical artefacts; for example the outlying high spETS rates in the 73–150 μm and >150 μm size fractions in March 2013 were all from Walcott Inlet, an area of high primary production in which the zooplankton samples used for enzyme assays may have contained sufficient phytoplankton biomass to inflate our measurements. Similarly, the anomalously high spETS and spAARS rates in the >350 μm size fraction in February 2013 were from Napier Broome Bay, where the copepod *Microsetella* and the pteropod *Creseis* occurred in great abundance.

Our analysis of the enzyme data using Bayesian linear models estimated finite-population standard deviations, considered the equivalent of ANOVA sums of squares. For spAARS, size fraction accounted for 30% of the variation, cruise 24% and the interaction of these terms 13% (Fig T in S1 Appendix). The covariates (protein biomass, temperature and chlorophyll) each accounted for <2% of the variance; 28% of the variance remained unexplained. For spETS, size fraction accounted for 23% of the variation, cruise 21% and the interaction of these terms 17% (Fig R in S1 Appendix). Temperature accounted for 4% of variance, while protein biomass and chlorophyll *a* accounted for <0.5%. Overall, 33% of the variance remained unexplained. Separate regression analyses of >150 μm size fraction spETS activities on both temperature and salinity were significant (p<0.001 and p<0.01 respectively) despite explaining a low proportion of the variance (R^2^ < 0.12). Since these effects were themselves correlated to cruise periods it is likely that their contribution was absorbed within the main effect of cruise in our Bayesian model.

**Fig 5:**
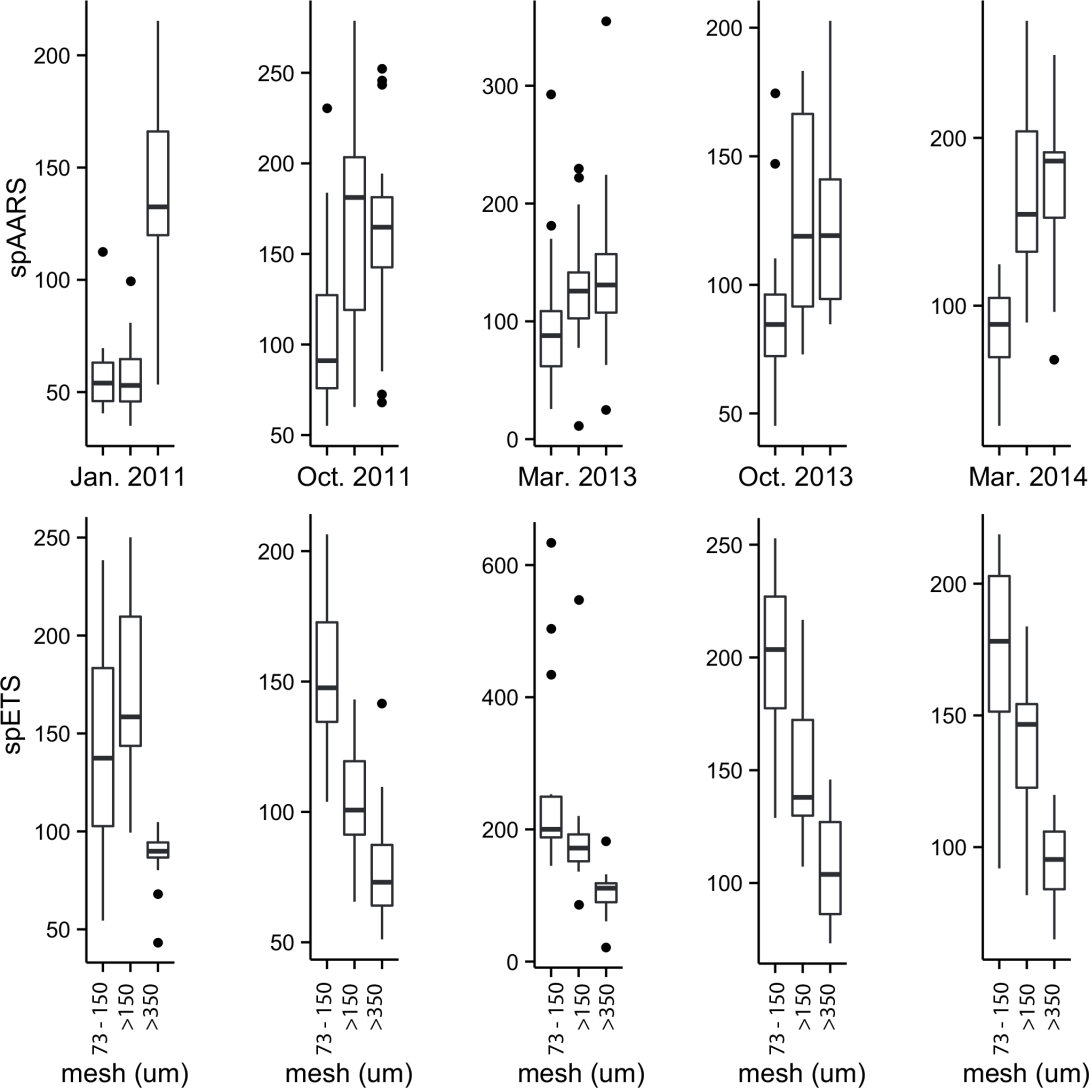
spAARS activity (nmol PPi hr^-1^mg protein^-1^) and spETS activity (μL O_2_ hr^-1^ mg protein^-1^) in 3 size fractions (73–150 μm, >150 μm and >350 μm) of zooplankton samples collected from 5 cruises to the Kimberley coast, contrasting differences between size fractions.

Comparison of the spAARS data grouped by size fraction (Fig 5) showed that activity in the >350 μm size fraction was always greater than in the 73–150 μm size fraction, with the activity in the >150 μm size fraction similar to that of either the 73–150 μm or >350 μm size fraction. Specific AARS activity in the >150 μm and >350 μm size classes was similar on all cruises and plausibly higher than the 73–150 μm size fraction except during the wet season of 2011, when 73–150 μm and >150 μm size fractions were similar (Fig U in S1 Appendix).

Specific AARS activity in the 73–150 μm and >150 μm size fractions during the wet season of 2011 was plausibly lower than all others (Fig U in S1 Appendix). This effect, though present, was less clear in the >350 μm size fraction. Pairwise comparisons of other cruises, though occasionally showing plausible differences, showed no clear overall pattern.

Comparison of spETS data grouped by size fraction (Fig 5) showed the opposite pattern to that observed from spAARS; with one exception (the wet season of 2011) the highest values were seen in the 73–150 μm size fraction, decreasing successively in the two larger size fractions. Moreover, differences in pairwise comparison of size classes were plausibly different in all but 3 cases: the 73–150 μm and >150 μm size classes in the wet season of 2011 and in the wet season of 2014, and the >150 μm and >350 μm size classes in the dry season of 2011 (Fig S in S1 Appendix).

Within the 73–150 μm size fractions, pairwise comparisons of cruises were only plausibly different for the wet season of 2011 compared to the dry season of 2011 and the wet season of 2013, and the wet season of 2014 compared to the wet season of 2013 and the dry season of 2013 (Fig S in S1 Appendix). In the >150 μm size fraction, the dry season of 2011 was plausibly lower than in the wet season of 2013, and the wet season of 2014 was plausibly lower than both the dry season of 2011 and the wet season of 2013. There were no differences in the >350 μm size fraction.

### Great Barrier Reef zooplankton enzyme assays

Over all cruises and stations, spAARS activity ranged between 24.1–224 (mean 71.72) nmol PPi hr^-1^ mg protein^-1^ in the 73–150 μm size fraction, 23.7–246 (mean 82.02) nmol PPi hr^-1^ mg protein^-1^ in the >150 μm fraction, and 17.7–156.45 (mean 83.82) nmol PPi hr^-1^ mg protein^-1^ in the >350 μm fraction. Specific ETS activity ranged between 14.9 and 173.4 (mean 86.5) μL O_2_ hr^-1^ mg protein^-1^ in the 73–150 μm size fraction, 34.1–137.8 (mean 88.3) μL O_2_ hr^-1^ mg protein^-1^ in the >150 μm fraction, and 19.1–134.9 (mean 71.3) μL O_2_ hr^-1^ mg protein^-1^ in the >350 μm fraction.

**Fig 6:**
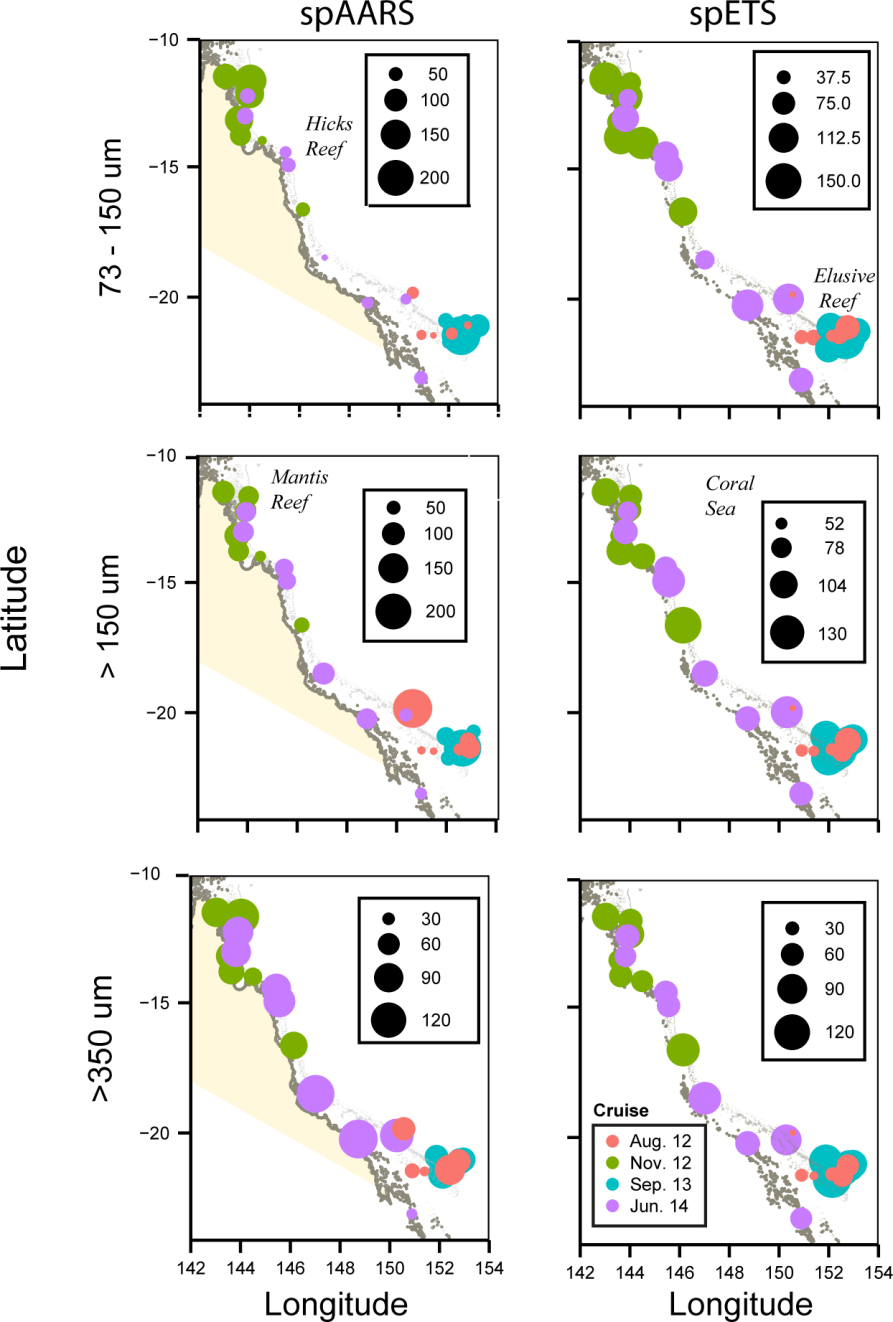
Enzyme activities (spAARS: nmol PPi hr^-1^ mg protein^-1^; spETS: μL O_2_ hr^-1^ mg protein^-1^) in the Great Barrier Reef region (see Table 1).

Highest values of spAARS activity in the >73–150 μm fraction occurred in the far northern GBR and around Elusive Reef, and in the >150 μm size fraction it was greatest in the Coral Sea off Hicks Reef and adjacent to Elusive Reef (Fig 6). Specific AARS activity in the >350 μm fraction and spETS activity in all size fractions did not show noticeable spatial variation.

**Fig 7:**
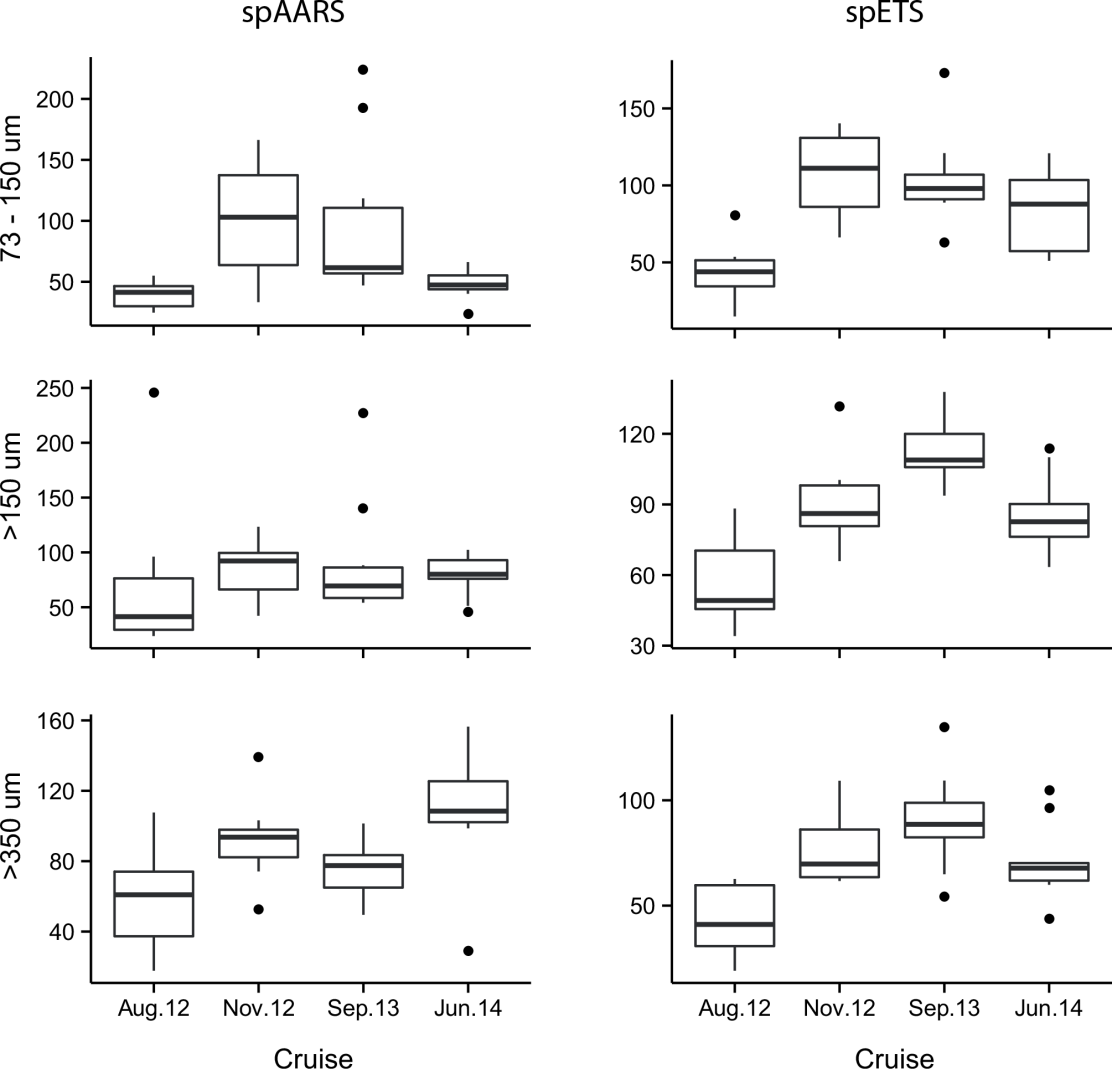
spAARS activity (nmol PPi hr^-1^ mg protein^-1^) and spETS activity (μL O_2_ hr^-1^ mg protein^-1^) in 3 size fractions (73–150 μm, >150 μm and >350 μm) of zooplankton samples collected during 4 cruises in the Great Barrier Reef, contrasting cruise differences.

The largest source of explained variation in enzyme activities was between cruises (Fig 7). For spAARS, cruise accounted for 25% of the variance, size fraction 12%, and the interaction of these terms 19% (Fig P in S1 Appendix). However, the majority of the variance (40%) was unexplained by the model. The covariates (protein biomass, temperature and chlorophyll) each accounted for <1% of the variance. For spETS, cruise accounted for 51% of variance (Fig N in S1 Appendix), there was comparatively little effect of size fraction (6% of variance), and the environmental covariates had almost no influence (<2%). There was a large proportion of variance that was unexplained by the model (26%).

**Fig 8:**
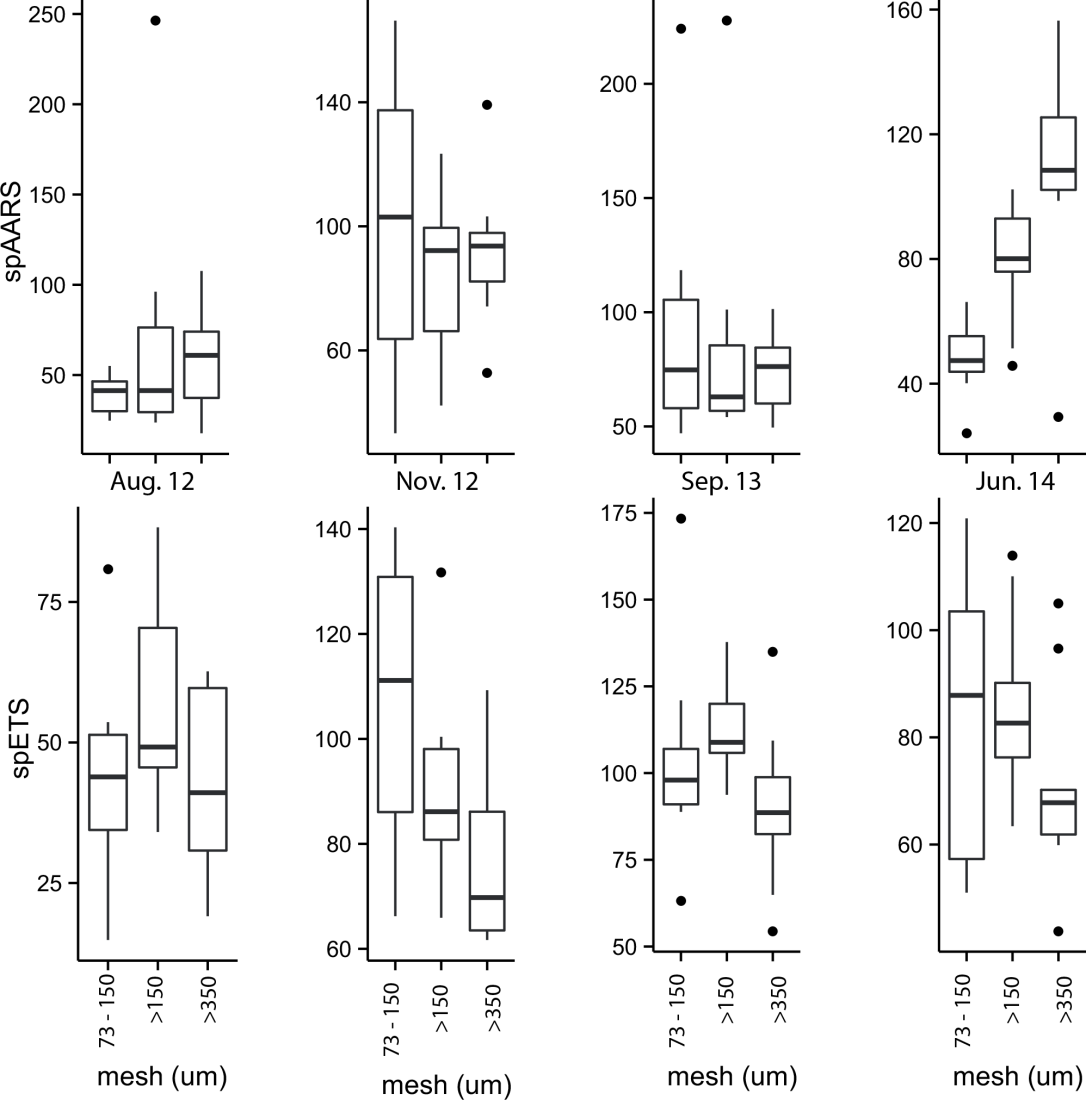
spAARS activity (nmol PPi hr^-1^ mg protein^-1^) and spETS activity (μL O_2_ hr^-1^ mg protein^-1^) in 3 size fractions (73–150 μm, >150 μm and >350 μm) of zooplankton samples collected during 4 cruises in the Great Barrier Reef, contrasting differences between size fractions.

Comparison of the spAARS data grouped by size fraction (Fig 8) showed that in only one case was there a plausible difference; for the 73–150 μm size fraction contrasted with the >350 μm size fraction in June 2014 (Fig Q in S1 Appendix). In contrast, spETS activities in specific size fractions were all plausibly different except in 4 cases: the 73–150 μm size fraction in November 2012 and September 2013, and for both the >150 μm and >350 μm fractions in November 2012 and June 2014 (Fig O in S1 Appendix).

#### Vertical variation

**Fig 9:**
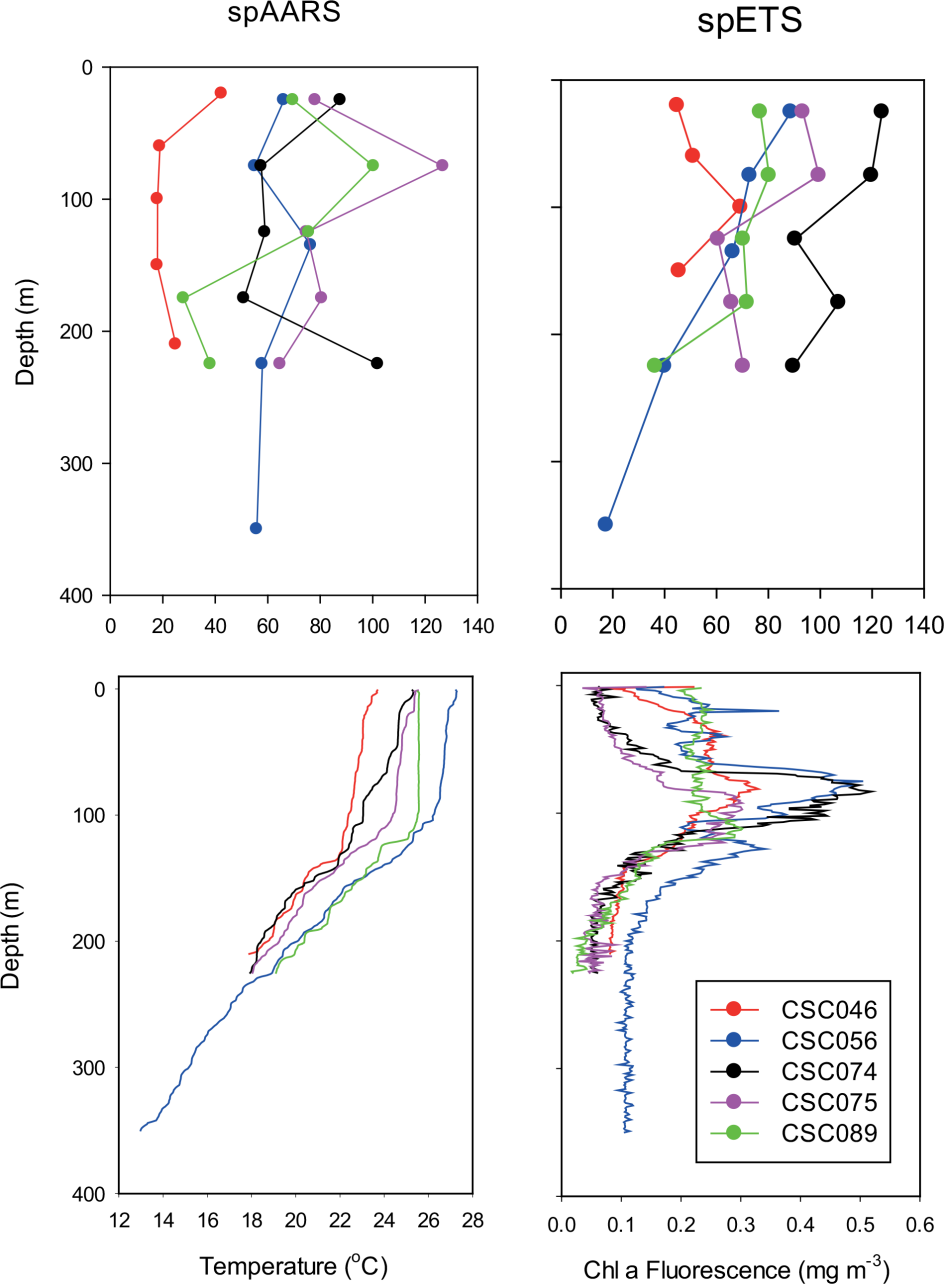
spAARS activity (nmol PPi hr^-1^ mg protein^-1^) and spETS activity (μL O_2_ hr^-1^ mg protein^-1^) in 100 μm zooplankton samples collected in discrete depth strata in Coral Sea waters adjacent to the Great Barrier Reef, contrasted with water column structure in terms of temperature and chlorophyll fluorescence.

At five stations in the GBR we made vertically stratified measurements of >100 μm zooplankton enzyme activities (Fig 9). The water column was stratified with respect to temperature on each occasion, with a thermocline at about 100 m. There was a well-defined chlorophyll maximum just above the thermocline on all occasions except CSC089, when chlorophyll was uniformly high throughout the mixed layer. Enzyme activities for depth strata of approximately 50 m depth intervals were broadly similar (Fig 9). At CSC056 broader strata were sampled with the deepest at 300–400 m. Enzyme activities appeared independent of either temperature or chlorophyll *a* concentration and in only one case–that of spETS at CSC056 –was there a clear indication of decrease in enzyme activity with depth.

### Zooplankton respiration, growth and production

We calculated zooplankton respiration from the spETS measurements according to Eq (3). ZP was then calculated as the product of C-specific biomass and *G* derived from R according to Eq (6), as well as from the product of C-specific biomass and *G* calculated from spAARS according to Eqs (4) and (5) (Table 3). On average, *G* calculated on the basis of Eq (4) was 3.7 times higher than that calculated on the basis of Eq (5). The estimate of ZP using *G* from Eq (4) was on average 6.3 times higher than that estimated from R, whereas ZP estimated using *G* from Eq (5) was on average 1.7 times higher than that estimated from R (Table 3).

**Table 3:** Summary of biomass (mg C m^-3^), respiration (R, mg C m^-3^ d^-1^), growth (*G* d^-1^) and zooplankton production (ZP, mg C m^-3^ d^-1^) estimates for each cruise based on the >150 μm size fraction. Growth has been calculated according to the empirically derived relationships between spAARS activities and independently estimated *G* for *Paracartia grani* (Eq 4) and for *Oithona davisae* (Eq 5). The subscripts Para and Oith refer to calculations made on each of these bases, respectively. ZP has been calculated 3 ways for comparison; on the basis of R (Eq 3), and on the basis of each estimate of *G*. The numbers are means and standard deviations of each set of n estimates for each cruise.

Cruise	n	Biomass	R	*G*_Para_	*G*_Oith_	ZP_R_	ZP_Para_	ZP_Oith_
**Great Barrier Reef**								
**Aug. 2012**	8	2.54	0.24	0.63	0.14	0.18	1.55	0.32
		(±1.53)	(±0.25)	(±0.52)	(±0.14)	(±0.18)	(±1.45)	(±0.37)
**Nov. 2012**	8	4.37	1.16	0.73	0.22	0.87	3.39	1.03
		(±3.19)	(±0.88)	(±0.18)	(±0.06)	(±0.66)	(±2.91)	(±0.90)
**Sep. 2013**	10	2.76	0.38	0.76	0.22	0.28	2.02	0.58
		(±1.96)	(±0.35)	(±0.38)	(±0.08)	(±0.26)	(±1.47)	(±0.42)
**Jun. 2014**	8	4.34	0.57	0.68	0.21	0.43	2.95	0.89
		(±2.66)	(±0.43)	(±0.13)	(±0.05)	(±0.32)	(±2.08)	(±0.65)
**Kimberley Coast**								
**Jan. 2011**	18	15.10	4.50	0.53	0.15	3.37	7.87	2.18
		(±8.56)	(±14.58)	(±0.11)	(±0.04)	(±2.72)	(±4.29)	(±1.23)
**Oct. 2011**	21	1.96	0.60	1.32	0.34	0.45	2.67	0.68
		(±0.81)	(±0.31)	(±0.40)	(±0.07)	(±0.24)	(±1.57)	(±0.34)
**Mar. 2013**	21	30.01	9.06	1.06	0.29	6.80	30.25	8.51
		(±86.54)	(±24.46)	(±0.36)	(±0.11)	(±18.35)	(±79.53)	(±24.07)
**Oct. 2013**	12	7.90	1.41	1.02	0.29	1.06	7.77	2.24
		(±3.38)	(±0.78)	(±0.29)	(±0.06)	(±0.59)	(±2.85)	(±0.79)
**Mar. 2014**	18	16.28	2.70	1.33	0.34	2.02	20.52	5.29
		(±13.48)	(±1.89)	(±0.39)	(±0.06)	(±1.42)	(±17.01)	(±4.42)

There was remarkably little variation in R, *G* or ZP between the GBR cruises (Table 3). In the Kimberley, biomass and R were lower in the cooler dry season cruises (October 2011, October 2013) than on the other cruises that all occurred in the warmer wet season (Table 3). Paradoxically, there was no such pattern for *G* except for January 2011 being lower than other cruises, reflecting the lower enzyme activities observed on that cruise, discussed above. The March 2013 cruise was characterized by very high variance in our estimates, possibly because of the diversity of habitats sampled on that cruise, which spanned the entire Kimberley coast.

## Discussion

This study has made comparative measurements of zooplankton growth and respiration on the west (Indian Ocean) and east (Pacific Ocean) coasts of tropical Australia, and found rates 2–4-fold higher in the west, despite broadly similar temperatures and nutrient regimes [1]. Remarkably, there were no indications of major changes in enzyme activities between wet and dry seasons, even though temporal effects were greater than spatial effects. Our measurements of zooplankton spAARS and spETS activity comprise the largest dataset available for the tropics, and the measurements from the Kimberley were taken at water temperatures in excess of any other published study. Stations occupied in the Kimberley were predominately coastal, since our intention was to document coastal plankton in the many inlets and embayments along this complex coastline. The community composition of mesozooplankton from the Kimberley is dominated by copepods, particularly small cyclopoid copepods of the genus *Oithona* and small paracalanid copepods of the genera *Bestiolina* and *Parvocalanus*, with occasional high abundance of the harpacticoid genus *Microsetella* [32]. Stations occupied in the GBR for this study were designed to document planktonic processes at the outer margin of the GBR adjacent to the Coral Sea, but our sample set includes many within the reef matrix or reef lagoon *en route* to the outer reef sites. The community composition of mesozooplankton from the GBR is similar to that of the Kimberley at the level of genus [15], though there are differences in species dominance. There is also a much greater representation of larvaceans and copepods of the families Corycaeidae and Oncaeidae in waters of the GBR [15].

Our enzyme assays had some extreme outliers. In the Kimberley, these mostly originated from a series of stations occupied in Walcott Inlet in the wet season of 2013 where primary production was as high as 4.7g C m^-2^ d^-1^ (Furnas, pers. comm.), but also in the >350 μm size fraction from a station located in Napier Broome Bay on the same cruise. Plankton abundance within Napier Broome Bay was 10-fold higher than in any other Kimberley location [32]. This peak in abundance occurred as a result of population increases of the small harpacticoid copepod *Microsetella* and the much larger pteropod *Creseis aciculata*. Though little is known of pteropod growth rates, they have been reported to prosper in nutrient-enriched conditions [51]. As is the case for other mucous-net feeders such as larvaceans, pteropods may have the capacity for rapid growth and be responsible for the extreme values of our assays. High outliers also occurred in the GBR enzyme data, especially in the >150 μm fraction in August 2012 and September 2013. In two of these three samples there were high numbers of larvaceans, which are thought to have the fastest growth rates of any metazoans [52].

In the seasonally structured Kimberley data set there were no consistent seasonal trends in the enzyme data. It may be that changes in the zooplankton communities or trophic conditions at the time of sampling modulate community rates of growth and respiration. For example, the October 2011 cruise had the highest spAARS values in all three size fractions, coinciding with high phytoplankton growth rates on that cruise (Furnas, pers. comm.). Specific ETS activity in the >150 μm fraction containing the dominant copepod fraction of the mesozooplankton was higher during wet season cruises than during the dry season cruises, which seems reasonable since respiration should scale with temperature. However, the October 2011 cruise had the highest spAARS activity, but low spETS activity. These results seem inconsistent and suggest that the activities of these two enzyme systems are not closely coupled.

Similarly, in the GBR no strong temporal trends occurred in the enzyme data except that the August 2012 cruise was consistently lower than the other cruises for both spAARS and spETS in all three size fractions. Admittedly, our GBR data do not include any wet season measurements, and seasonality is the source of the greatest contrasts in microbial respiration and primary production in the GBR [8]. The seasonal minimum of zooplankton abundance on the GBR is at the end of August [53]. This is consistent with our finding of low enzyme activities in August 2012, but inconsistent with our finding of plausibly higher activities in September 2013.

Specific ETS activity was plausibly different between the 3 size classes of zooplankton in the Kimberley samples, with highest rates occurring in the 73–150 μm fraction and lowest in the >350 μm fraction. In contrast, however, spAARS activities tended to be greater in the larger size fractions. The reason for this disparity is unclear, since both respiration and growth scale negatively with body size [54]. In the GBR samples the same trends were evident, but were less marked because of the smaller sample size (i.e. fewer stations were occupied on the GBR than in the Kimberley). Measurements of size fractionated ETS and glutamate dehydrogenase (related to excretion) in zooplankton from coastal, transitional and offshore stations in the Benguela upwelling system did not suggest consistent differences in enzyme activities in the size classes measured (100–200 μm, 200–500 μm, 500–1000 μm, >1000 μm) [55]. In the Mediterranean, comparison of ETS of zooplankton in 3 size classes (53–200 μm, 200–500 μm, >500 μm) showed that where significant differences in biomass-specific potential respiration were found the small fraction had the highest values [56], as was the case in our study. The reason why our spAARS values are highest in the largest size fractions is unclear.

### Zooplankton respiration

We have measured specific ETS activity, sometimes referred to as “potential respiration”, ɸ, an index of the biochemical machinery available to undertake respiration, and which represents an upper limit to respiration. Our measurements of ɸ in bulk zooplankton in all three size fractions were 2-3-fold higher in the Kimberley than on the GBR. Nutritional level, activity and behavioral shifts combine to determine the actual respiratory oxygen consumption [56]. In fact, there is sometimes an excellent relationship between ɸ and measured respiration (e.g. [38]), presumably when resources are not limiting. However, Herrera et al. [56] modelled log ETS against log biomass and found exponents considerably lower than 0.75 (i.e. the ¾ power predicted by Kleiber’s law)—between 0.35 and 0.65 in 3 size classes (53–200 μm, 200–500 μm, >500 μm) of western Mediterranean zooplankton—and made the case that this was indicative of food limitation, despite ETS activity apparently accounting for 19.7% of primary production. In our case, regressions of log spETS against log biomass (as mg protein m^-3^) had slopes of 0.81 and 0.96 for Kimberley and GBR samples, respectively. Applying the logic of Herrera et al. [56], at our study sites zooplankton do not appear to be severely food-limited in contrast to what has been suggested by earlier studies of copepod growth and egg production [24].

Our measurements of ɸ from the five vertically stratified stations from Coral Sea waters adjacent to the outer GBR gave no indication that there is any difference in respiration with depth, at least over the scale of our sampling (<400 m depth) and within the constraints of our admittedly small dataset. Similarly, there was little change in ETS activity with depth in the epipelagic zone of the Gulf of Mexico [57]. This similarity in epipelagic respiration may be attributed to vertical movement of plankton within the water column [58]. In the Benguela upwelling system, zooplankton ETS activity corresponded to the distribution of biomass [55], and the strongest gradients were cross-shelf despite some indication of vertical structure over the 150 m depth range sampled.

### Role in carbon cycling

Converting our oxygen-specific units to carbon-specific units, we calculated the mean volume-specific respiration rate for zooplankton >73 μm in size (i.e. summing the 73–150 μm and >150 μm size fractions) to be 0.70 mg C m^-3^ d^-1^ on the GBR, and 5.05 mg C m^-3^ d^-1^ in the Kimberley. Zooplankton abundance is similar in the Kimberley to that in the inshore waters of the GBR [15], so the higher respiration rate must come about as a combination of higher temperature and turbulence, and changing food availability. Assuming an average water depth on the GBR of 35 m [59] and an average depth for our Kimberley stations of 41 m, then the mean area-specific rates are 24 mg C m^-2^ d^-1^ on the GBR and 207 mg C m^-2^ d^-1^ in the Kimberley. Allowing for the differences in integration depths, these estimates appear reasonable when compared with mean mesozooplankton respiration rates for the open ocean of 144 mg C m^-2^ d^-1^ for equatorial waters between 10°N and 10°S, and 98 mg C m^-2^ d^-1^ for waters between 10°S and 50°S [60].

On the GBR, median respiration rates of whole water samples are 1.85 mmol O_2_ m^-3^ d^-1^ in the dry season and 2.87 mmol O_2_ m^-3^ d^-1^ in the wet season [8]. Assuming 4 months of wet season and 8 months of dry season, the seasonally averaged respiration rate is 2.19 mmol O_2_ m^-3^ d^-1^. Our spETS data correspond to means of 5.2, 16.5 and 8.0 μmol O_2_ m^-3^ d^-1^ in the 73–150 μm, >150 μm and >350 μm size fractions, respectively. Our value of ɸ for >73 μm zooplankton is therefore equivalent to only 1% of the seasonally averaged whole water respiration rate as above, in contrast to the contribution of 10 ± 8.5% of total respiration estimated for the 100–1000 μm size class of larval and adult mesozooplankton in the epipelagic ocean [61].

### Zooplankton grazing and production with respect to primary production

Applying Eq (7), we calculate that >150 μm zooplankton have ingestion rates of 1.7 and 12.6 mg C m^-3^ d^-1^ for the GBR and Kimberley, respectively. To estimate ZP of >150 μm zooplankton, we used the grand mean of estimates derived on the basis of the spETS and those of ZP derived on the basis of the two published spAARS-*G* relationships (Table 3). The resulting grand mean of >150 μm ZP on the GBR was 1.21 mg C m^-3^ d^-1^ and for the Kimberley was 6.78 mg C m^-3^ d^-1^.

In area-specific terms, we estimate zooplankton production on the GBR to be on the order of 42 mg C m^-2^ d^-1^ and 278 mg C m^-2^ d^-1^ in the Kimberley, assuming water depths of 35 and 41 m, respectively. Our enzyme-based methods have generated zooplankton production rates comparable to previous measurements, for example 151 mg C m^-2^ d^-1^ in Kaneohe Bay, Hawaii [62] and 400 mg C m^-2^ d^-1^ in the Eastern Agulhas Bank of the Benguela upwelling system [63]. Previous measurements of copepod production in Australian waters, based on artificial cohort experiments, were substantially lower than our estimate of ZP, averaging 12.6 mg C m^-2^ d^-1^ in both shelf break (75 m depth) and shelf (18 m depth) stations near NW Cape [23], and 2.6 mg C m^-2^ d^-1^ in the dry season and 8.5 mg C m^-2^ d^-1^ in the wet season in coastal waters of the GBR (20 m depth) [24]. These estimates differ from those of the present work in that they focussed solely on the copepod component of the zooplankton, but the range of volume-specific production estimates from these studies (0.13–0.70 mg C m^-3^ d^-1^) is an order of magnitude lower than our enzyme-based measurements. Given that copepods comprise ~80% of the mesozooplankton by number, this disparity points to methodological differences, possibly involving experimental artefacts inherent in incubation methods. We emphasize, however, that the previous estimates of zooplankton production based on artificial cohort experiments focussed solely on the small copepods dominant in these systems, whilst our enzyme methods were conducted on mixed plankton selected purely on the basis of the mesh size of our plankton nets.

What proportion of primary production do our estimates of grazing rates represent? Unfortunately, for our GBR samples there were no direct measurements of primary production on these cruises. However, we can assume a mean value of 730 mg C m^-2^ d^-1^ [8] resulting in a mean volume-specific primary production of 21 mg C m^-3^ d^-1^ assuming an average water depth of 35 m as above, in which case it appears that trophic transfer from primary producers to >150 μm zooplankton has an efficiency of about 6%, and that >150 μm zooplankton consume ~8% of primary production (applying Eq 7). For the Kimberley samples, similar primary production measurements are available for 29 of the stations at which zooplankton were collected (McKinnon unpublished). However, the calculation of the proportion of primary production consumed is complicated by the very shallow euphotic zone in Kimberley waters. We have made the assumption that turbulence arising from the huge tidal range of the Kimberley would mix primary producers (and hence available production) throughout the water column. On this basis, combining the directly measured, volume-specific primary production measured closest to the surface from those measurements (mean 177 mg C m^-3^ d^-1^, range of 10–481 mg C m^-3^ d^-1^) with ZP calculated at the same station, trophic transfer in the Kimberley (as above) is 4% efficient, and the > 150 μm zooplankton consumption is equivalent to 7% of the primary production in the surface stratum.

In northern Australia the southward extension of tropical waters favor the dominance of picoprokaryotes in the phytoplankton, though diatom blooms occur periodically on the east coast [64]. Unfortunately, the only available comparison of phytoplankton on the west and east coasts of Australia [64] did not consider the NW shelf, but waters of the Kimberley resemble those of the GBR in that they are also dominated by picoplankton [12]. The dominance of picoautotrophs, which because of their small size are not directly available to mesozooplankton, does not always result in lower grazing rates. Picoplankton coagulation into flocs can more efficiently entrain these small cells into food chains than would be expected via microbial pathways [65]. Quantification of mesozooplankton feeding on detrital flocs is challenging, yet these represent important biogeochemical pathways [66].

On the GBR there is concern about the input of nutrients from the land, usually during periods of flood, and the subsequent elevation in chlorophyll *a* [67,68]. What is virtually unknown is the way in which such flood plumes dissipate, and the fate of the organic material within them. Grazing has a critical role in the fate of oceanic blooms [69]. However, there have been few attempts to calculate grazing rates of zooplankton in northern Australian waters. The planktonic response to riverine plumes entering the GBR is dominated by small to medium sized diatoms that can out-grow their zooplankton grazers when not constrained by nutrient availability [10]. In the GBR lagoon copepods showed an immediate growth response to flood conditions and larvaceans showed a rapid increase in abundance, but it took about one month for calanoid copepod juveniles to respond in terms of abundance [53].

On the basis of the incorporation of radio-labelled particulate material, zooplankton at Davies Reef (GBR) cropped 30% of daily primary production by >2 μm phytoplankton [21], which is barely sufficient to cover their metabolic needs. To account for the deficit those authors concluded that most nutrition came from other sources such as detritus or other organic material originating from benthic communities, a conclusion also reached by others [70]. Some indication of the total ingestion rate of zooplankton can also be inferred from directly measured production. Zooplankton production efficiencies with respect to primary production estimated from the growth of artificial cohorts were <1% for the dominant copepod component of the zooplankton both on the GBR and in the vicinity of NW Cape, WA [24]. These estimates of zooplankton production are insufficient to explain the fate of primary production, since sedimentation seems to be a minor term [9,71]. Similarly, bottle feeding experiments failed to account for the metabolic demand of common copepods [72,22]. We conclude that the application of enzymatic indices of *in situ* growth or respiration results in much more reasonable estimates of the percentage of primary production grazed by >150 μm zooplankton (~7.5%). By way of comparison, mesozooplankton ingestion accounted for ~40% of primary production in the Arabian Sea [73], but on average mesozooplankton consume ~12% of global primary production [16].

Our study has produced the first realistic estimate of zooplankton grazing rates for the GBR, and by extension, for the Kimberley. However, we have been unable to resolve issues such as the unexpected higher rates of spAARS in the larger size fractions when compared to the smaller size fractions, and our enzyme-based growth and production rates from the Kimberley seem high in comparison with previous estimates, for example from the Benguela upwelling system [63]. However, when the high water temperatures and more estuarine nature of the Kimberley study sites are considered, our ZP estimate of 278 mg C m^-2^ d^-1^ appears reasonable and is supported by the highest productivity per unit chlorophyll *a* in Australian waters occurring on the NW shelf [1] and also the highest zooplankton biomass [74]. Placed in perspective of primary production rates, our enzyme-based measurements are similar to, though slightly lower than, global estimates of the percentage of primary production grazed by mesozooplankton [16]. Our estimates of zooplankton production are high when compared to previous estimates within Australia and to the comparatively few measurements elsewhere. Consequently, our study has not been able to offer an explanation for the mismatch between primary productivity and fish production. Perhaps the answer may lie in the temporal variability apparent in both primary (e.g. [2]) and zooplankton production [this study], and that these are not reliably sustained over time scales relevant to the growth of larval fish. For instance, Meekan et al. [75] concluded that environmental conditions in the days immediately after hatching determined growth rates during later life and were ultimately an important determinant of larval supply. In this scenario, early larvae coinciding with optimal food conditions may starve when production rates decline, and early larvae coinciding with sub-optimal food conditions may suffer higher mortality throughout larval life.

We have highlighted the very different pelagic productivity regimes between the NW and NE coasts of Australia, despite broad similarity in zooplankton community structure [15]. Our measurements considerably add to the number of assays of these enzymes made in tropical waters, and our wet season measurements from the Kimberley region were conducted in the highest ambient water temperatures of any published study (>30°C). Area-specific respiration by >73–150 μm zooplankton was ~7-fold higher in the Kimberley than on the GBR and ZP by >150 μm zooplankton 6-fold higher. We hypothesize that the stronger physical forcing on the NW shelf, together with high temperatures and turbulence, is the principal determinant of these differences.

## Supporting Information

S1 Appendix Figs A-U. Bayesian statistical analysis of data, including R code.
(PDF)
