# Supplementary material for: Zooplankton Growth, Respiration and Grazing on the Australian Margins of the Tropical Indian and Pacific Oceans
Source: PLoS One. 2015 Oct 15;10(10):e0140012. doi: 10.1371/journal.pone.0140012 (PMC4607465; doi:10.1371/journal.pone.0140012)
Supplement: S1 Appendix — Bayesian statistical analysis of data, including R code. (PDF) [file pone.0140012.s001.pdf]

Enzyme analysis

Murray Logan
September 26, 2015

Table of contents

1 Preparations 1
- 1.1 Libraries . . . 1
- 1.2 Read in data . . . 1
- 1.3 Data manipulations . . . 2

2 Exploratory data analysis 3

3 Analyses 18
- 3.1 GBR . . . 18
  - 3.1.1 ETS . . . 19
  - 3.1.2 AARS . . . 24
- 3.2 Kimberley . . . 29
  - 3.2.1 ETS . . . 29
  - 3.2.2 AARS . . . 34

4 References 39

1. Preparations

1.1. Libraries

```
> library(ggplot2)
> library(gridExtra)
> library(R2jags)
> library(plyr)
> library(xtable)
```

1.2. Read in data

```
> csc <- read.csv('data/CSCzymes.csv', strip.white=TRUE)
> str(csc)
```

```
'data.frame':   105 obs. of  8 variables:
 $ Station: Factor w/ 33 levels "CSC042","CSC043",..: 1 2 3 4 5 6 7 8 9 10 ...
 $ Cruise : int  1 1 1 1 1 1 1 1 2 2 ...
 $ Mesh   : int  73 73 73 73 73 73 73 73 73 73 ...
 $ Temp   : num  22.9 21 21.4 22 20.2 ...
 $ Chl    : num  0.238 0.215 0.271 0.228 0.154 ...
 $ Protein: num  0.1747 0.2828 0.0822 0.2548 0.0553 ...
 $ ETS    : num  63.8 157 183.7 232.6 217.7 ...
 $ AARS   : num  50.5 41.4 27.5 33.6 61.1 ...
```

```
> summary(csc)
```

```
    Station       Cruise           Mesh          Temp            Chl              Protein
 CSC075 : 9   Min.   :1.000   Min.   : 73   Min.   :16.57   Min.   :0.1094   Min.   :0.007117
 CSC042 : 3   1st Qu.:2.000   1st Qu.: 73   1st Qu.:21.40   1st Qu.:0.1532   1st Qu.:0.177098
 CSC043 : 3   Median :3.000   Median :150   Median :22.25   Median :0.2094   Median :0.394378
 CSC044 : 3   Mean   :2.571   Mean   :191   Mean   :23.03   Mean   :0.2262   Mean   :0.648345
```

```
CSC045 : 3   3rd Qu.:4.000   3rd Qu.:350   3rd Qu.:24.62   3rd Qu.:0.2652   3rd Qu.:0.810647
CSC046 : 3   Max.   :4.000   Max.   :350   Max.   :28.19   Max.   :0.5765   Max.   :5.696086
(Other):81                                                                  NA's   :2
     ETS              AARS
Min.   : 63.8   Min.   : 19.71
1st Qu.:260.9   1st Qu.: 55.09
Median :349.1   Median : 77.58
Mean   :345.6   Mean   : 87.98
3rd Qu.:440.7   3rd Qu.:106.83
Max.   :729.4   Max.   :273.69
NA's   :1
```

```
> kim <- read.csv('data/KIMzymes.csv', strip.white=TRUE)
> str(kim)
```

```
'data.frame':   267 obs. of  8 variables:
 $ Station: Factor w/ 89 levels "KIM002","KIM003",..: 1 1 1 2 2 2 3 3 3 4 ...
 $ Cruise : int  1 1 1 1 1 1 1 1 1 1 ...
 $ Mesh   : int  73 150 350 73 150 350 73 150 350 73 ...
 $ Temp   : num  30 30 30 30.4 30.4 ...
 $ Chl    : num  3.37 3.37 3.37 3.09 3.09 ...
 $ Protein: num  1.998 6.097 NA 0.724 3.772 ...
 $ ETS    : num  428 504 174 486 450 ...
 $ AARS   : num  47.9 38.9 59.3 48.7 57.7 ...
```

```
> summary(kim)
```

```
   Station        Cruise           Mesh          Temp            Chl              Protein
 KIM002 :  3   Min.   :1.000   Min.   : 73   Min.   :26.10   Min.   :0.3914   Min.   :  0.02508
 KIM003 :  3   1st Qu.:2.000   1st Qu.: 73   1st Qu.:28.52   1st Qu.:0.5476   1st Qu.:  0.48979
 KIM004 :  3   Median :3.000   Median :150   Median :29.96   Median :0.8060   Median :  1.00035
 KIM005 :  3   Mean   :2.876   Mean   :191   Mean   :29.37   Mean   :1.1126   Mean   :  2.57076
 KIM007 :  3   3rd Qu.:4.000   3rd Qu.:350   3rd Qu.:30.35   3rd Qu.:1.5562   3rd Qu.:  1.99886
 KIM008 :  3   Max.   :5.000   Max.   :350   Max.   :31.71   Max.   :3.3707   Max.   :161.92120
 (Other):249                                                                  NA's   :7
      ETS               AARS
 Min.   :  86.2   Min.   : 12.61
 1st Qu.: 397.2   1st Qu.: 83.72
 Median : 533.3   Median :126.69
 Mean   : 574.0   Mean   :136.98
 3rd Qu.: 707.8   3rd Qu.:182.47
 Max.   :2521.5   Max.   :394.57
 NA's   :5        NA's   :4
```

1.3. Data manipulations

```
> csc$Mesh <- factor(csc$Mesh)
> csc$Cruise <- factor(csc$Cruise)
>
> kim$Mesh <- factor(kim$Mesh)
> kim$Cruise <- factor(kim$Cruise)
```

2. Exploratory data analysis

```
> g1<-ggplot(csc, aes(y=ETS, x=rep(1,nrow(csc)), color=Mesh)) +
+     geom_jitter() +
+     geom_boxplot(aes(x=2),alpha=0.2)+
+         facet_wrap(~Cruise, scales='fixed',nrow=1)+theme_classic()+
+         scale_y_continuous('Activity of enzyme ETS')+
+         scale_x_continuous('')+theme(axis.text.x=element_blank(),axis.ticks.length=unit(0, 'cm'),
+                                  legend.position=c(0,1),legend.justification=c(0,1),
+                                  axis.title.y=element_text(vjust=2),plot.margin=unit(c(0,0,2,2),'lines'))
> g2<-ggplot(csc, aes(y=AARS, x=rep(1,nrow(csc)), color=Mesh)) +
+     geom_jitter() +
+     geom_boxplot(aes(x=2),alpha=0.2)+
+         facet_wrap(~Cruise, scales='fixed',nrow=1)+theme_classic()+
+         scale_y_continuous('Activity of enzyme AARS')+
+         scale_x_continuous('')+theme(axis.text.x=element_blank(),axis.ticks.length=unit(0, 'cm'),
+                                  legend.position=c(0,1),legend.justification=c(0,1),
+                                  axis.title.y=element_text(vjust=2),plot.margin=unit(c(0,0,2,2),'lines'))
>
> grid.arrange(g1,g2,nrow=2)
```

```
> g1<-ggplot(kim, aes(y=ETS, x=rep(1,nrow(kim)), color=Mesh)) +
+     geom_jitter() +
+     geom_boxplot(aes(x=2),alpha=0.2)+
+         facet_wrap(~Cruise, scales='fixed',nrow=1)+theme_classic()+
+         scale_y_continuous('Activity of enzyme ETS')+
+         scale_x_continuous('')+theme(axis.text.x=element_blank(),axis.ticks.length=unit(0, 'cm'),
+                                  legend.position=c(0,1),legend.justification=c(0,1),
+                                  axis.title.y=element_text(vjust=2),plot.margin=unit(c(0,0,2,2),'lines'))
> g2<-ggplot(kim, aes(y=AARS, x=rep(1,nrow(kim)), color=Mesh)) +
+     geom_jitter() +
+     geom_boxplot(aes(x=2),alpha=0.2)+
+         facet_wrap(~Cruise, scales='fixed',nrow=1)+theme_classic()+
+         scale_y_continuous('Activity of enzyme AARS')+
+         scale_x_continuous('')+theme(axis.text.x=element_blank(),axis.ticks.length=unit(0, 'cm'),
+                                  legend.position=c(0,1),legend.justification=c(0,1),
+                                  axis.title.y=element_text(vjust=2),plot.margin=unit(c(0,0,2,2),'lines'))
>
> grid.arrange(g1,g2,nrow=2)
```

```
> g1 <- ggplot(csc, aes(y=ETS, x=Temp, color=Mesh)) + geom_point()+facet_wrap(~Cruise, scales='fixed',nrow=1)+geom_smooth(method='lm')+theme_classic()
> g2 <- ggplot(csc, aes(y=AARS, x=Temp, color=Mesh)) + geom_point()+facet_wrap(~Cruise, scales='fixed', nrow=1)+geom_smooth(method='lm')+theme_classic(
>
> grid.arrange(g1,g2,nrow=2)
```

```
> g1 <- ggplot(kim, aes(y=ETS, x=Temp, color=Mesh)) + geom_point()+facet_wrap(~Cruise, scales='fixed',nrow=1)+geom_smooth(method='lm')+theme_classic()
> g2 <- ggplot(kim, aes(y=AARS, x=Temp, color=Mesh)) + geom_point()+facet_wrap(~Cruise, scales='fixed', nrow=1)+geom_smooth(method='lm')+theme_classic(
>
> grid.arrange(g1,g2,nrow=2)
```

```
> ggplot(csc, aes(y=ETS, x=Chl, color=Mesh)) + geom_point()+facet_wrap(~Cruise, scales='fixed')+geom_smooth(method='lm')+theme_classic()
```

```
> ggplot(csc, aes(y=AARS, x=Chl, color=Mesh)) + geom_point()+facet_wrap(~Cruise, scales='fixed')+geom_smooth(method='lm')+theme_classic()
```

```
> ggplot(kim, aes(y=ETS, x=Chl, color=Mesh)) + geom_point()+facet_wrap(~Cruise, scales='fixed')+geom_smooth(method='lm')+theme_classic()
```

```
> ggplot(kim, aes(y=AARS, x=Chl, color=Mesh)) + geom_point()+facet_wrap(~Cruise, scales='fixed')+geom_smooth(method='lm')+theme_classic()
```

```
> ggplot(csc, aes(y=ETS, x=Chl, color=Mesh)) + geom_point()+facet_wrap(~Cruise, scales='fixed')+geom_smooth(method='lm')+theme_classic()
```

```
> ggplot(csc, aes(y=AARS, x=Chl, color=Mesh)) + geom_point()+facet_wrap(~Cruise, scales='fixed')+geom_smooth(method='lm')+theme_classic()
```

```
> ggplot(kim, aes(y=ETS, x=Chl, color=Mesh)) + geom_point()+facet_wrap(~Cruise, scales='fixed')+geom_smooth(method='lm')+theme_classic()
```

```
> ggplot(kim, aes(y=AARS, x=Chl, color=Mesh)) + geom_point()+facet_wrap(~Cruise, scales='fixed')+geom_smooth(method='lm')+theme_classic()
```

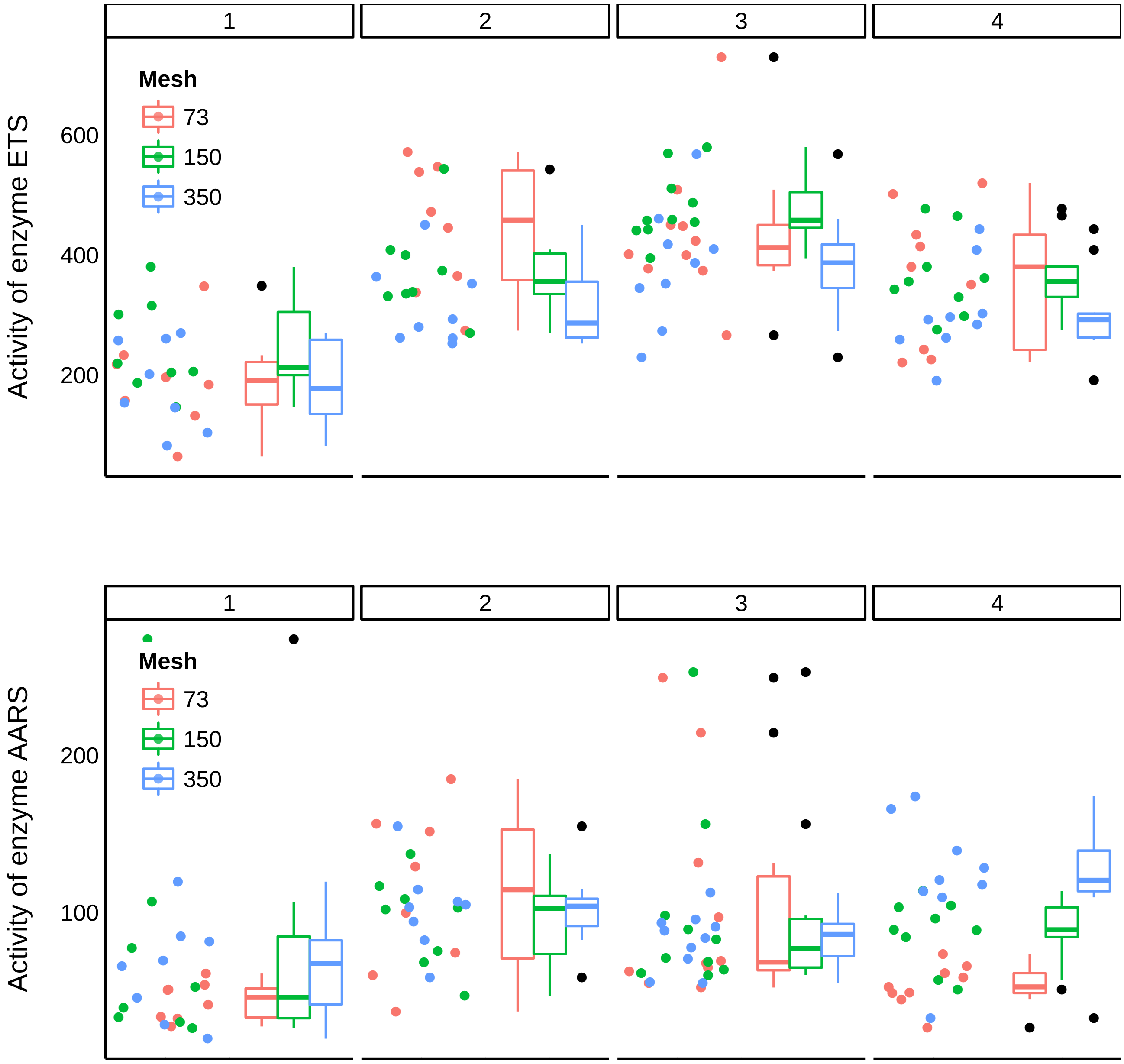

Figure A. plot of chunk eda

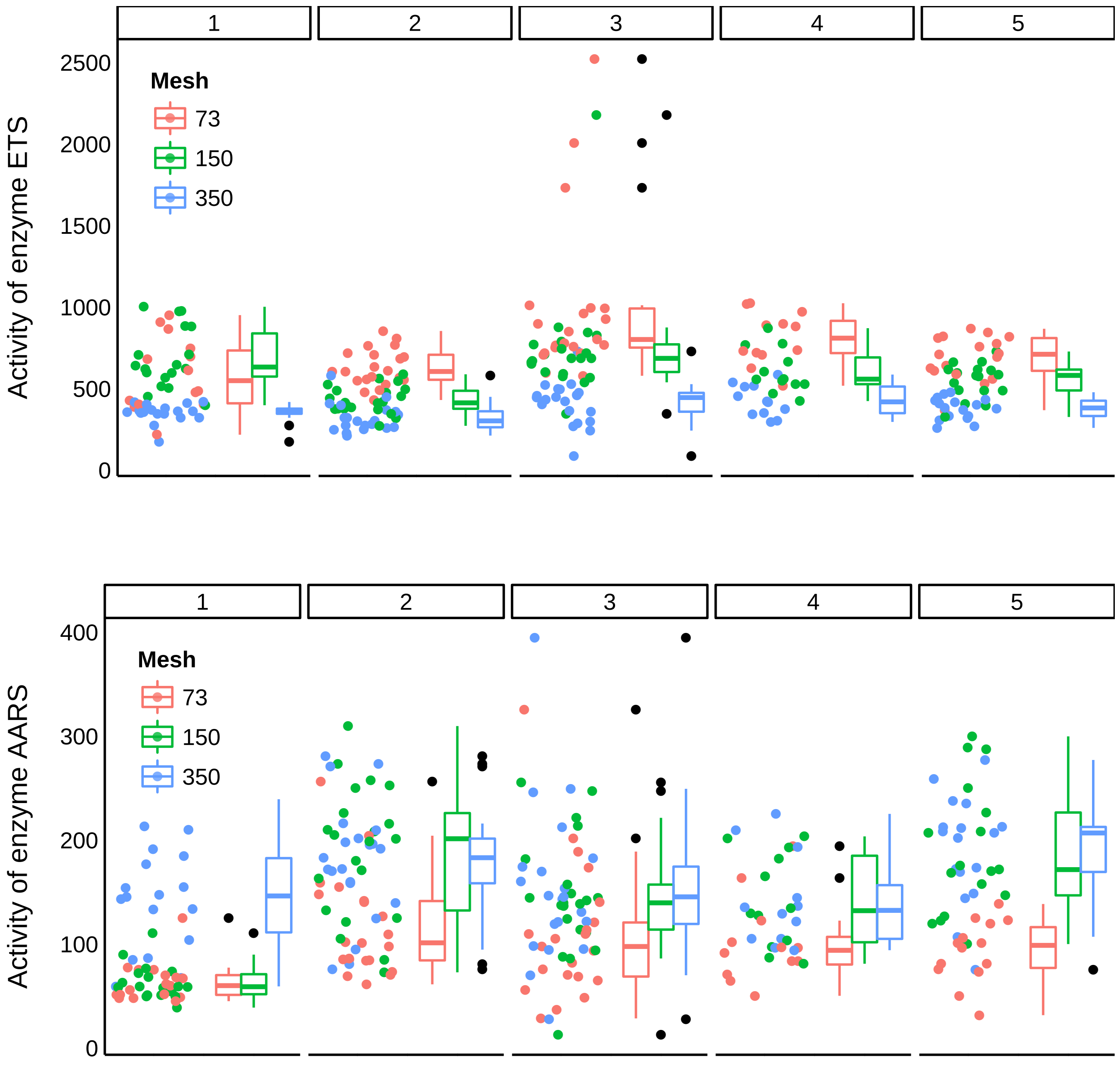

Figure B. plot of chunk eda

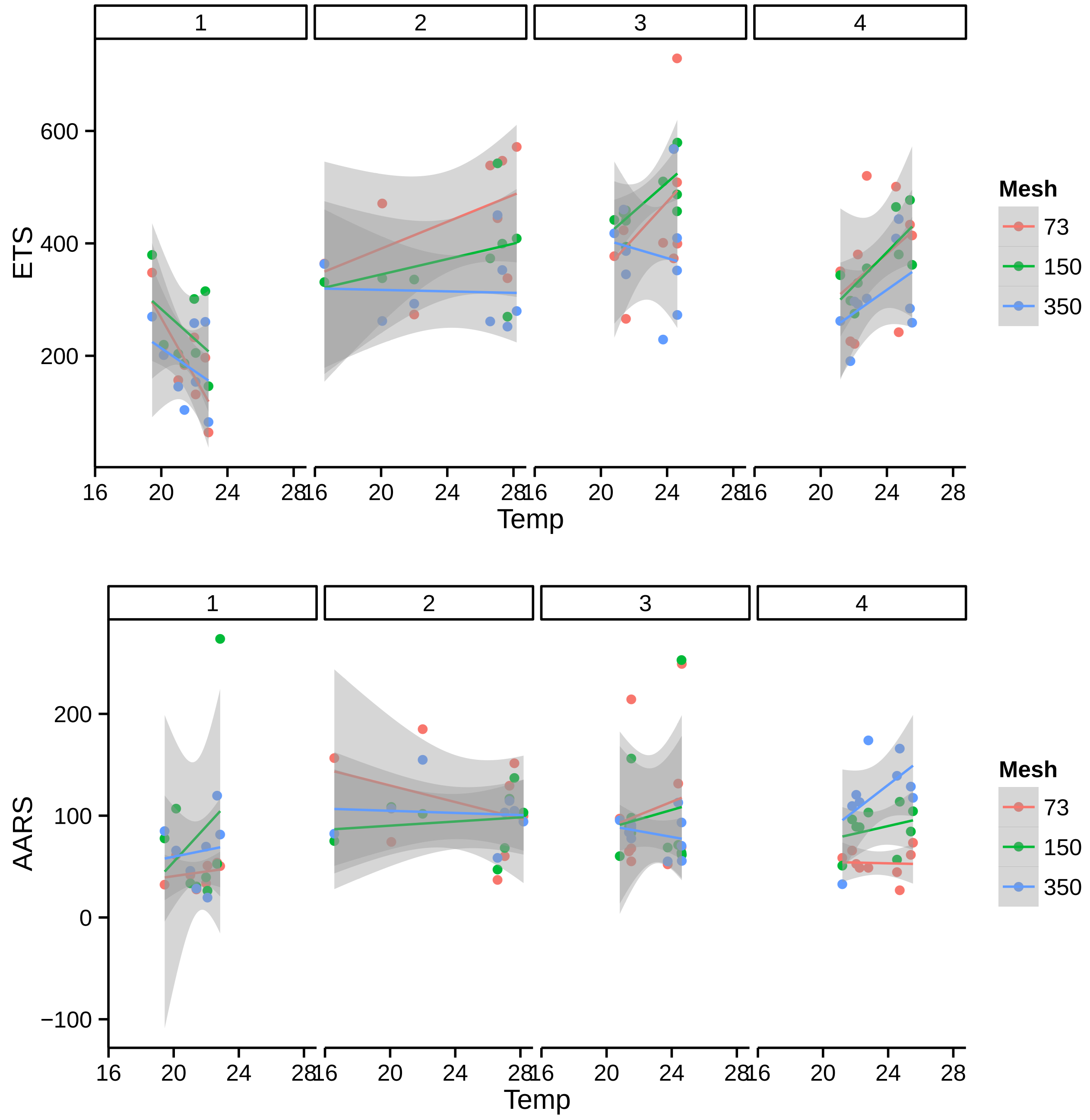

Figure C. plot of chunk eda1

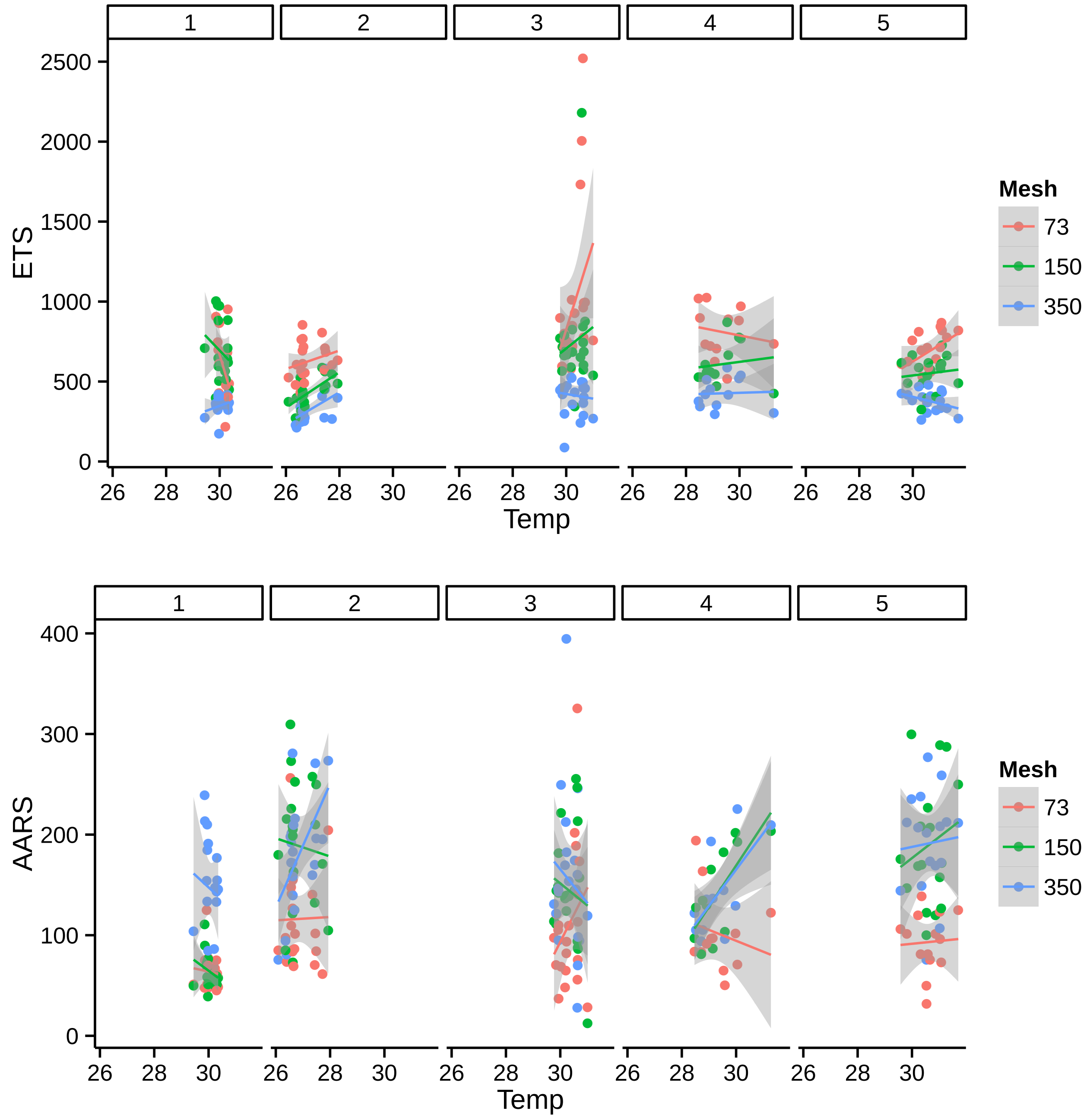

Figure D. plot of chunk eda1

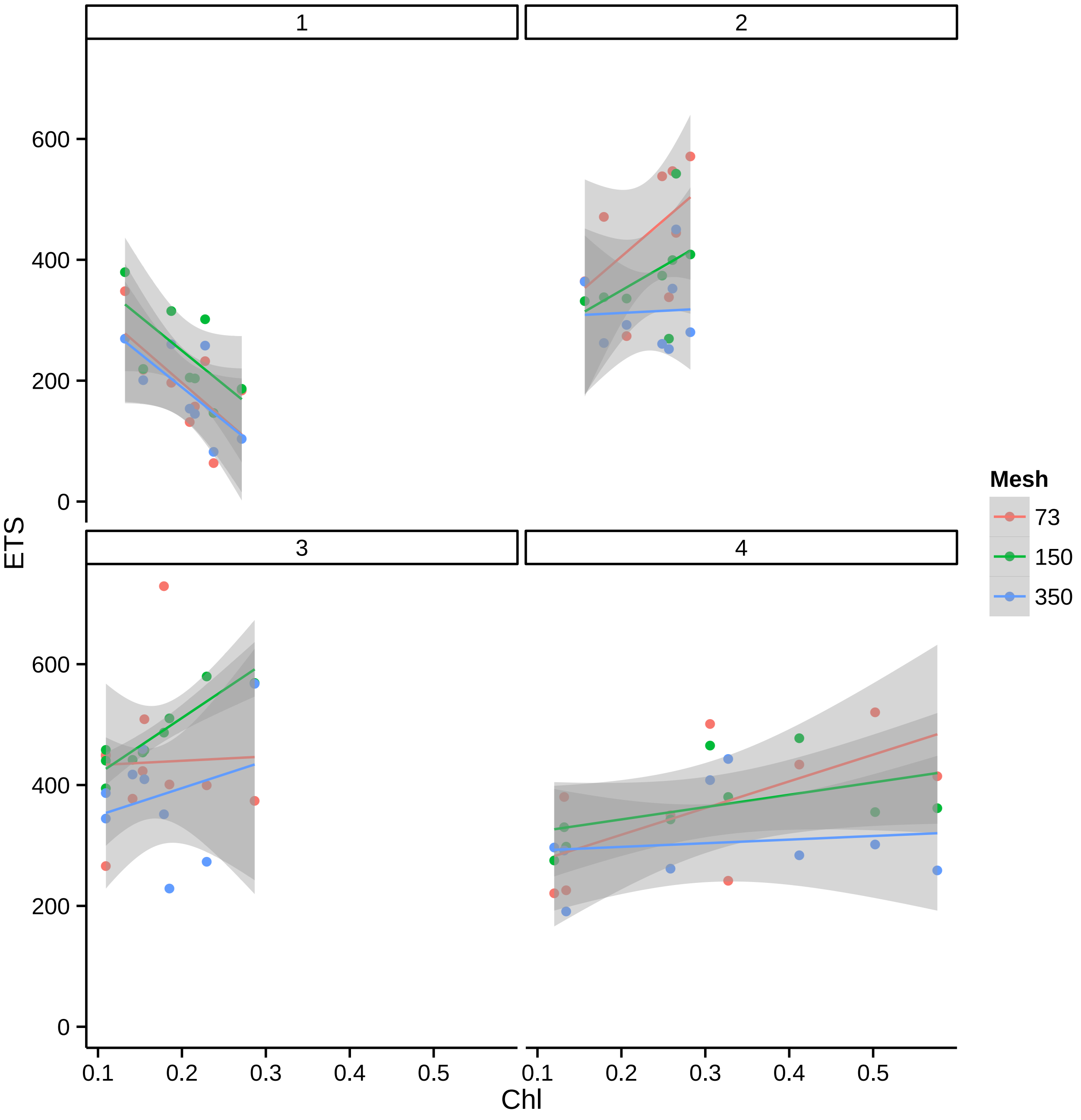

Figure E. plot of chunk eda2

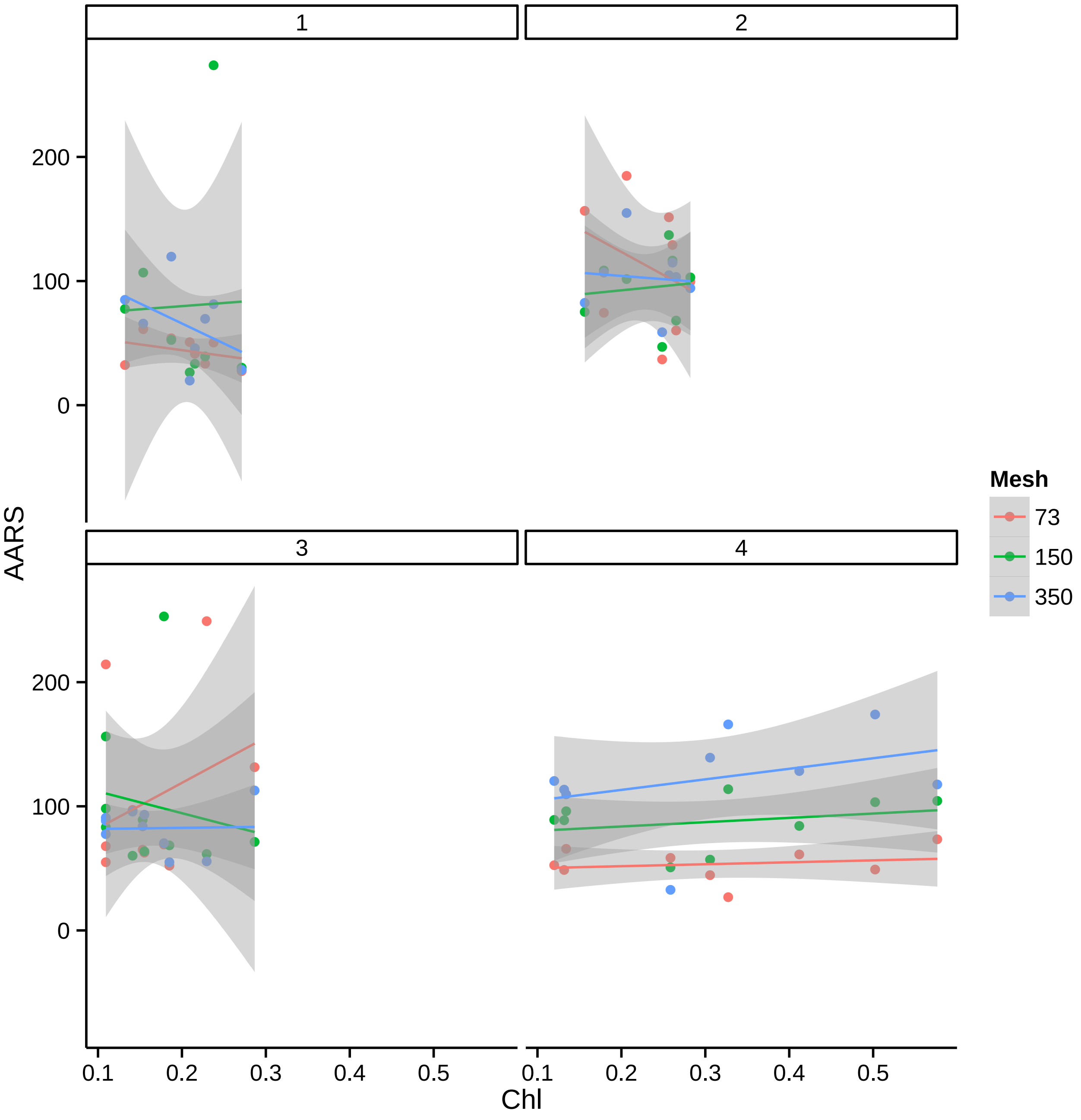

Figure F. plot of chunk eda2

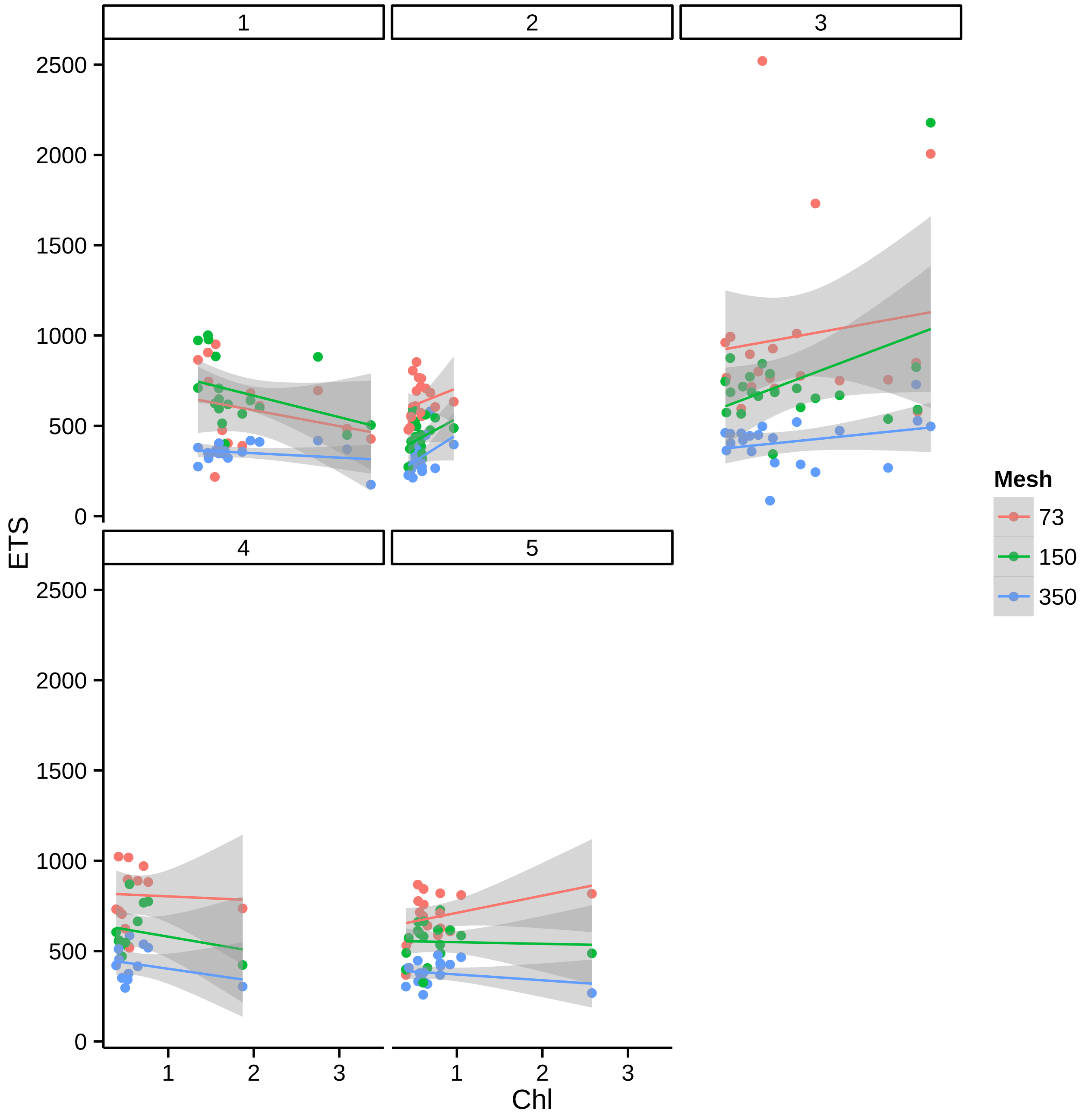

Figure G. plot of chunk eda2

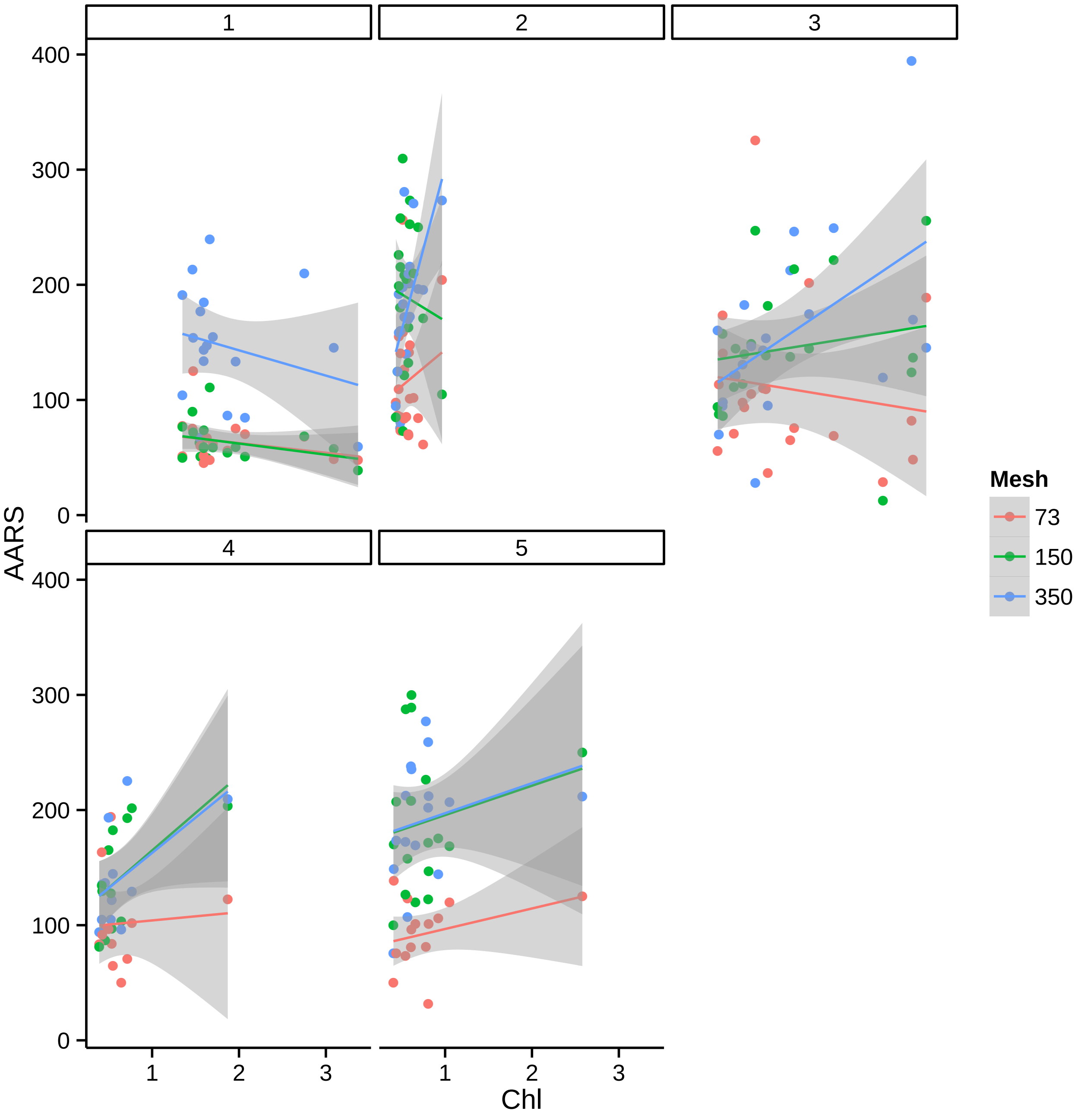

Figure H. plot of chunk eda2

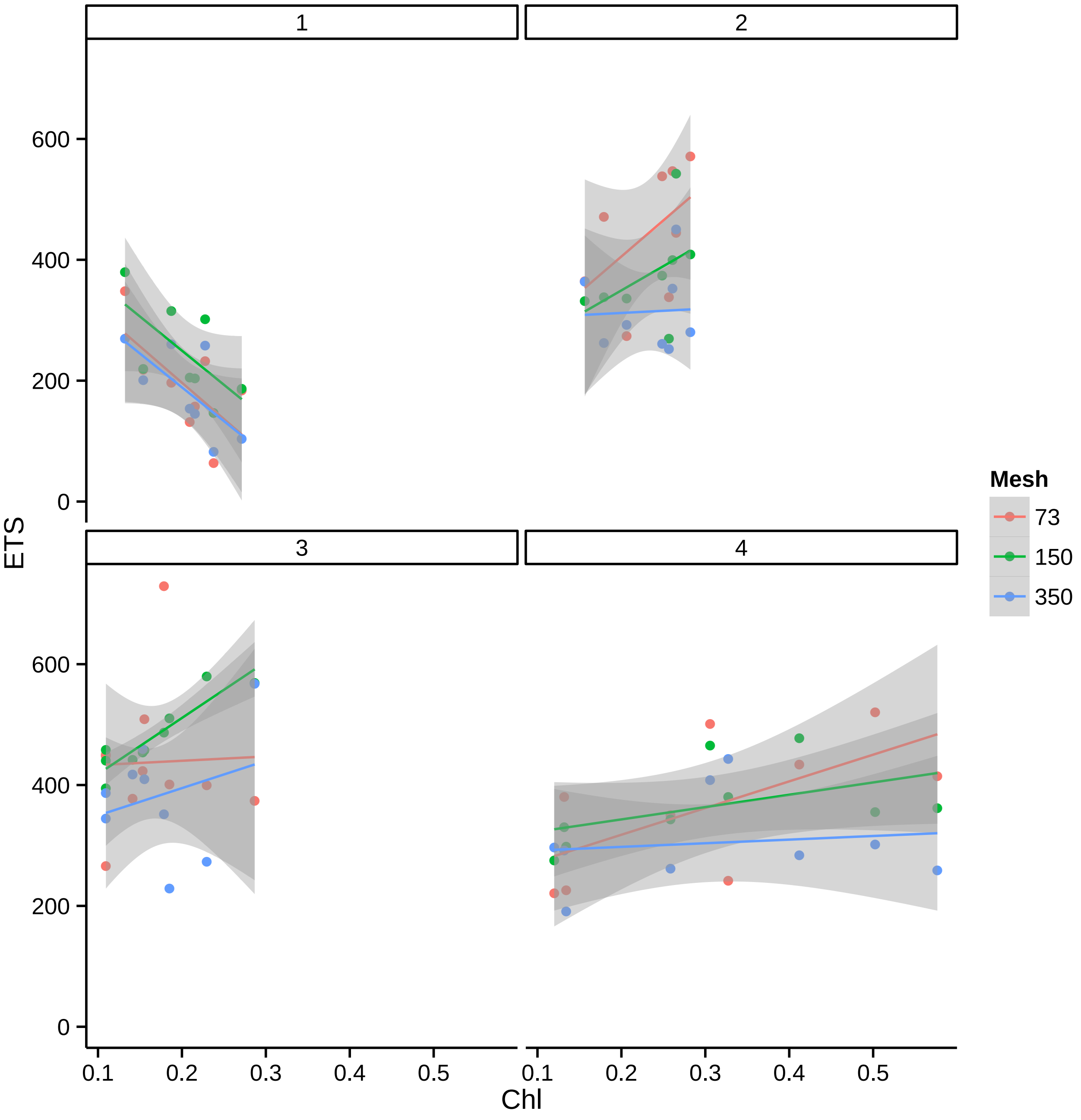

Figure I. plot of chunk eda3

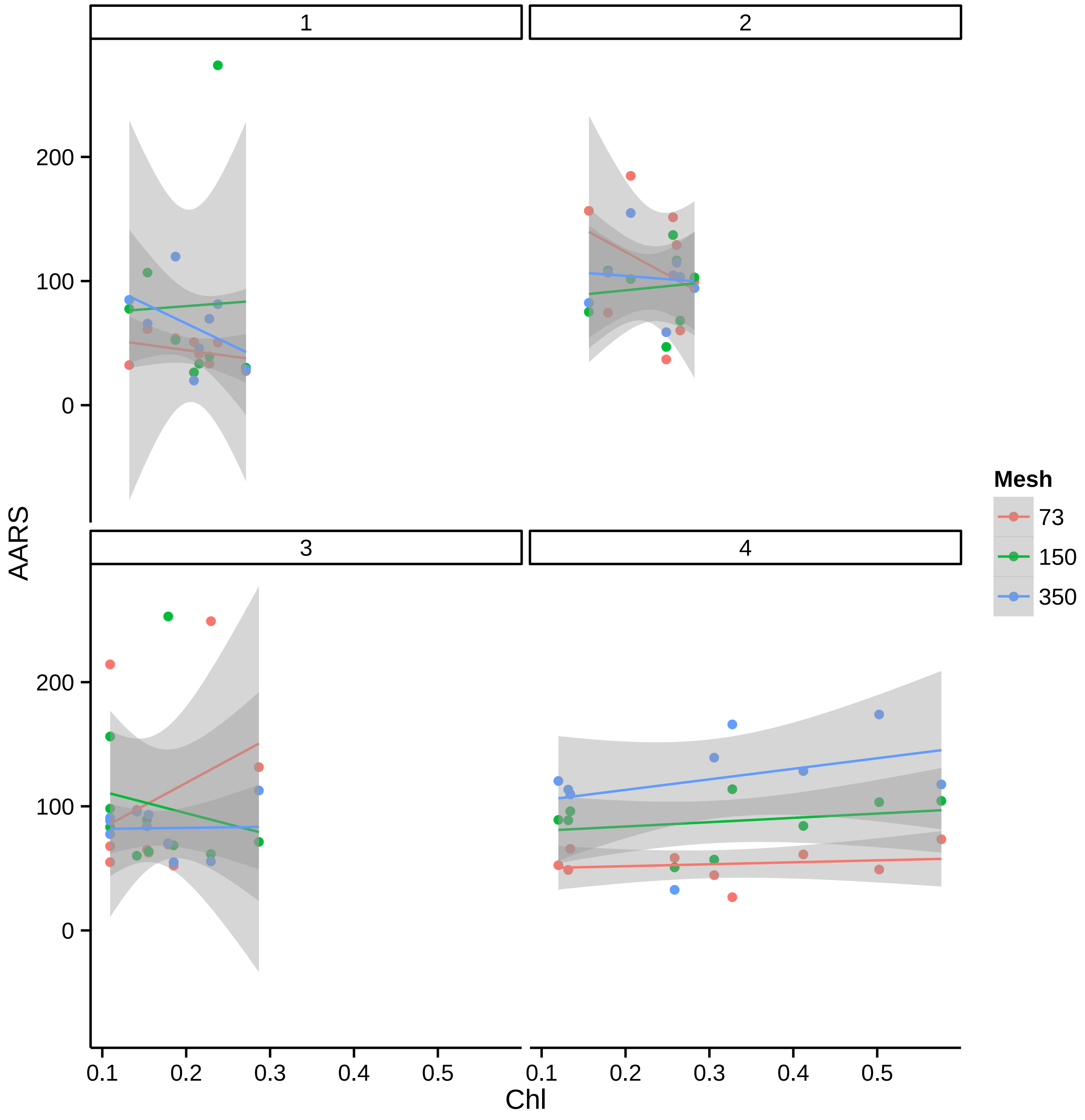

Figure J. plot of chunk eda3

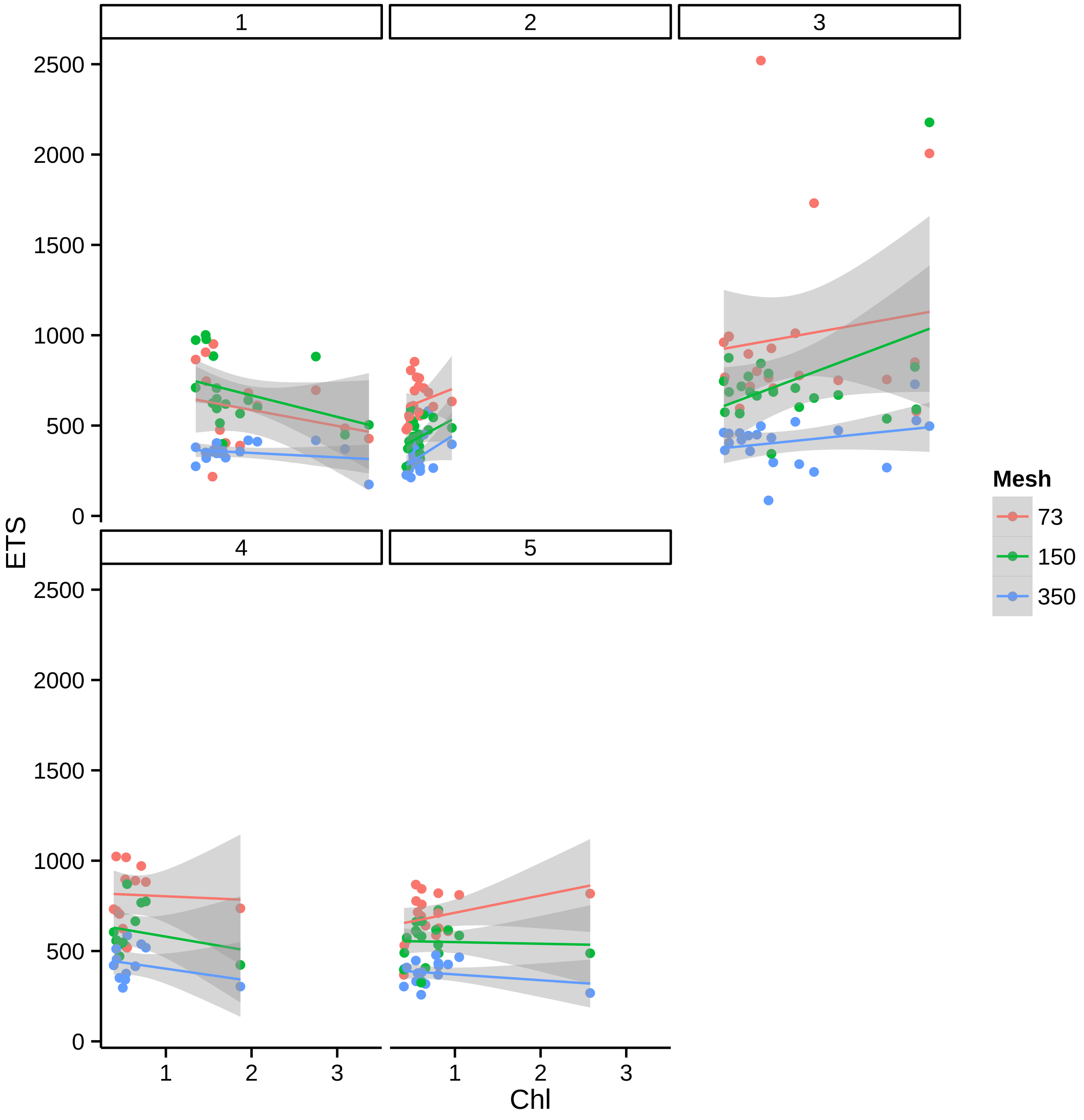

Figure K. plot of chunk eda3

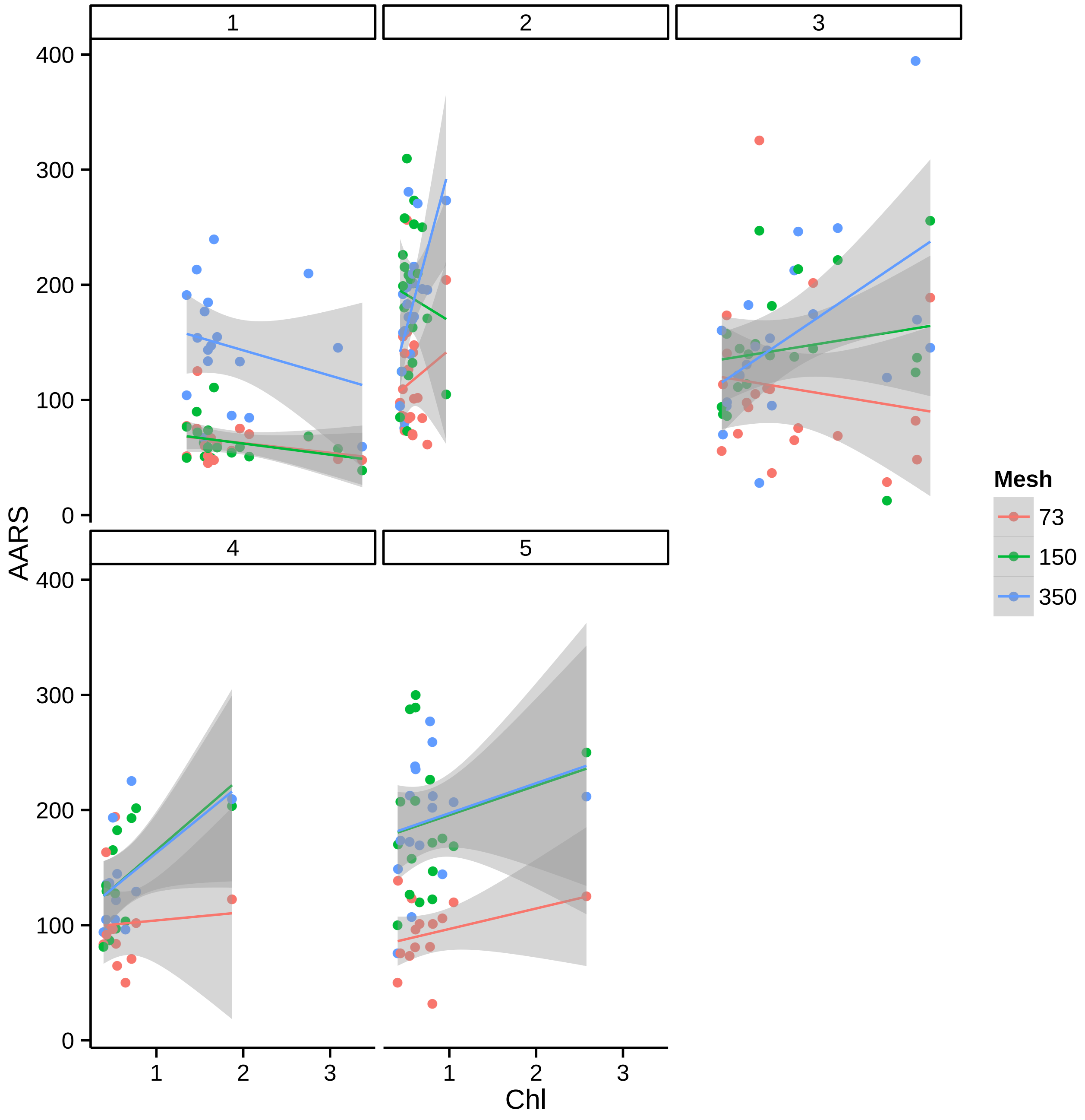

Figure L. plot of chunk eda3

So what if we partial out protein, chl and temperature... If we want to estimate the effects of protein, chlorophyll and temperature, we first need to make sure that they are not correlated to one another (multi-collinearity). However, if we just wish to partial them out, then it is not so much of an issue.

```
> library(GGally)
> dat <- within(subset(csc, select=c(ETS,AARS,Protein,Chl,Temp)),{
+     logAARS=log(AARS)
+     logProtein=log(Protein)
+     logChl=log(Chl)
+ })
> head(dat)
```

```
        ETS     AARS    Protein       Chl     Temp    logChl logProtein  logAARS
1  63.79607 50.53627 0.17469550 0.2378516 22.85509 -1.436109  -1.744711 3.922691
2 156.96130 41.39708 0.28281610 0.2154798 21.02649 -1.534888  -1.262958 3.723210
3 183.69280 27.46457 0.08221237 0.2713337 21.40192 -1.304406  -2.498450 3.312897
4 232.62470 33.56256 0.25477210 0.2276602 21.99414 -1.479901  -1.367386 3.513411
5 217.68900 61.14242 0.05529095 0.1539380 20.15350 -1.871205  -2.895146 4.113206
6 348.08290 32.42767 0.09302974 0.1321699 19.44771 -2.023667  -2.374836 3.479012
```

```
> p <- ggpairs(dat,columns=c(c(1,8,5:7)),
+     lower=list(continuous="smooth", params=c(colour="blue")),
+     diag=list(continuous="density",params=c(colour="blue")),
+     upper=list(params=list(corSize=6)), axisLabels='show')
> print(p)
```

I very much doubt that adding Temperature, Chlorophyll and Protein are going to do much...

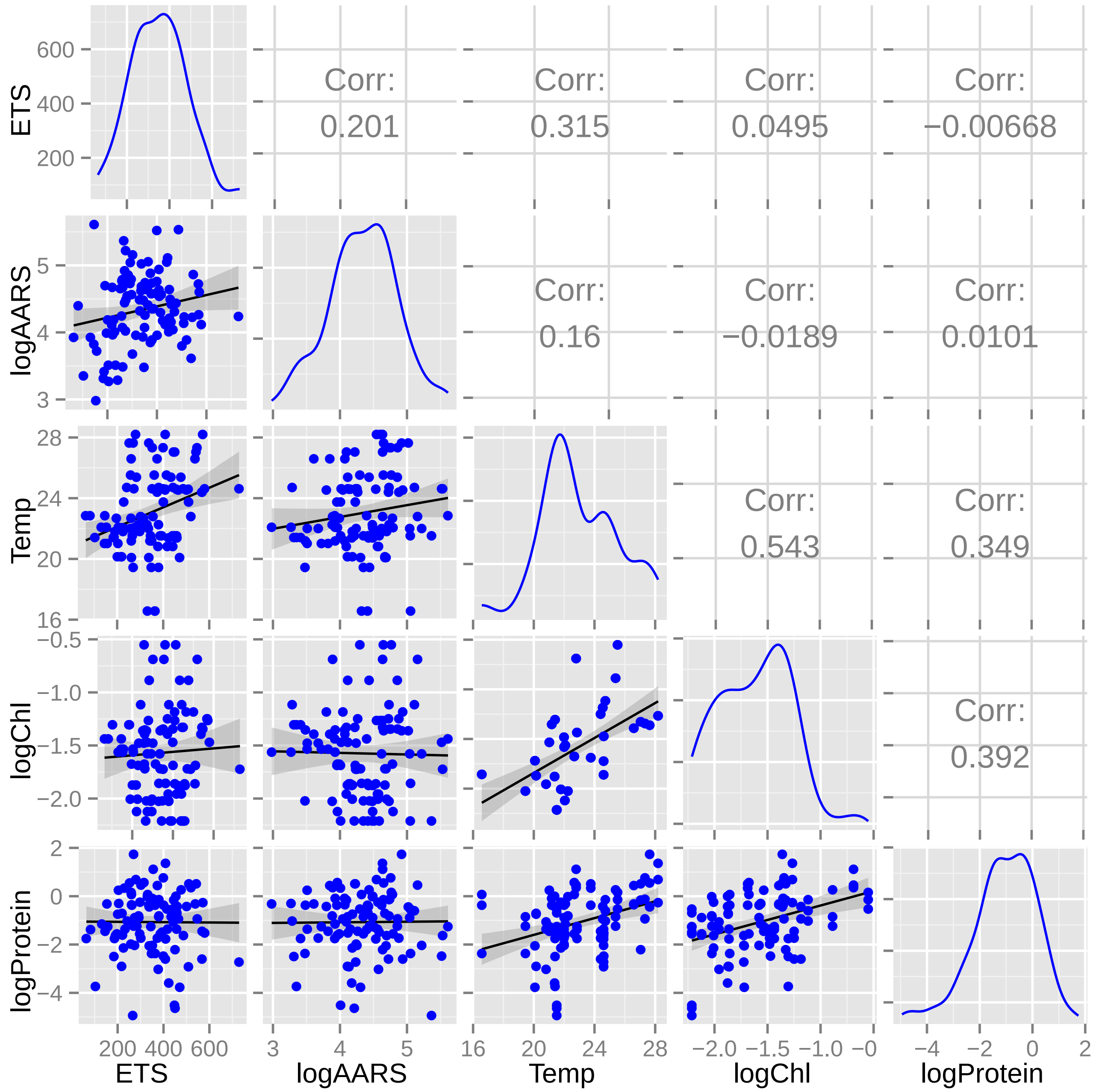

Figure M. plot of chunk eda4

3. Analyses

For each location, the effects of mesh size, cruise (and their interactions) on ETS and AARS were explored via linear models in a Bayesian framework using JAGS (Plummer 2003) interfaced through R (R Core Team 2014) (Team 2014) and the R2jags (Su and Yajima 2014) package. Models also included the covariates of protein (log transformed), chlorophyll (log transformed) and Temperature in an attempt to reduce residual uncertainty.

$$
\begin{aligned}
y_i &\sim \mathcal{N}(\mu_i, \sigma^2)\\
\mu_i &= \beta_0 + \beta X_i + \gamma W_i\\
X_{ijk} &= mesh_{ij} + cruise_{ik} + mesh : cruise_{ijk}\\
W_i &= log(protein_i) + log(chlorophyll_i) + temperature_i\\
\beta_0 &\sim \mathcal{N}(0, 100000)\\
\beta &\sim \mathcal{MVN}(0, 1000000)\\
\gamma &\sim \mathcal{MVN}(0, 1000000)\\
\sigma^2 &\sim \mathcal{HC}(25)
\end{aligned}
$$

where y_i are the i_{th} ETS or AARS observation from the j_{th} mesh and k_{th} cruise. Non-informative normal priors were specified for the intercept (β_0) and non-informative multivariate normal priors were specified for both the main effect (β) and covariate (γ) parameters. Half-cauchy (scale=25) priors where specified for the variance following (Gelman 2006).

A total of 10,000 Gibbs sampling iterations were performed across 3 chains with a burnin of 1,000 and thinning rate of 10, resulting in a total of 2,700 collected samples. Chain mixing and convergence were assessed via traceplots, autocorrelation and Gelman-Rubin diagnostics (all scale reduction factors less than 1.005)

The relative influences of the main effects of mesh, cruise and their interactions as well as the covariates (protein, chlorophyll and temperature) were assessed via finite-population standard deviations (Gelman and Hill 2007). Pairwise comparisons of mesh within each cruise and vice verse were derived from specific contrasts on the posteriors and all inferences about specific differences (effects) were based on 95% Bayesian UIs for modeled higher posterior density (HPD) median effects.

3.1. GBR

3.1.1. ETS

```
> model="
+ model {
+   for (i in 1:n) {
+     y[i] ~ dnorm(mu[i], tau)
+     mu[i] <- inprod(beta[],X[i,])+inprod(gamma[],XX[i,])
+     y.err[i] <- y[i] - mu[i]
+   }
+   beta ~ dmnorm(a0,A0)
+   gamma ~ dmnorm(c0,C0)
+   tau <- pow(sigma,-2)
+   sigma <- z/sqrt(chSq)
+   z ~ dnorm(0, 0.0016)I(0,)  #1/25^2 = 0.0016
+   chSq ~ dgamma(0.5, 0.5)
+   sd.res <- sd(y.err[])
+   b[1] <- 0
+   b[2:3] <- beta[2:3]
+   sd.mesh <- sd(b)
+   c[1] <-0
+   c[2:4] <- beta[4:6]
+   sd.cruise <- sd(c)
+   for (i in 1:6) {d[i] <-0}
+   d[7:12] <- beta[7:12]
+   sd.int <- sd(d)
+   sd.protein <- abs(gamma[1])*sd(XX[,1])
+   sd.chl <- abs(gamma[2])*sd(XX[,2])
+   sd.temp <- abs(gamma[3])*sd(XX[,3])
+ }
+ "
>
> contrasts(csc$Mesh) <- contr.treatment
> contrasts(csc$Cruise) <- contr.treatment
> dat1 <- subset(csc, !is.na(Protein))
> X <- model.matrix(~Mesh*Cruise, data=dat1)
> nX <- ncol(X)
> XX <- model.matrix(~log(Protein)+log(Chl)+Temp, dat1)[,-1]
> nXX <- ncol(XX)
> csc.list <- with(dat1, list(y=ETS,
+                            n=nrow(dat1),
+                            X=X,nX=nX,
+                            XX=XX,
+                            a0=rep(0,nX), A0=diag(1.0E-06,nX),
+                            c0=rep(0,nXX), C0=diag(1.0E-06,nXX)))
> param <- c('beta','gamma','sigma', 'sd.res', 'sd.mesh','sd.cruise','sd.int','sd.protein','sd.chl','sd.temp')
> csc.jags <- jags(data=csc.list,init=NULL, parameters.to.save=param,
+                  model.file=textConnection(model),
+                  n.chains=3,
+                  n.iter=10000,
+                  n.burnin=1000,
+                  n.thin=10)
```

```
Compiling model graph
   Resolving undeclared variables
   Allocating nodes
   Graph Size: 2394

Initializing model
```

```
> csc.jags
```

```
Inference for Bugs model at "5", fit using jags,
 3 chains, each with 10000 iterations (first 1000 discarded), n.thin = 10
 n.sims = 2700 iterations saved
            mu.vect sd.vect     2.5%      25%      50%      75%    97.5%  Rhat n.eff
beta[1]     158.783 137.113 -118.662   67.214  159.437  251.049  424.079 1.001  2700
beta[2]      58.188  44.008  -28.529   28.570   58.229   86.950  143.654 1.000  2700
beta[3]      -5.380  43.121  -88.330  -34.326   -4.895   22.548   78.835 1.003   960
beta[4]     233.231  44.184  144.548  204.462  233.533  262.363  319.241 1.001  2700
beta[5]     242.935  44.188  152.640  214.401  244.027  271.950  326.218 1.001  2700
beta[6]     157.038  43.260   73.160  128.124  157.341  186.318  242.360 1.001  2700
beta[7]    -118.560  62.134 -240.526 -158.773 -119.914  -77.973    3.581 1.001  2700
```

```
beta[8]    -115.124  61.766 -240.722 -154.989 -114.449  -74.153    7.164 1.003   830
beta[9]      -5.937  58.164 -116.429  -45.306   -6.362   31.754  112.770 1.001  2700
beta[10]    -27.268  60.131 -146.750  -67.034  -27.472   12.506   88.952 1.001  2700
beta[11]    -55.337  58.709 -166.947  -94.026  -55.598  -18.295   63.623 1.001  2700
beta[12]    -56.102  59.638 -175.323  -94.756  -57.434  -16.243   64.524 1.003   890
gamma[1]     -4.753   9.929  -24.709  -11.250   -4.836    1.684   15.571 1.001  2000
gamma[2]     45.630  29.718  -12.108   26.161   46.101   64.846  106.523 1.001  2700
gamma[3]      4.584   4.627   -4.490    1.504    4.532    7.662   13.947 1.001  2700
sd.chl       19.673  11.217    1.089   11.182   19.209   26.968   44.301 1.001  2700
sd.cruise   115.224  17.740   78.823  103.786  115.393  126.964  149.785 1.001  2700
sd.int       56.941  16.726   27.419   45.037   55.745   67.361   92.643 1.003   780
sd.mesh      42.768  19.239    9.764   28.557   41.963   55.475   82.142 1.001  2700
sd.protein   11.159   8.411    0.430    4.536    9.366   15.889   32.048 1.001  2300
sd.res       86.621   2.522   82.752   84.764   86.278   88.088   92.381 1.001  2700
sd.temp      13.644   9.457    0.456    6.149   12.142   19.627   35.663 1.001  2700
sigma        86.971   6.716   75.624   82.321   86.454   90.961  101.815 1.001  2700
deviance   1200.645   6.071 1190.937 1196.186 1199.899 1204.101 1214.317 1.001  2700

For each parameter, n.eff is a crude measure of effective sample size,
and Rhat is the potential scale reduction factor (at convergence, Rhat=1).

DIC info (using the rule, pD = var(deviance)/2)
pD = 18.4 and DIC = 1219.1
DIC is an estimate of expected predictive error (lower deviance is better).
```

```
> ## Finite-population standard deviations
> X.mesh.var <- csc.jags$BUGSoutput$sims.list[['sd.mesh']]^2
> X.protein.var <- csc.jags$BUGSoutput$sims.list[['sd.protein']]^2
> X.chl.var <- csc.jags$BUGSoutput$sims.list[['sd.chl']]^2
> X.temp.var <- csc.jags$BUGSoutput$sims.list[['sd.temp']]^2
> X.cruise.var <- csc.jags$BUGSoutput$sims.list[['sd.cruise']]^2
> X.int.var <- csc.jags$BUGSoutput$sims.list[['sd.int']]^2
> R.var <- csc.jags$BUGSoutput$sims.list[['sd.res']]^2
>
> R2.X<-(X.mesh.var + X.cruise.var + X.int.var + X.protein.var + X.chl.var + X.temp.var)/(X.mesh.var + X.cruise.var + X.int.var + X.protein.var + X.chl
> R2.mesh<-((X.mesh.var)/(X.mesh.var + X.cruise.var + X.int.var + X.protein.var + R.var))
> R2.cruise<-((X.cruise.var)/(X.mesh.var + X.cruise.var + X.int.var + X.protein.var + X.chl.var + X.temp.var + R.var))
> R2.int<-((X.int.var)/(X.mesh.var + X.cruise.var + X.int.var + X.protein.var + X.chl.var + X.temp.var + R.var))
> R2.protein<-((X.protein.var)/(X.mesh.var + X.cruise.var + X.int.var + X.protein.var + X.chl.var + X.temp.var + R.var))
> R2.chl<-((X.chl.var)/(X.mesh.var + X.cruise.var + X.int.var + X.chl.var + X.chl.var + X.temp.var + R.var))
> R2.temp<-((X.temp.var)/(X.mesh.var + X.cruise.var + X.int.var + X.temp.var + X.chl.var + X.temp.var + R.var))
> R2.resid <- ((R.var)/(X.mesh.var + X.cruise.var + X.int.var + X.protein.var + X.chl.var + X.temp.var + R.var))
>
> a<-cbind(Residual=R.var, 'Temperature'=X.temp.var, 'Chlorophyll'=X.chl.var, 'Protein'=X.protein.var,'Mesh x Cruise'=X.int.var, Cruise=X.cruise.var, M
> sdtab <- cbind(Name=c('Residuals','Temperature','Chlorophyll','Protein','Mesh x Cruise','Cruise','Mesh'),adply(a, 2, function(x) {
+     data.frame(Mean=mean(x), Median=median(x), HPDinterval(as.mcmc(x)), HPDinterval(as.mcmc(x), p=0.68))
+     }))
>
> a<-cbind(Residual=R2.resid, 'Temperature'=R2.temp, 'Chlorophyll'=R2.chl, 'Protein'=R2.protein, 'Mesh x Cruise'=R2.int, Cruise=R2.cruise, Mesh=R2.mesh
> R2tab <- cbind(Name=c('Residuals','Temperature','Chlorophyll','Protein','Mesh x Cruise','Cruise','Mesh'),adply(a, 2, function(x) {
+     data.frame(Mean=mean(x), Median=median(x), HPDinterval(as.mcmc(x)), HPDinterval(as.mcmc(x), p=0.68))
+     }))
> sdtab$R2 <- round(R2tab$Median,3)
>
>
> sdtab$Name <- factor(sdtab$Name, levels=c('Residuals','Temperature','Chlorophyll','Protein','Mesh x Cruise','Cruise','Mesh'))
> sdtab <- subset(sdtab, select=-X1)
>
> print(xtable(sdtab[7:1,], caption='Mean, median, (lower and upper) and highest probability density intervals of finite-population standard deviations
```

% latex table generated in R 3.1.0 by xtable 1.7-4 package % Thu May 7 08:35:39 2015

Table S1. Mean, median, (lower and upper) and highest probability density intervals of finite-population standard deviations as well as marginal R^2 approximations.

Name	Mean	Median	lower	upper	lower.1	upper.1	R2
Mesh	2199.09	1760.93	4.54	5814.05	0.77	2634.47	0.07
Cruise	13591.25	13315.60	5649.63	21550.91	9200.58	17120.02	0.49
Mesh x Cruise	3521.91	3107.55	365.43	7662.14	1145.01	4541.36	0.12
Protein	195.24	87.71	0.00	743.45	0.00	189.28	0.00
Chlorophyll	512.82	368.99	0.00	1578.56	0.00	612.80	0.01
Temperature	275.56	147.42	0.00	984.12	0.00	300.94	0.01
Residuals	7509.60	7443.88	6813.96	8437.30	6961.77	7760.70	0.28

```
> p1 <- ggplot(sdtab, aes(y=Name,x=Median)) +
+     geom_vline(xintercept=0,linetype='dashed')+
+         geom_errorbarh(aes(xmin=lower, xmax=upper), height=0)+
+                     geom_errorbarh(aes(xmin=lower.1, xmax=upper.1), height=0, size=2)+
+         geom_point(size=3, shape=21, fill='white')+
+             geom_text(aes(label=R2, y=as.numeric(Name)+0.3),position=position_dodge(height=1), size=3)+
+             scale_x_continuous('Finite-population standard deviation')+
+             theme_classic(8)+theme(axis.title.y=element_blank(),axis.title.x=element_text(vjust=-2, size=rel(1.25)),
+                                   plot.margin=unit(c(0,0,2,2), 'lines'))
> print(p1)
```

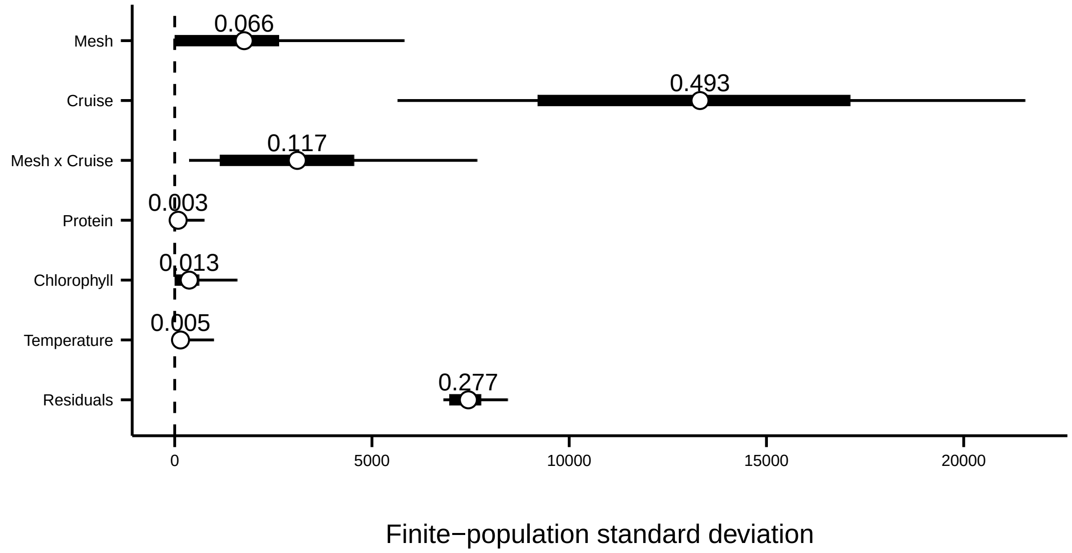

Figure N. Median (lower and upper) highest probability density intervals of finite-population standard deviations. R^2 approximations above medians.

```
> newdata <- expand.grid(Mesh=levels(csc$Mesh), Cruise=levels(csc$Cruise))
> newdata$Protein <- mean(csc$Protein, na.rm=TRUE)
> newdata$Chl <- mean(csc$Chl, na.rm=TRUE)
> newdata$Temp <- mean(csc$Temp, na.rm=TRUE)
> Xmat <- model.matrix(~Mesh*Cruise, data=newdata)
> XXmat <- model.matrix(~log(Protein)+log(Chl)+Temp, data=newdata)[,-1]
> coef <- csc.jags$BUGSoutput$sims.list[['beta']]
> fit <- coef %*% t(Xmat) + csc.jags$BUGSoutput$sims.list[['gamma']] %*% t(XXmat)
> library(plyr)
> newdata<- cbind(newdata, adply(fit, 2, function(x) {
+     data.frame(Mean=mean(x), Median=median(x), HPDinterval(as.mcmc(x)),HPDinterval(as.mcmc(x), p=0.68))
+     }))
>
> #newdata
>
> #Comparisons
> MCMCsum <- function(x) {
+    data.frame(Median=median(x, na.rm=TRUE),
+               t(quantile(x,na.rm=TRUE)),
+               HPDinterval(as.mcmc(x)),
+               HPDinterval(as.mcmc(x), p=0.68))
+ }
> library(multcomp)
> ndata <- newdata
> ndata<- subset(ndata, Cruise=='1')
> Xmat <- contrMat(n=rep(1,nrow(ndata)), type="Tukey") %*% model.matrix(~Mesh*Cruise, ndata, xlev=list(Mesh=levels(csc$Mesh), Cruise=levels(csc$Cruise)
> wch <- ldply(strsplit(rownames(Xmat)," - "),as.numeric)
> rownames(Xmat) <- paste(interaction(newdata$Mesh, newdata$Cruise)[wch[,1]],"-",interaction(newdata$Mesh,newdata$Cruise)[wch[,2]])
>  pairwise.comps <- coef %*% t(Xmat)
> comp.mesh_Cruise1<-adply(pairwise.comps,2, MCMCsum)
>
> ndata <- newdata
> ndata<- subset(ndata, Cruise=='2')
> Xmat <- contrMat(n=rep(1,nrow(ndata)), type="Tukey") %*% model.matrix(~Mesh*Cruise, ndata, xlev=list(Mesh=levels(csc$Mesh), Cruise=levels(csc$Cruise)
> wch <- ldply(strsplit(rownames(Xmat)," - "),as.numeric)
> rownames(Xmat) <- paste(interaction(newdata$Mesh, newdata$Cruise)[wch[,1]],"-",interaction(newdata$Mesh,newdata$Cruise)[wch[,2]])
>  pairwise.comps <- coef %*% t(Xmat)
> comp.mesh_Cruise2<-adply(pairwise.comps,2, MCMCsum)
>
> ndata <- newdata
> ndata<- subset(ndata, Cruise=='3')
> Xmat <- contrMat(n=rep(1,nrow(ndata)), type="Tukey") %*% model.matrix(~Mesh*Cruise, ndata, xlev=list(Mesh=levels(csc$Mesh), Cruise=levels(csc$Cruise)
> wch <- ldply(strsplit(rownames(Xmat)," - "),as.numeric)
> rownames(Xmat) <- paste(interaction(newdata$Mesh, newdata$Cruise)[wch[,1]],"-",interaction(newdata$Mesh,newdata$Cruise)[wch[,2]])
>  pairwise.comps <- coef %*% t(Xmat)
> comp.mesh_Cruise3<-adply(pairwise.comps,2, MCMCsum)
>
> ndata <- newdata
> ndata<- subset(ndata, Cruise=='4')
> Xmat <- contrMat(n=rep(1,nrow(ndata)), type="Tukey") %*% model.matrix(~Mesh*Cruise, ndata, xlev=list(Mesh=levels(csc$Mesh), Cruise=levels(csc$Cruise)
> wch <- ldply(strsplit(rownames(Xmat)," - "),as.numeric)
> rownames(Xmat) <- paste(interaction(newdata$Mesh, newdata$Cruise)[wch[,1]],"-",interaction(newdata$Mesh,newdata$Cruise)[wch[,2]])
>  pairwise.comps <- coef %*% t(Xmat)
> comp.mesh_Cruise4<-adply(pairwise.comps,2, MCMCsum)
>
> comp.mesh <- rbind(data.frame(Cruise=1,comp.mesh_Cruise1),
+                    data.frame(Cruise=2,comp.mesh_Cruise2),
+                    data.frame(Cruise=3,comp.mesh_Cruise3),
+                    data.frame(Cruise=4,comp.mesh_Cruise4))
>
> p1 <- ggplot(comp.mesh, aes(x=Cruise, y=Median, color=X1)) +
+         geom_hline(yintercept=0,linetype='dashed')+
+                     geom_errorbar(aes(ymin=lower, ymax=upper), width=0, position=position_dodge(width=0.5))+
+                     geom_errorbar(aes(ymin=lower.1, ymax=upper.1), width=0, size=2, position=position_dodge(width=0.5))+
+                         geom_point(size=3, shape=21, fill='white',position=position_dodge(width=0.5))+
+                             scale_y_continuous('Median effect size')+
+                                 #scale_x_discrete('Cruise number')+
+                                 scale_color_manual('Mesh contrasts', breaks=c('150.1 - 73.1', '350.1 - 73.1','350.1 - 150.1'), labels=c('150 vs 73','
+                             coord_flip()+theme_classic()+
+                                 theme(axis.title.y=element_text(size=rel(1.25), vjust=2),
+                                       axis.title.x=element_text(size=rel(1.25), vjust=-1),
+                                       plot.margin=unit(c(0,0,1,1), 'lines'))
>
>
>
> ndata <- newdata
> ndata<- subset(ndata, Mesh=='73')
> Xmat <- contrMat(n=rep(1,nrow(ndata)), type="Tukey") %*% model.matrix(~Mesh*Cruise, ndata, xlev=list(Mesh=levels(csc$Mesh), Cruise=levels(csc$Cruise)
>  pairwise.comps <- coef %*% t(Xmat)
> comp.cruise_Mesh73<-adply(pairwise.comps,2, MCMCsum)
>
> ndata <- newdata
> ndata<- subset(ndata,Mesh=='150')
> Xmat <- contrMat(n=rep(1,nrow(ndata)), type="Tukey") %*% model.matrix(~Mesh*Cruise, ndata, xlev=list(Mesh=levels(csc$Mesh), Cruise=levels(csc$Cruise)
>  pairwise.comps <- coef %*% t(Xmat)
> comp.cruise_Mesh150<-adply(pairwise.comps,2, MCMCsum)
>
> ndata <- newdata
> ndata<- subset(ndata, Mesh=='350')
> Xmat <- contrMat(n=rep(1,nrow(ndata)), type="Tukey") %*% model.matrix(~Mesh*Cruise, ndata, xlev=list(Mesh=levels(csc$Mesh), Cruise=levels(csc$Cruise)
>  pairwise.comps <- coef %*% t(Xmat)
> comp.cruise_Mesh350<-adply(pairwise.comps,2, MCMCsum)
>
> comp.cruise <- rbind(data.frame(Mesh=73,comp.cruise_Mesh73),
+                      data.frame(Mesh=150,comp.cruise_Mesh150),
+                      data.frame(Mesh=350,comp.cruise_Mesh350))
>
```

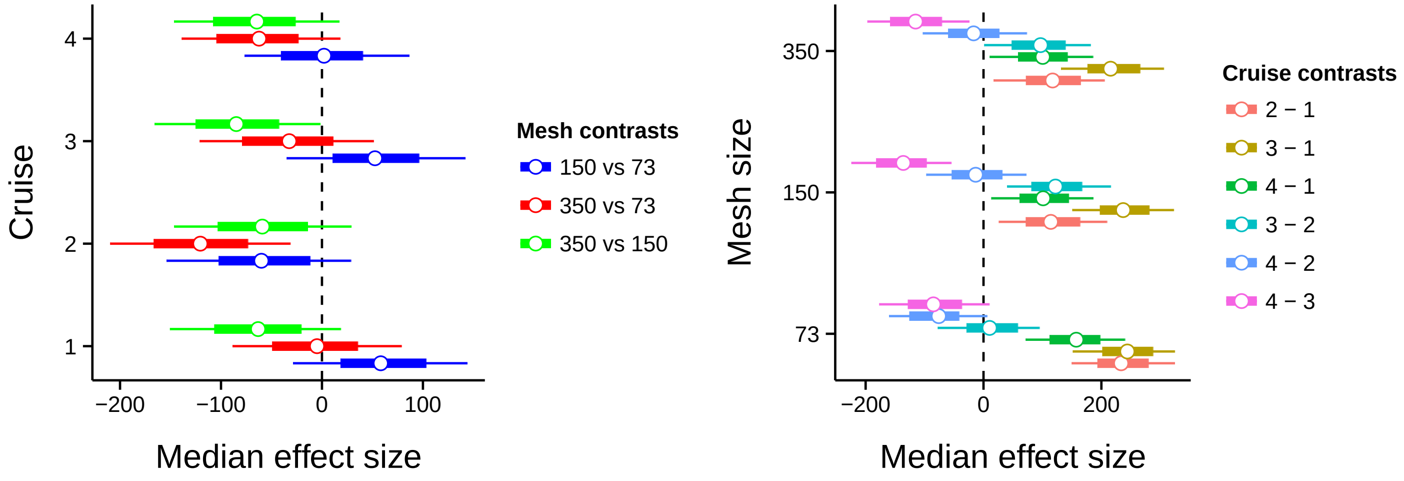

Figure O. plot of chunk ETScontrasts

3.1.2. AARS

```
> model="
+ model {
+   for (i in 1:n) {
+     y[i] ~ dnorm(mu[i], tau)
+     mu[i] <- inprod(beta[],X[i,])+inprod(gamma[],XX[i,])
+     y.err[i] <- y[i] - mu[i]
+   }
+   beta ~ dmnorm(a0,A0)
+   gamma ~ dmnorm(c0,C0)
+   tau <- pow(sigma,-2)
+   sigma <- z/sqrt(chSq)
+   z ~ dnorm(0, 0.0016)I(0,)  #1/25^2 = 0.0016
+   chSq ~ dgamma(0.5, 0.5)
+   sd.res <- sd(y.err[])
+   b[1] <- 0
+   b[2:3] <- beta[2:3]
+   sd.mesh <- sd(b)
+   c[1] <-0
+   c[2:4] <- beta[4:6]
+   sd.cruise <- sd(c)
+   for (i in 1:6) {d[i] <-0}
+   d[7:12] <- beta[7:12]
+   sd.int <- sd(d)
+   sd.protein <- abs(gamma[1])*sd(XX[,1])
+   sd.chl <- abs(gamma[2])*sd(XX[,2])
+   sd.temp <- abs(gamma[3])*sd(XX[,3])
+ }
+ "
>
> contrasts(csc$Mesh) <- contr.treatment
> contrasts(csc$Cruise) <- contr.treatment
> dat1 <- subset(csc, !is.na(Protein))
> X <- model.matrix(~Mesh*Cruise, data=dat1)
> nX <- ncol(X)
> XX <- model.matrix(~log(Protein)+log(Chl)+Temp, dat1)[,-1]
> nXX <- ncol(XX)
> csc.list <- with(dat1, list(y=AARS,
+                            n=nrow(dat1),
+                            X=X,nX=nX,
+                            XX=XX,
+                            a0=rep(0,nX), A0=diag(1.0E-06,nX),
+                            c0=rep(0,nXX), C0=diag(1.0E-06,nXX)))
> param <- c('beta','gamma','sigma', 'sd.res', 'sd.mesh','sd.cruise','sd.int','sd.protein','sd.chl','sd.temp')
> csc.jags <- jags(data=csc.list,init=NULL, parameters.to.save=param,
+                  model.file=textConnection(model),
+                  n.chains=3,
+                  n.iter=10000,
+                  n.burnin=1000,
+                  n.thin=10)
```

```
Compiling model graph
   Resolving undeclared variables
   Allocating nodes
   Graph Size: 2394

Initializing model
```

```
> csc.jags
```

```
Inference for Bugs model at "5", fit using jags,
 3 chains, each with 10000 iterations (first 1000 discarded), n.thin = 10
 n.sims = 2700 iterations saved
            mu.vect sd.vect     2.5%      25%      50%      75%    97.5%  Rhat n.eff
beta[1]      25.049  72.736 -109.549  -25.097   24.395   74.175  169.432 1.001  2700
beta[2]      41.000  24.051   -5.369   24.783   41.019   57.030   88.743 1.001  2700
beta[3]      22.382  22.958  -22.515    7.152   22.352   37.874   67.966 1.001  2700
beta[4]      66.763  23.725   20.329   50.527   66.829   82.325  114.001 1.001  2700
beta[5]      56.153  23.709    8.460   40.582   56.134   71.811  102.268 1.002  1100
beta[6]      10.989  23.118  -34.394   -4.898   10.577   26.957   54.869 1.001  2700
beta[7]     -49.043  33.238 -113.765  -71.587  -48.699  -26.996   16.986 1.001  2700
```

```
beta[8]      -28.224  33.369  -92.057  -50.727  -28.788   -5.915   37.989 1.001  2700
beta[9]      -35.291  31.380  -96.039  -57.027  -35.117  -14.708   27.055 1.001  2700
beta[10]     -34.409  31.981  -95.847  -56.496  -34.915  -12.666   30.913 1.000  2700
beta[11]      -2.786  32.269  -66.741  -24.261   -3.274   18.649   59.278 1.001  2700
beta[12]      46.364  32.017  -17.438   25.223   46.608   67.625  110.122 1.001  2700
gamma[1]      -5.303   5.295  -15.343   -8.984   -5.308   -1.722    5.116 1.001  2400
gamma[2]       6.191  15.478  -23.603   -3.959    5.940   16.139   36.828 1.001  2700
gamma[3]       0.905   2.472   -4.128   -0.793    0.993    2.619    5.673 1.001  2700
sd.chl         5.465   4.264    0.236    2.085    4.628    7.686   15.934 1.003  1700
sd.cruise     35.713   9.357   17.300   29.490   35.723   41.919   54.096 1.001  2400
sd.int        31.441   8.079   16.641   25.833   30.911   36.751   47.949 1.001  2700
sd.mesh       24.179  10.589    5.666   16.383   23.719   31.321   46.464 1.002  2700
sd.protein     7.851   5.372    0.375    3.478    7.098   11.510   19.479 1.001  2700
sd.res        45.645   1.305   43.674   44.672   45.468   46.394   48.644 1.001  2700
sd.temp        5.411   3.967    0.220    2.326    4.629    7.781   14.997 1.001  2700
sigma         45.874   3.469   39.634   43.467   45.699   48.087   53.339 1.002  1800
deviance    1080.290   6.186 1070.653 1075.793 1079.588 1083.926 1094.069 1.000  2700

For each parameter, n.eff is a crude measure of effective sample size,
and Rhat is the potential scale reduction factor (at convergence, Rhat=1).

DIC info (using the rule, pD = var(deviance)/2)
pD = 19.1 and DIC = 1099.4
DIC is an estimate of expected predictive error (lower deviance is better).
```

```
> ## Finite-population standard deviations
> X.mesh.var <- csc.jags$BUGSoutput$sims.list[['sd.mesh']]^2
> X.protein.var <- csc.jags$BUGSoutput$sims.list[['sd.protein']]^2
> X.chl.var <- csc.jags$BUGSoutput$sims.list[['sd.chl']]^2
> X.temp.var <- csc.jags$BUGSoutput$sims.list[['sd.temp']]^2
> X.cruise.var <- csc.jags$BUGSoutput$sims.list[['sd.cruise']]^2
> X.int.var <- csc.jags$BUGSoutput$sims.list[['sd.int']]^2
> R.var <- csc.jags$BUGSoutput$sims.list[['sd.res']]^2
>
> R2.X<-(X.mesh.var + X.cruise.var + X.int.var + X.protein.var + X.chl.var + X.temp.var)/(X.mesh.var + X.cruise.var + X.int.var + X.protein.var + X.chl
> R2.mesh<-((X.mesh.var)/(X.mesh.var + X.cruise.var + X.int.var + X.protein.var + R.var))
> R2.cruise<-((X.cruise.var)/(X.mesh.var + X.cruise.var + X.int.var + X.protein.var + X.chl.var + X.temp.var + R.var))
> R2.int<-((X.int.var)/(X.mesh.var + X.cruise.var + X.int.var + X.protein.var + X.chl.var + X.temp.var + R.var))
> R2.protein<-((X.protein.var)/(X.mesh.var + X.cruise.var + X.int.var + X.protein.var + X.chl.var + X.temp.var + R.var))
> R2.chl<-((X.chl.var)/(X.mesh.var + X.cruise.var + X.int.var + X.chl.var + X.chl.var + X.temp.var + R.var))
> R2.temp<-((X.temp.var)/(X.mesh.var + X.cruise.var + X.int.var + X.temp.var + X.chl.var + X.temp.var + R.var))
> R2.resid <- ((R.var)/(X.mesh.var + X.cruise.var + X.int.var + X.protein.var + X.chl.var + X.temp.var + R.var))
>
> a<-cbind(Residual=R.var, 'Temperature'=X.temp.var, 'Chlorophyll'=X.chl.var, 'Protein'=X.protein.var,'Mesh x Cruise'=X.int.var, Cruise=X.cruise.var, M
> sdtab <- cbind(Name=c('Residuals','Temperature','Chlorophyll','Protein','Mesh x Cruise','Cruise','Mesh'),adply(a, 2, function(x) {
+     data.frame(Mean=mean(x), Median=median(x), HPDinterval(as.mcmc(x)), HPDinterval(as.mcmc(x), p=0.68))
+     }))
>
> a<-cbind(Residual=R2.resid, 'Temperature'=R2.temp, 'Chlorophyll'=R2.chl, 'Protein'=R2.protein, 'Mesh x Cruise'=R2.int, Cruise=R2.cruise, Mesh=R2.mesh
> R2tab <- cbind(Name=c('Residuals','Temperature','Chlorophyll','Protein','Mesh x Cruise','Cruise','Mesh'),adply(a, 2, function(x) {
+     data.frame(Mean=mean(x), Median=median(x), HPDinterval(as.mcmc(x)), HPDinterval(as.mcmc(x), p=0.68))
+     }))
> sdtab$R2 <- round(R2tab$Median,3)
>
>
> sdtab$Name <- factor(sdtab$Name, levels=c('Residuals','Temperature','Chlorophyll','Protein','Mesh x Cruise','Cruise','Mesh'))
> sdtab <- subset(sdtab, select=-X1)
>
> print(xtable(sdtab[7:1,], caption='Mean, median, (lower and upper) and highest probability density intervals of finite-population standard deviations
```

% latex table generated in R 3.1.0 by xtable 1.7-4 package % Thu May 7 08:35:41 2015

Table S2. Mean, median, (lower and upper) and highest probability density intervals of finite-population standard deviations as well as marginal R^2 approximations.

Name	Mean	Median	lower	upper	lower.1	upper.1	R2
Mesh	696.72	562.60	0.25	1816.46	1.04	838.47	0.12
Cruise	1362.91	1276.16	211.12	2682.70	541.59	1795.14	0.25
Mesh x Cruise	1053.77	955.49	219.58	2120.44	389.81	1332.99	0.19
Protein	90.49	50.39	0.00	299.07	0.00	100.07	0.01
Chlorophyll	48.05	21.42	0.00	195.23	0.00	44.91	0.00
Temperature	45.01	21.43	0.00	169.90	0.00	45.69	0.00
Residuals	2085.21	2067.31	1891.24	2328.62	1938.82	2154.55	0.40

```
> p1 <- ggplot(sdtab, aes(y=Name,x=Median)) +
+     geom_vline(xintercept=0,linetype='dashed')+
+         geom_errorbarh(aes(xmin=lower, xmax=upper), height=0)+
+                     geom_errorbarh(aes(xmin=lower.1, xmax=upper.1), height=0, size=2)+
+         geom_point(size=3, shape=21, fill='white')+
+             geom_text(aes(label=R2, y=as.numeric(Name)+0.3),position=position_dodge(height=1), size=3)+
+             scale_x_continuous('Finite-population standard deviation')+
+             theme_classic(8)+theme(axis.title.y=element_blank(),axis.title.x=element_text(vjust=-2, size=rel(1.25)),
+                                  plot.margin=unit(c(0,0,2,2), 'lines'))
> print(p1)
```

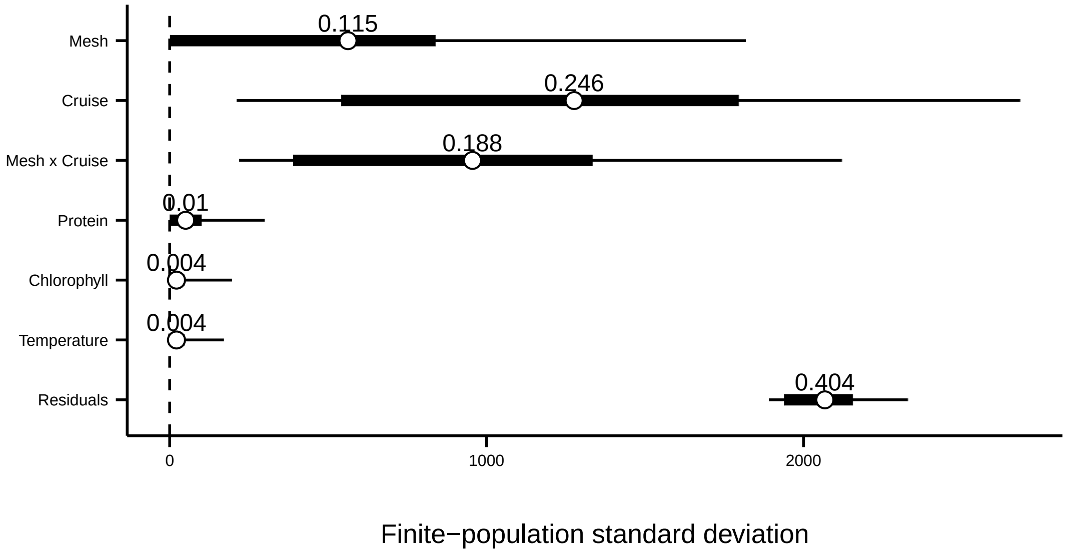

Figure P. Median (lower and upper) highest probability density intervals of finite-population standard deviations. R^2 approximations above medians.

```
> newdata <- expand.grid(Mesh=levels(csc$Mesh), Cruise=levels(csc$Cruise))
> newdata$Protein <- mean(csc$Protein, na.rm=TRUE)
> newdata$Chl <- mean(csc$Chl, na.rm=TRUE)
> newdata$Temp <- mean(csc$Temp, na.rm=TRUE)
> Xmat <- model.matrix(~Mesh*Cruise, data=newdata)
> XXmat <- model.matrix(~log(Protein)+log(Chl)+Temp, data=newdata)[,-1]
> coef <- csc.jags$BUGSoutput$sims.list[[’beta’]]
> fit <- coef %*% t(Xmat) + csc.jags$BUGSoutput$sims.list[[’gamma’]] %*% t(XXmat)
> library(plyr)
> newdata<- cbind(newdata, adply(fit, 2, function(x) {
+     data.frame(Mean=mean(x), Median=median(x), HPDinterval(as.mcmc(x)),HPDinterval(as.mcmc(x), p=0.68))
+     }))
>
> #newdata
>
> #Comparisons
> MCMCsum <- function(x) {
+    data.frame(Median=median(x, na.rm=TRUE),
+               t(quantile(x,na.rm=TRUE)),
+               HPDinterval(as.mcmc(x)),
+               HPDinterval(as.mcmc(x), p=0.68))
+ }
> library(multcomp)
> ndata <- newdata
> ndata<- subset(ndata, Cruise==’1’)
> Xmat <- contrMat(n=rep(1,nrow(ndata)), type="Tukey") %*% model.matrix(~Mesh*Cruise, ndata, xlev=list(Mesh=levels(csc$Mesh), Cruise=levels(csc$Cruise)
> wch <- ldply(strsplit(rownames(Xmat)," - "),as.numeric)
> rownames(Xmat) <- paste(interaction(newdata$Mesh, newdata$Cruise)[wch[,1]],"-",interaction(newdata$Mesh,newdata$Cruise)[wch[,2]])
>  pairwise.comps <- coef %*% t(Xmat)
> comp.mesh_Cruise1<-adply(pairwise.comps,2, MCMCsum)
>
> ndata <- newdata
> ndata<- subset(ndata, Cruise==’2’)
> Xmat <- contrMat(n=rep(1,nrow(ndata)), type="Tukey") %*% model.matrix(~Mesh*Cruise, ndata, xlev=list(Mesh=levels(csc$Mesh), Cruise=levels(csc$Cruise)
> wch <- ldply(strsplit(rownames(Xmat)," - "),as.numeric)
> rownames(Xmat) <- paste(interaction(newdata$Mesh, newdata$Cruise)[wch[,1]],"-",interaction(newdata$Mesh,newdata$Cruise)[wch[,2]])
>  pairwise.comps <- coef %*% t(Xmat)
> comp.mesh_Cruise2<-adply(pairwise.comps,2, MCMCsum)
>
> ndata <- newdata
> ndata<- subset(ndata, Cruise==’3’)
> Xmat <- contrMat(n=rep(1,nrow(ndata)), type="Tukey") %*% model.matrix(~Mesh*Cruise, ndata, xlev=list(Mesh=levels(csc$Mesh), Cruise=levels(csc$Cruise)
> wch <- ldply(strsplit(rownames(Xmat)," - "),as.numeric)
> rownames(Xmat) <- paste(interaction(newdata$Mesh, newdata$Cruise)[wch[,1]],"-",interaction(newdata$Mesh,newdata$Cruise)[wch[,2]])
>  pairwise.comps <- coef %*% t(Xmat)
> comp.mesh_Cruise3<-adply(pairwise.comps,2, MCMCsum)
>
> ndata <- newdata
> ndata<- subset(ndata, Cruise==’4’)
> Xmat <- contrMat(n=rep(1,nrow(ndata)), type="Tukey") %*% model.matrix(~Mesh*Cruise, ndata, xlev=list(Mesh=levels(csc$Mesh), Cruise=levels(csc$Cruise)
> wch <- ldply(strsplit(rownames(Xmat)," - "),as.numeric)
> rownames(Xmat) <- paste(interaction(newdata$Mesh, newdata$Cruise)[wch[,1]],"-",interaction(newdata$Mesh,newdata$Cruise)[wch[,2]])
>  pairwise.comps <- coef %*% t(Xmat)
> comp.mesh_Cruise4<-adply(pairwise.comps,2, MCMCsum)
>
> comp.mesh <- rbind(data.frame(Cruise=1,comp.mesh_Cruise1),
+                    data.frame(Cruise=2,comp.mesh_Cruise2),
+                    data.frame(Cruise=3,comp.mesh_Cruise3),
+                    data.frame(Cruise=4,comp.mesh_Cruise4))
>
> p1 <- ggplot(comp.mesh, aes(x=Cruise, y=Median, color=X1)) +
+        geom_hline(yintercept=0,linetype=’dashed’)+
+                    geom_errorbar(aes(ymin=lower, ymax=upper), width=0, position=position_dodge(width=0.5))+
+                    geom_errorbar(aes(ymin=lower.1, ymax=upper.1), width=0, size=2, position=position_dodge(width=0.5))+
+                        geom_point(size=3, shape=21, fill=’white’,position=position_dodge(width=0.5))+
+                            scale_y_continuous(’Median effect size’)+
+                                #scale_x_discrete(’Cruise number’)+
+                                scale_color_manual(’Mesh contrasts’, breaks=c(’150.1 - 73.1’, ’350.1 - 73.1’,’350.1 - 150.1’), labels=c(’150 vs 73’,’
+                            coord_flip()+theme_classic()+
+                                theme(axis.title.y=element_text(size=rel(1.25), vjust=2),
+                                      axis.title.x=element_text(size=rel(1.25), vjust=-1),
+                                      plot.margin=unit(c(0,0,1,1), ’lines’))
>
>
>
> ndata <- newdata
> ndata<- subset(ndata, Mesh==’73’)
> Xmat <- contrMat(n=rep(1,nrow(ndata)), type="Tukey") %*% model.matrix(~Mesh*Cruise, ndata, xlev=list(Mesh=levels(csc$Mesh), Cruise=levels(csc$Cruise)
>  pairwise.comps <- coef %*% t(Xmat)
> comp.cruise_Mesh73<-adply(pairwise.comps,2, MCMCsum)
>
> ndata <- newdata
> ndata<- subset(ndata,Mesh==’150’)
> Xmat <- contrMat(n=rep(1,nrow(ndata)), type="Tukey") %*% model.matrix(~Mesh*Cruise, ndata, xlev=list(Mesh=levels(csc$Mesh), Cruise=levels(csc$Cruise)
>  pairwise.comps <- coef %*% t(Xmat)
> comp.cruise_Mesh150<-adply(pairwise.comps,2, MCMCsum)
>
> ndata <- newdata
> ndata<- subset(ndata, Mesh==’350’)
> Xmat <- contrMat(n=rep(1,nrow(ndata)), type="Tukey") %*% model.matrix(~Mesh*Cruise, ndata, xlev=list(Mesh=levels(csc$Mesh), Cruise=levels(csc$Cruise)
>  pairwise.comps <- coef %*% t(Xmat)
> comp.cruise_Mesh350<-adply(pairwise.comps,2, MCMCsum)
>
> comp.cruise <- rbind(data.frame(Mesh=73,comp.cruise_Mesh73),
+                      data.frame(Mesh=150,comp.cruise_Mesh150),
+                      data.frame(Mesh=350,comp.cruise_Mesh350))
>
```

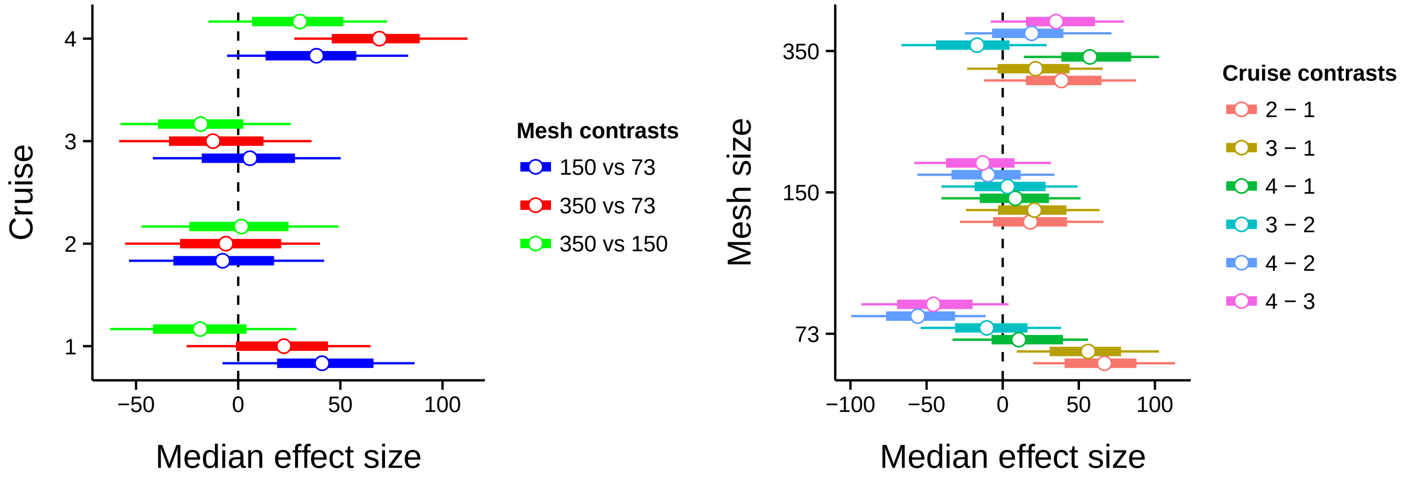

Figure Q. plot of chunk AARScontrasts

3.2. Kimberley

3.2.1. ETS

```
> model="
+ model {
+   for (i in 1:n) {
+     y[i] ~ dnorm(mu[i], tau)
+     mu[i] <- inprod(beta[],X[i,])+inprod(gamma[],XX[i,])
+     y.err[i] <- y[i] - mu[i]
+   }
+   beta ~ dmnorm(a0,A0)
+   gamma ~ dmnorm(c0,C0)
+   tau <- pow(sigma,-2)
+   sigma <- z/sqrt(chSq)
+   z ~ dnorm(0, 0.0016)I(0,)  #1/25^2 = 0.0016
+   chSq ~ dgamma(0.5, 0.5)
+   sd.res <- sd(y.err[])
+   b[1] <- 0
+   b[2:3] <- beta[2:3]
+   sd.mesh <- sd(b)
+   c[1] <-0
+   c[2:4] <- beta[4:6]
+   sd.cruise <- sd(c)
+   for (i in 1:6) {d[i] <-0}
+   d[7:12] <- beta[7:12]
+   sd.int <- sd(d)
+   sd.protein <- abs(gamma[1])*sd(XX[,1])
+   sd.chl <- abs(gamma[2])*sd(XX[,2])
+   sd.temp <- abs(gamma[3])*sd(XX[,3])
+ }
+ "
>
> contrasts(kim$Mesh) <- contr.treatment
> contrasts(kim$Cruise) <- contr.treatment
> dat1 <- subset(kim, !is.na(Protein))
> X <- model.matrix(~Mesh*Cruise, data=dat1)
> nX <- ncol(X)
> XX <- model.matrix(~log(Protein)+log(Chl)+Temp, dat1)[,-1]
> nXX <- ncol(XX)
> kim.list <- with(dat1, list(y=ETS,
+                             n=nrow(dat1),
+                             X=X,nX=nX,
+                             XX=XX,
+                             a0=rep(0,nX), A0=diag(1.0E-06,nX),
+                             c0=rep(0,nXX), C0=diag(1.0E-06,nXX)))
> param <- c('beta','gamma','sigma', 'sd.res', 'sd.mesh','sd.cruise','sd.int','sd.protein','sd.chl','sd.temp')
> kim.jags <- jags(data=kim.list,init=NULL, parameters.to.save=param,
+                  model.file=textConnection(model),
+                  n.chains=3,
+                  n.iter=10000,
+                  n.burnin=1000,
+                  n.thin=10)
```

```
Compiling model graph
   Resolving undeclared variables
   Allocating nodes
   Graph Size: 6558

Initializing model
```

```
> kim.jags
```

```
Inference for Bugs model at "5", fit using jags,
 3 chains, each with 10000 iterations (first 1000 discarded), n.thin = 10
 n.sims = 2700 iterations saved
           mu.vect sd.vect      2.5%      25%      50%      75%    97.5%  Rhat n.eff
beta[1]   -408.238 663.701 -1705.464 -859.748 -422.093   37.516  890.094 1.001  2700
beta[2]     24.288  77.441  -123.030  -29.754   25.680   76.386  177.514 1.001  2700
beta[3]   -272.729  74.573  -419.388 -322.867 -272.271 -223.360 -124.814 1.001  2700
beta[4]    161.658  99.571   -33.598   94.695  161.720  226.206  363.221 1.001  2700
beta[5]    361.399  71.728   219.735  310.879  362.141  411.745  498.871 1.001  2700
```

```
beta[6]     238.714  91.122    65.208  177.459  235.831  300.628  420.637 1.001  2700
beta[7]      36.768  91.606  -145.106  -24.503   35.148   98.409  215.701 1.001  2500
beta[8]    -243.878  99.338  -445.940 -308.708 -244.888 -177.413  -47.409 1.001  2700
beta[9]     -39.248  99.268  -240.870 -104.390  -39.543   26.599  159.832 1.001  2700
beta[10]   -285.742  97.382  -469.397 -352.215 -285.802 -220.899  -93.263 1.001  2700
beta[11]   -308.403  96.098  -485.747 -375.097 -311.573 -243.547 -110.836 1.000  2700
beta[12]   -239.085 110.587  -456.289 -313.517 -241.219 -164.143  -27.326 1.000  2700
beta[13]   -121.640 111.502  -348.982 -194.514 -122.490  -46.978   91.013 1.001  2700
beta[14]   -155.749 103.927  -355.795 -224.795 -156.364  -82.851   44.279 1.001  2200
beta[15]    -25.127 102.848  -231.842  -93.226  -24.245   44.323  172.111 1.001  2700
gamma[1]     21.246  17.153   -11.727    9.339   21.471   32.696   54.100 1.003  1800
gamma[2]     22.561  42.469   -59.310   -6.311   21.976   50.606  108.593 1.001  2700
gamma[3]     34.099  22.356   -10.261   18.968   34.466   49.279   77.478 1.001  2700
sd.chl       23.201  17.302     1.056    9.524   19.796   33.431   65.477 1.001  2700
sd.cruise   161.458  30.193   101.846  141.218  161.270  181.931  221.645 1.001  2700
sd.int      145.934  36.141    80.869  120.319  144.228  169.427  219.618 1.001  2700
sd.mesh     169.497  35.150   101.661  146.409  168.542  193.135  238.492 1.001  2700
sd.protein   25.256  16.174     1.216   12.117   23.807   36.223   59.422 1.001  2100
sd.res      205.882   2.758   201.448  203.878  205.606  207.521  212.032 1.001  2700
sd.temp      54.424  31.391     3.012   30.039   53.109   75.933  119.384 1.005  1900
sigma       206.026   9.408   188.862  199.482  205.422  212.108  226.045 1.002  1200
deviance   3441.492   6.381  3431.047 3437.029 3440.715 3445.050 3456.099 1.001  2700

For each parameter, n.eff is a crude measure of effective sample size,
and Rhat is the potential scale reduction factor (at convergence, Rhat=1).

DIC info (using the rule, pD = var(deviance)/2)
pD = 20.4 and DIC = 3461.9
DIC is an estimate of expected predictive error (lower deviance is better).
```

```
> ## Finite-population standard deviations
> X.mesh.var <- kim.jags$BUGSoutput$sims.list[['sd.mesh']]^2
> X.protein.var <- kim.jags$BUGSoutput$sims.list[['sd.protein']]^2
> X.chl.var <- kim.jags$BUGSoutput$sims.list[['sd.chl']]^2
> X.temp.var <- kim.jags$BUGSoutput$sims.list[['sd.temp']]^2
> X.cruise.var <- kim.jags$BUGSoutput$sims.list[['sd.cruise']]^2
> X.int.var <- kim.jags$BUGSoutput$sims.list[['sd.int']]^2
> R.var <- kim.jags$BUGSoutput$sims.list[['sd.res']]^2
>
> R2.X<-(X.mesh.var + X.cruise.var + X.int.var + X.protein.var + X.chl.var + X.temp.var)/(X.mesh.var + X.cruise.var + X.int.var + X.protein.var + X.chl
> R2.mesh<-((X.mesh.var)/(X.mesh.var + X.cruise.var + X.int.var + X.protein.var + R.var))
> R2.cruise<-((X.cruise.var)/(X.mesh.var + X.cruise.var + X.int.var + X.protein.var + X.chl.var + X.temp.var + R.var))
> R2.int<-((X.int.var)/(X.mesh.var + X.cruise.var + X.int.var + X.protein.var + X.chl.var + X.temp.var + R.var))
> R2.protein<-((X.protein.var)/(X.mesh.var + X.cruise.var + X.int.var + X.protein.var + X.chl.var + X.temp.var + R.var))
> R2.chl<-((X.chl.var)/(X.mesh.var + X.cruise.var + X.int.var + X.chl.var + X.chl.var + X.temp.var + R.var))
> R2.temp<-((X.temp.var)/(X.mesh.var + X.cruise.var + X.int.var + X.temp.var + X.chl.var + X.temp.var + R.var))
> R2.resid <- ((R.var)/(X.mesh.var + X.cruise.var + X.int.var + X.protein.var + X.chl.var + X.temp.var + R.var))
>
> a<-cbind(Residual=R.var, 'Temperature'=X.temp.var, 'Chlorophyll'=X.chl.var, 'Protein'=X.protein.var,'Mesh x Cruise'=X.int.var, Cruise=X.cruise.var, M
> sdtab <- cbind(Name=c('Residuals','Temperature','Chlorophyll','Protein','Mesh x Cruise','Cruise','Mesh'),adply(a, 2, function(x) {
+     data.frame(Mean=mean(x), Median=median(x), HPDinterval(as.mcmc(x)), HPDinterval(as.mcmc(x), p=0.68))
+     }))
>
> a<-cbind(Residual=R2.resid, 'Temperature'=R2.temp, 'Chlorophyll'=R2.chl, 'Protein'=R2.protein, 'Mesh x Cruise'=R2.int, Cruise=R2.cruise, Mesh=R2.mesh
> R2tab <- cbind(Name=c('Residuals','Temperature','Chlorophyll','Protein','Mesh x Cruise','Cruise','Mesh'),adply(a, 2, function(x) {
+     data.frame(Mean=mean(x), Median=median(x), HPDinterval(as.mcmc(x)), HPDinterval(as.mcmc(x), p=0.68))
+     }))
> sdtab$R2 <- round(R2tab$Median,3)
>
>
> sdtab$Name <- factor(sdtab$Name, levels=c('Residuals','Temperature','Chlorophyll','Protein','Mesh x Cruise','Cruise','Mesh'))
> sdtab <- subset(sdtab, select=-X1)
>
> print(xtable(sdtab[7:1,], caption='Mean, median, (lower and upper) and highest probability density intervals of finite-population standard deviations
```

% latex table generated in R 3.1.0 by xtable 1.7-4 package % Thu May 7 08:35:43 2015

Table S3. Mean, median, (lower and upper) and highest probability density intervals of finite-population standard deviations as well as marginal R^2 approximations.

Name	Mean	Median	lower	upper	lower.1	upper.1	R2
Mesh	29964.17	28406.29	7778.75	52971.70	16229.73	39320.80	0.24
Cruise	26980.04	26007.99	9420.74	46329.49	16700.66	35664.58	0.21
Mesh x Cruise	22602.37	20801.81	5278.46	44404.80	9133.18	28755.93	0.17
Protein	899.37	566.79	0.00	2928.71	0.00	1041.05	0.00
Chlorophyll	837.54	391.90	0.00	3254.10	0.00	824.17	0.00
Temperature	3946.92	2820.52	0.00	11661.24	0.00	4722.05	0.02
Residuals	42394.82	42273.88	40408.95	44654.92	40887.71	43050.46	0.34

```
> p1 <- ggplot(sdtab, aes(y=Name,x=Median)) +
+     geom_vline(xintercept=0,linetype='dashed')+
+         geom_errorbarh(aes(xmin=lower, xmax=upper), height=0)+
+                 geom_errorbarh(aes(xmin=lower.1, xmax=upper.1), height=0, size=2)+
+         geom_point(size=3, shape=21, fill='white')+
+             geom_text(aes(label=R2, y=as.numeric(Name)+0.3),position=position_dodge(height=1), size=3)+
+             scale_x_continuous('Finite-population standard deviation')+
+             theme_classic(8)+theme(axis.title.y=element_blank(),axis.title.x=element_text(vjust=-2, size=rel(1.25)),
+                          plot.margin=unit(c(0,0,2,2), 'lines'))
> print(p1)
```

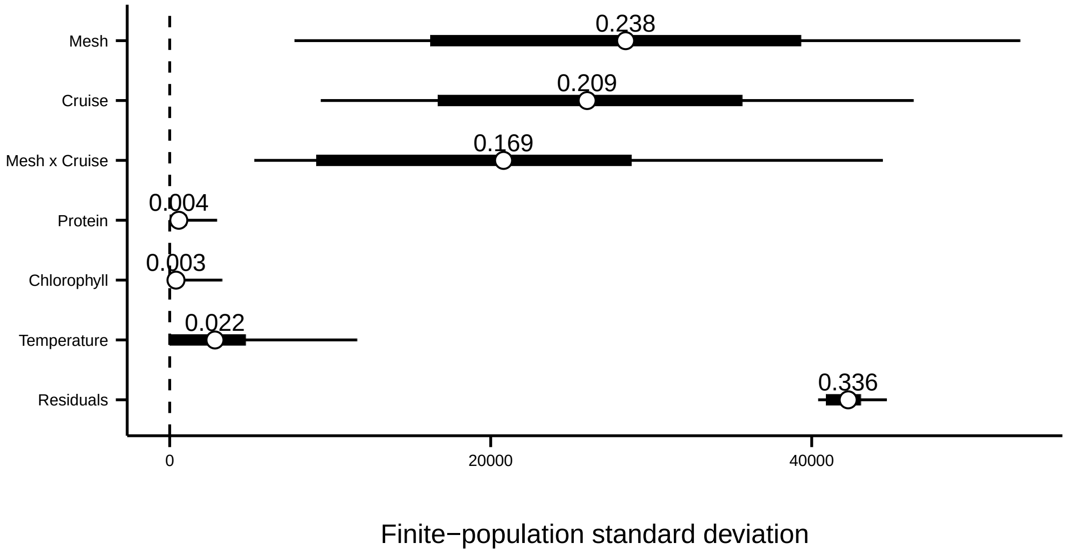

Figure R. Median (lower and upper) highest probability density intervals of finite-population standard deviations. R^2 approximations above medians.

```
> newdata <- expand.grid(Mesh=levels(kim$Mesh), Cruise=levels(kim$Cruise))
> newdata$Protein <- mean(kim$Protein, na.rm=TRUE)
> newdata$Chl <- mean(kim$Chl, na.rm=TRUE)
> newdata$Temp <- mean(kim$Temp, na.rm=TRUE)
> Xmat <- model.matrix(~Mesh*Cruise, data=newdata)
> XXmat <- model.matrix(~log(Protein)+log(Chl)+Temp, data=newdata)[,-1]
> coef <- kim.jags$BUGSoutput$sims.list[['beta']]
> fit <- coef %*% t(Xmat) + kim.jags$BUGSoutput$sims.list[['gamma']] %*% t(XXmat)
> library(plyr)
> newdata<- cbind(newdata, adply(fit, 2, function(x) {
+     data.frame(Mean=mean(x), Median=median(x), HPDinterval(as.mcmc(x)),HPDinterval(as.mcmc(x), p=0.68))
+     }))
>
> #newdata
>
> #Comparisons
> MCMCsum <- function(x) {
+    data.frame(Median=median(x, na.rm=TRUE),
+               t(quantile(x,na.rm=TRUE)),
+               HPDinterval(as.mcmc(x)),
+               HPDinterval(as.mcmc(x), p=0.68))
+ }
> library(multcomp)
> ndata <- newdata
> ndata<- subset(ndata, Cruise=='1')
> Xmat <- contrMat(n=rep(1,nrow(ndata)), type="Tukey") %*% model.matrix(~Mesh*Cruise, ndata, xlev=list(Mesh=levels(kim$Mesh), Cruise=levels(kim$Cruise)
> wch <- ldply(strsplit(rownames(Xmat)," - "),as.numeric)
> rownames(Xmat) <- paste(interaction(newdata$Mesh, newdata$Cruise)[wch[,1]],"-",interaction(newdata$Mesh,newdata$Cruise)[wch[,2]])
>  pairwise.comps <- coef %*% t(Xmat)
> comp.mesh_Cruise1<-adply(pairwise.comps,2, MCMCsum)
>
> ndata <- newdata
> ndata<- subset(ndata, Cruise=='2')
> Xmat <- contrMat(n=rep(1,nrow(ndata)), type="Tukey") %*% model.matrix(~Mesh*Cruise, ndata, xlev=list(Mesh=levels(kim$Mesh), Cruise=levels(kim$Cruise)
> wch <- ldply(strsplit(rownames(Xmat)," - "),as.numeric)
> rownames(Xmat) <- paste(interaction(newdata$Mesh, newdata$Cruise)[wch[,1]],"-",interaction(newdata$Mesh,newdata$Cruise)[wch[,2]])
>  pairwise.comps <- coef %*% t(Xmat)
> comp.mesh_Cruise2<-adply(pairwise.comps,2, MCMCsum)
>
> ndata <- newdata
> ndata<- subset(ndata, Cruise=='3')
> Xmat <- contrMat(n=rep(1,nrow(ndata)), type="Tukey") %*% model.matrix(~Mesh*Cruise, ndata, xlev=list(Mesh=levels(kim$Mesh), Cruise=levels(kim$Cruise)
> wch <- ldply(strsplit(rownames(Xmat)," - "),as.numeric)
> rownames(Xmat) <- paste(interaction(newdata$Mesh, newdata$Cruise)[wch[,1]],"-",interaction(newdata$Mesh,newdata$Cruise)[wch[,2]])
>  pairwise.comps <- coef %*% t(Xmat)
> comp.mesh_Cruise3<-adply(pairwise.comps,2, MCMCsum)
>
> ndata <- newdata
> ndata<- subset(ndata, Cruise=='4')
> Xmat <- contrMat(n=rep(1,nrow(ndata)), type="Tukey") %*% model.matrix(~Mesh*Cruise, ndata, xlev=list(Mesh=levels(kim$Mesh), Cruise=levels(kim$Cruise)
> wch <- ldply(strsplit(rownames(Xmat)," - "),as.numeric)
> rownames(Xmat) <- paste(interaction(newdata$Mesh, newdata$Cruise)[wch[,1]],"-",interaction(newdata$Mesh,newdata$Cruise)[wch[,2]])
>  pairwise.comps <- coef %*% t(Xmat)
> comp.mesh_Cruise4<-adply(pairwise.comps,2, MCMCsum)
>
> ndata <- newdata
> ndata<- subset(ndata, Cruise=='5')
> Xmat <- contrMat(n=rep(1,nrow(ndata)), type="Tukey") %*% model.matrix(~Mesh*Cruise, ndata, xlev=list(Mesh=levels(kim$Mesh), Cruise=levels(kim$Cruise)
> wch <- ldply(strsplit(rownames(Xmat)," - "),as.numeric)
> rownames(Xmat) <- paste(interaction(newdata$Mesh, newdata$Cruise)[wch[,1]],"-",interaction(newdata$Mesh,newdata$Cruise)[wch[,2]])
>  pairwise.comps <- coef %*% t(Xmat)
> comp.mesh_Cruise5<-adply(pairwise.comps,2, MCMCsum)
>
> comp.mesh <- rbind(data.frame(Cruise=1,comp.mesh_Cruise1),
+                    data.frame(Cruise=2,comp.mesh_Cruise2),
+                    data.frame(Cruise=3,comp.mesh_Cruise3),
+                    data.frame(Cruise=4,comp.mesh_Cruise4),
+                    data.frame(Cruise=5,comp.mesh_Cruise5))
>
> p1 <- ggplot(comp.mesh, aes(x=Cruise, y=Median, color=X1)) +
+        geom_hline(yintercept=0,linetype='dashed')+
+                    geom_errorbar(aes(ymin=lower, ymax=upper), width=0, position=position_dodge(width=0.5))+
+                    geom_errorbar(aes(ymin=lower.1, ymax=upper.1), width=0, size=2, position=position_dodge(width=0.5))+
+                        geom_point(size=3, shape=21, fill='white',position=position_dodge(width=0.5))+
+                            scale_y_continuous('Median effect size')+
+                                #scale_x_discrete('Cruise number')+
+                                scale_color_manual('Mesh contrasts', breaks=c('150.1 - 73.1', '350.1 - 73.1','350.1 - 150.1'), labels=c('150 vs 73','
+                            coord_flip()+theme_classic()+
+                                theme(axis.title.y=element_text(size=rel(1.25), vjust=2),
+                                      axis.title.x=element_text(size=rel(1.25), vjust=-1),
+                                      plot.margin=unit(c(0,0,1,1), 'lines'))
>
>
>
> ndata <- newdata
> ndata<- subset(ndata, Mesh=='73')
> Xmat <- contrMat(n=rep(1,nrow(ndata)), type="Tukey") %*% model.matrix(~Mesh*Cruise, ndata, xlev=list(Mesh=levels(kim$Mesh), Cruise=levels(kim$Cruise)
>  pairwise.comps <- coef %*% t(Xmat)
> comp.cruise_Mesh73<-adply(pairwise.comps,2, MCMCsum)
>
> ndata <- newdata
> ndata<- subset(ndata,Mesh=='150')
> Xmat <- contrMat(n=rep(1,nrow(ndata)), type="Tukey") %*% model.matrix(~Mesh*Cruise, ndata, xlev=list(Mesh=levels(kim$Mesh), Cruise=levels(kim$Cruise)
>  pairwise.comps <- coef %*% t(Xmat)
> comp.cruise_Mesh150<-adply(pairwise.comps,2, MCMCsum)
>
> ndata <- newdata
```

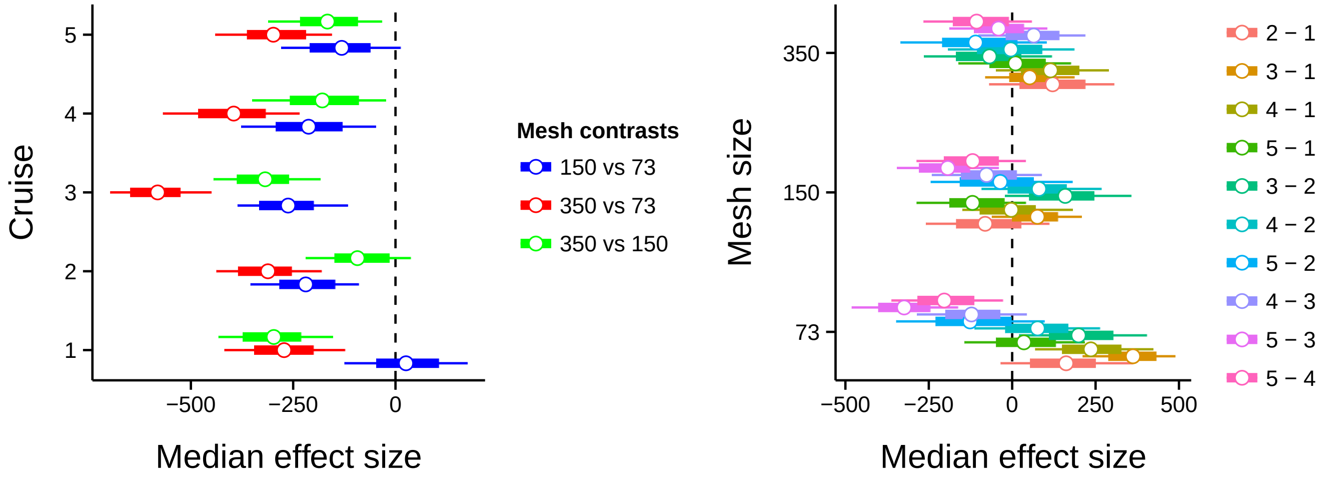

Figure S. plot of chunk ETSKimcontrasts

3.2.2. AARS

```
> model="
+ model {
+   for (i in 1:n) {
+     y[i] ~ dnorm(mu[i], tau)
+     mu[i] <- inprod(beta[],X[i,])+inprod(gamma[],XX[i,])
+     y.err[i] <- y[i] - mu[i]
+   }
+   beta ~ dmnorm(a0,A0)
+   gamma ~ dmnorm(c0,C0)
+   tau <- pow(sigma,-2)
+   sigma <- z/sqrt(chSq)
+   z ~ dnorm(0, 0.0016)I(0,)  #1/25^2 = 0.0016
+   chSq ~ dgamma(0.5, 0.5)
+   sd.res <- sd(y.err[])
+   b[1] <- 0
+   b[2:3] <- beta[2:3]
+   sd.mesh <- sd(b)
+   c[1] <-0
+   c[2:4] <- beta[4:6]
+   sd.cruise <- sd(c)
+   for (i in 1:6) {d[i] <-0}
+   d[7:12] <- beta[7:12]
+   sd.int <- sd(d)
+   sd.protein <- abs(gamma[1])*sd(XX[,1])
+   sd.chl <- abs(gamma[2])*sd(XX[,2])
+   sd.temp <- abs(gamma[3])*sd(XX[,3])
+ }
+ "
>
> contrasts(kim$Mesh) <- contr.treatment
> contrasts(kim$Cruise) <- contr.treatment
> dat1 <- subset(kim, !is.na(Protein))
> X <- model.matrix(~Mesh*Cruise, data=dat1)
> nX <- ncol(X)
> XX <- model.matrix(~log(Protein)+log(Chl)+Temp, dat1)[,-1]
> nXX <- ncol(XX)
> kim.list <- with(dat1, list(y=AARS,
+                             n=nrow(dat1),
+                             X=X,nX=nX,
+                             XX=XX,
+                             a0=rep(0,nX), A0=diag(1.0E-06,nX),
+                             c0=rep(0,nXX), C0=diag(1.0E-06,nXX)))
> param <- c('beta','gamma','sigma', 'sd.res', 'sd.mesh','sd.cruise','sd.int','sd.protein','sd.chl','sd.temp')
> kim.jags <- jags(data=kim.list,init=NULL, parameters.to.save=param,
+                 model.file=textConnection(model),
+                 n.chains=3,
+                 n.iter=10000,
+                 n.burnin=1000,
+                 n.thin=10)
```

```
Compiling model graph
   Resolving undeclared variables
   Allocating nodes
   Graph Size: 6558

Initializing model
```

```
> kim.jags
```

```
Inference for Bugs model at "5", fit using jags,
 3 chains, each with 10000 iterations (first 1000 discarded), n.thin = 10
 n.sims = 2700 iterations saved
            mu.vect sd.vect     2.5%      25%      50%     75%   97.5%  Rhat n.eff
beta[1]    -172.085 222.518 -593.148 -322.049 -174.277 -29.152 271.117 1.001  2700
beta[2]      -8.118  18.203  -42.805  -20.451   -8.132   3.911  27.747 1.001  2700
beta[3]      86.017  17.619   50.192   74.584   86.153  97.796 120.755 1.003   710
beta[4]     109.829  27.775   56.855   91.610  109.791 128.074 164.355 1.002  1300
beta[5]      49.427  17.561   14.601   37.531   49.478  61.352  83.944 1.002  1000
beta[6]      69.788  21.848   25.700   55.362   69.934  84.013 112.446 1.002  1400
beta[7]      44.046  23.176   -2.725   28.892   44.940  59.882  86.460 1.001  2700
```

```
beta[8]      74.024  23.685   28.139   57.789   74.105   89.773  120.428 1.002  1200
beta[9]     -25.033  23.886  -71.769  -41.373  -25.130   -9.010   22.825 1.005   430
beta[10]     40.482  24.296   -8.234   24.490   40.748   57.269   86.565 1.002  1000
beta[11]    -40.524  24.065  -86.929  -56.433  -40.496  -24.553    6.263 1.003   820
beta[12]     44.448  26.808   -9.310   26.548   44.400   61.754   97.419 1.001  2000
beta[13]    -49.394  27.865 -102.928  -68.058  -49.771  -30.796    4.820 1.001  2100
beta[14]    100.601  25.930   49.666   83.252  100.706  118.659  149.568 1.001  2700
beta[15]     10.415  25.375  -38.401   -6.561   10.258   27.308   60.953 1.001  2500
gamma[1]      6.420   4.257   -2.138    3.530    6.395    9.393   14.494 1.001  2700
gamma[2]     21.724  11.184   -0.123   14.446   21.537   29.289   43.647 1.001  2700
gamma[3]      7.430   7.490   -7.468    2.607    7.491   12.515   21.600 1.001  2700
sd.chl       13.238   6.407    1.514    8.718   12.963   17.629   26.271 1.001  2700
sd.cruise    47.575  10.907   27.338   40.163   47.392   54.759   69.859 1.002  1300
sd.int       36.006   6.443   23.638   31.683   35.815   40.350   49.062 1.000  2700
sd.mesh      52.962   8.721   35.757   47.347   53.111   58.630   70.199 1.002  1200
sd.protein    7.327   4.230    0.409    4.032    7.025   10.317   15.919 1.002  2700
sd.res       51.971   0.615   51.000   51.539   51.893   52.340   53.370 1.001  2700
sd.temp      13.481   9.083    0.727    6.195   12.127   19.487   33.454 1.001  2000
sigma        52.069   2.358   47.864   50.446   51.911   53.554   57.051 1.002  1500
deviance   2793.126   6.450 2782.603 2788.464 2792.472 2796.876 2807.700 1.001  2700

For each parameter, n.eff is a crude measure of effective sample size,
and Rhat is the potential scale reduction factor (at convergence, Rhat=1).

DIC info (using the rule, pD = var(deviance)/2)
pD = 20.8 and DIC = 2813.9
DIC is an estimate of expected predictive error (lower deviance is better).
```

```
> ## Finite-population standard deviations
> X.mesh.var <- kim.jags$BUGSoutput$sims.list[['sd.mesh']]^2
> X.protein.var <- kim.jags$BUGSoutput$sims.list[['sd.protein']]^2
> X.chl.var <- kim.jags$BUGSoutput$sims.list[['sd.chl']]^2
> X.temp.var <- kim.jags$BUGSoutput$sims.list[['sd.temp']]^2
> X.cruise.var <- kim.jags$BUGSoutput$sims.list[['sd.cruise']]^2
> X.int.var <- kim.jags$BUGSoutput$sims.list[['sd.int']]^2
> R.var <- kim.jags$BUGSoutput$sims.list[['sd.res']]^2
>
> R2.X<-(X.mesh.var + X.cruise.var + X.int.var + X.protein.var + X.chl.var + X.temp.var)/(X.mesh.var + X.cruise.var + X.int.var + X.protein.var + X.chl
> R2.mesh<-((X.mesh.var)/(X.mesh.var + X.cruise.var + X.int.var + X.protein.var + R.var))
> R2.cruise<-((X.cruise.var)/(X.mesh.var + X.cruise.var + X.int.var + X.protein.var + X.chl.var + X.temp.var + R.var))
> R2.int<-((X.int.var)/(X.mesh.var + X.cruise.var + X.int.var + X.protein.var + X.chl.var + X.temp.var + R.var))
> R2.protein<-((X.protein.var)/(X.mesh.var + X.cruise.var + X.int.var + X.protein.var + X.chl.var + X.temp.var + R.var))
> R2.chl<-((X.chl.var)/(X.mesh.var + X.cruise.var + X.int.var + X.chl.var + X.chl.var + X.temp.var + R.var))
> R2.temp<-((X.temp.var)/(X.mesh.var + X.cruise.var + X.int.var + X.temp.var + X.chl.var + X.temp.var + R.var))
> R2.resid <- ((R.var)/(X.mesh.var + X.cruise.var + X.int.var + X.protein.var + X.chl.var + X.temp.var + R.var))
>
> a<-cbind(Residual=R.var, 'Temperature'=X.temp.var, 'Chlorophyll'=X.chl.var, 'Protein'=X.protein.var,'Mesh x Cruise'=X.int.var, Cruise=X.cruise.var, M
> sdtab <- cbind(Name=c('Residuals','Temperature','Chlorophyll','Protein','Mesh x Cruise','Cruise','Mesh'),adply(a, 2, function(x) {
+     data.frame(Mean=mean(x), Median=median(x), HPDinterval(as.mcmc(x)), HPDinterval(as.mcmc(x), p=0.68))
+     }))
>
> a<-cbind(Residual=R2.resid, 'Temperature'=R2.temp, 'Chlorophyll'=R2.chl, 'Protein'=R2.protein, 'Mesh x Cruise'=R2.int, Cruise=R2.cruise, Mesh=R2.mesh
> R2tab <- cbind(Name=c('Residuals','Temperature','Chlorophyll','Protein','Mesh x Cruise','Cruise','Mesh'),adply(a, 2, function(x) {
+     data.frame(Mean=mean(x), Median=median(x), HPDinterval(as.mcmc(x)), HPDinterval(as.mcmc(x), p=0.68))
+     }))
> sdtab$R2 <- round(R2tab$Median,3)
>
>
> sdtab$Name <- factor(sdtab$Name, levels=c('Residuals','Temperature','Chlorophyll','Protein','Mesh x Cruise','Cruise','Mesh'))
> sdtab <- subset(sdtab, select=-X1)
>
> print(xtable(sdtab[7:1,], caption='Mean, median, (lower and upper) and highest probability density intervals of finite-population standard deviations
```

% latex table generated in R 3.1.0 by xtable 1.7-4 package % Thu May 7 08:35:45 2015

Table S4. Mean, median, (lower and upper) and highest probability density intervals of finite-population standard deviations as well as marginal R^2 approximations.

Name	Mean	Median	lower	upper	lower.1	upper.1	R2
Mesh	2881.02	2820.78	1176.67	4768.52	1758.23	3524.99	0.30
Cruise	2382.32	2245.97	576.33	4552.20	1109.00	3081.09	0.23
Mesh x Cruise	1337.90	1282.73	472.21	2233.68	813.63	1701.69	0.13
Protein	71.57	49.35	0.00	213.00	0.00	85.75	0.01
Chlorophyll	216.27	168.03	0.00	585.12	0.00	264.35	0.02
Temperature	264.21	147.07	0.00	891.61	0.00	294.42	0.02
Residuals	2701.34	2692.84	2584.66	2823.89	2628.70	2746.52	0.28

```
> p1 <- ggplot(sdtab, aes(y=Name,x=Median)) +
+     geom_vline(xintercept=0,linetype='dashed')+
+         geom_errorbarh(aes(xmin=lower, xmax=upper), height=0)+
+                     geom_errorbarh(aes(xmin=lower.1, xmax=upper.1), height=0, size=2)+
+         geom_point(size=3, shape=21, fill='white')+
+             geom_text(aes(label=R2, y=as.numeric(Name)+0.3),position=position_dodge(height=1), size=3)+
+             scale_x_continuous('Finite-population standard deviation')+
+             theme_classic(8)+theme(axis.title.y=element_blank(),axis.title.x=element_text(vjust=-2, size=rel(1.25)),
+                                  plot.margin=unit(c(0,0,2,2), 'lines'))
> print(p1)
```

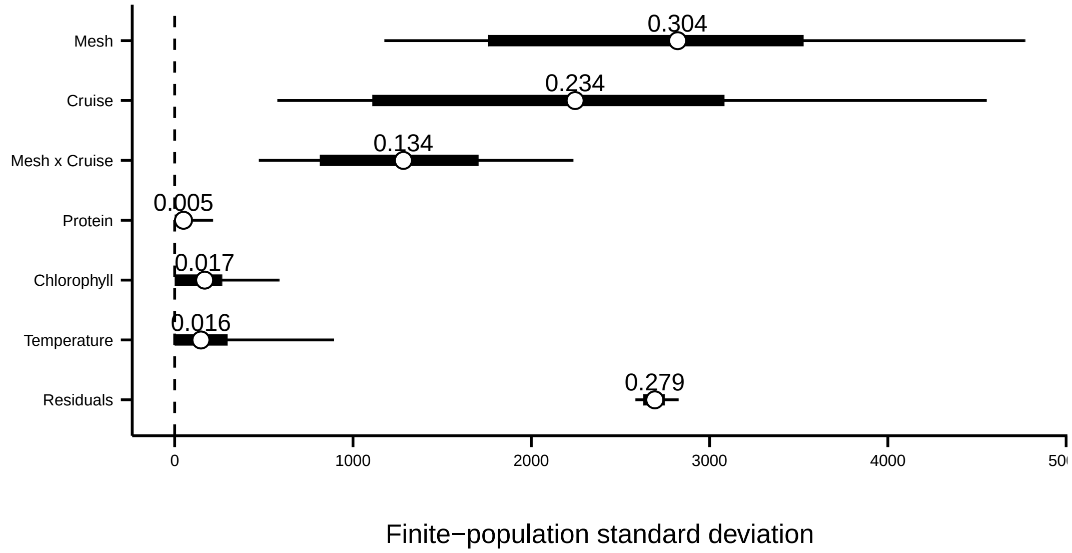

Figure T. Median (lower and upper) highest probability density intervals of finite-population standard deviations. R^2 approximations above medians.

```
> newdata <- expand.grid(Mesh=levels(kim$Mesh), Cruise=levels(kim$Cruise))
> newdata$Protein <- mean(kim$Protein, na.rm=TRUE)
> newdata$Chl <- mean(kim$Chl, na.rm=TRUE)
> newdata$Temp <- mean(kim$Temp, na.rm=TRUE)
> Xmat <- model.matrix(~Mesh*Cruise, data=newdata)
> XXmat <- model.matrix(~log(Protein)+log(Chl)+Temp, data=newdata)[,-1]
> coef <- kim.jags$BUGSoutput$sims.list[['beta']]
> fit <- coef %*% t(Xmat) + kim.jags$BUGSoutput$sims.list[['gamma']] %*% t(XXmat)
> library(plyr)
> newdata<- cbind(newdata, adply(fit, 2, function(x) {
+     data.frame(Mean=mean(x), Median=median(x), HPDinterval(as.mcmc(x)),HPDinterval(as.mcmc(x), p=0.68))
+     }))
>
> #newdata
>
> #Comparisons
> MCMCsum <- function(x) {
+    data.frame(Median=median(x, na.rm=TRUE),
+               t(quantile(x,na.rm=TRUE)),
+               HPDinterval(as.mcmc(x)),
+               HPDinterval(as.mcmc(x), p=0.68))
+ }
> library(multcomp)
> ndata <- newdata
> ndata<- subset(ndata, Cruise=='1')
> Xmat <- contrMat(n=rep(1,nrow(ndata)), type="Tukey") %*% model.matrix(~Mesh*Cruise, ndata, xlev=list(Mesh=levels(kim$Mesh), Cruise=levels(kim$Cruise)
> wch <- ldply(strsplit(rownames(Xmat)," - "),as.numeric)
> rownames(Xmat) <- paste(interaction(newdata$Mesh, newdata$Cruise)[wch[,1]],"-",interaction(newdata$Mesh,newdata$Cruise)[wch[,2]])
>  pairwise.comps <- coef %*% t(Xmat)
> comp.mesh_Cruise1<-adply(pairwise.comps,2, MCMCsum)
>
> ndata <- newdata
> ndata<- subset(ndata, Cruise=='2')
> Xmat <- contrMat(n=rep(1,nrow(ndata)), type="Tukey") %*% model.matrix(~Mesh*Cruise, ndata, xlev=list(Mesh=levels(kim$Mesh), Cruise=levels(kim$Cruise)
> wch <- ldply(strsplit(rownames(Xmat)," - "),as.numeric)
> rownames(Xmat) <- paste(interaction(newdata$Mesh, newdata$Cruise)[wch[,1]],"-",interaction(newdata$Mesh,newdata$Cruise)[wch[,2]])
>  pairwise.comps <- coef %*% t(Xmat)
> comp.mesh_Cruise2<-adply(pairwise.comps,2, MCMCsum)
>
> ndata <- newdata
> ndata<- subset(ndata, Cruise=='3')
> Xmat <- contrMat(n=rep(1,nrow(ndata)), type="Tukey") %*% model.matrix(~Mesh*Cruise, ndata, xlev=list(Mesh=levels(kim$Mesh), Cruise=levels(kim$Cruise)
> wch <- ldply(strsplit(rownames(Xmat)," - "),as.numeric)
> rownames(Xmat) <- paste(interaction(newdata$Mesh, newdata$Cruise)[wch[,1]],"-",interaction(newdata$Mesh,newdata$Cruise)[wch[,2]])
>  pairwise.comps <- coef %*% t(Xmat)
> comp.mesh_Cruise3<-adply(pairwise.comps,2, MCMCsum)
>
> ndata <- newdata
> ndata<- subset(ndata, Cruise=='4')
> Xmat <- contrMat(n=rep(1,nrow(ndata)), type="Tukey") %*% model.matrix(~Mesh*Cruise, ndata, xlev=list(Mesh=levels(kim$Mesh), Cruise=levels(kim$Cruise)
> wch <- ldply(strsplit(rownames(Xmat)," - "),as.numeric)
> rownames(Xmat) <- paste(interaction(newdata$Mesh, newdata$Cruise)[wch[,1]],"-",interaction(newdata$Mesh,newdata$Cruise)[wch[,2]])
>  pairwise.comps <- coef %*% t(Xmat)
> comp.mesh_Cruise4<-adply(pairwise.comps,2, MCMCsum)
>
> ndata <- newdata
> ndata<- subset(ndata, Cruise=='5')
> Xmat <- contrMat(n=rep(1,nrow(ndata)), type="Tukey") %*% model.matrix(~Mesh*Cruise, ndata, xlev=list(Mesh=levels(kim$Mesh), Cruise=levels(kim$Cruise)
> wch <- ldply(strsplit(rownames(Xmat)," - "),as.numeric)
> rownames(Xmat) <- paste(interaction(newdata$Mesh, newdata$Cruise)[wch[,1]],"-",interaction(newdata$Mesh,newdata$Cruise)[wch[,2]])
>  pairwise.comps <- coef %*% t(Xmat)
> comp.mesh_Cruise5<-adply(pairwise.comps,2, MCMCsum)
>
>
> comp.mesh <- rbind(data.frame(Cruise=1,comp.mesh_Cruise1),
+                    data.frame(Cruise=2,comp.mesh_Cruise2),
+                    data.frame(Cruise=3,comp.mesh_Cruise3),
+                    data.frame(Cruise=4,comp.mesh_Cruise4),
+                    data.frame(Cruise=5,comp.mesh_Cruise5))
>
> p1 <- ggplot(comp.mesh, aes(x=Cruise, y=Median, color=X1)) +
+         geom_hline(yintercept=0,linetype='dashed')+
+                     geom_errorbar(aes(ymin=lower, ymax=upper), width=0, position=position_dodge(width=0.5))+
+                     geom_errorbar(aes(ymin=lower.1, ymax=upper.1), width=0, size=2, position=position_dodge(width=0.5))+
+                         geom_point(size=3, shape=21, fill='white',position=position_dodge(width=0.5))+
+                             scale_y_continuous('Median effect size')+
+                                 #scale_x_discrete('Cruise number')+
+                                 scale_color_manual('Mesh contrasts', breaks=c('150.1 - 73.1', '350.1 - 73.1','350.1 - 150.1'), labels=c('150 vs 73','
+                             coord_flip()+theme_classic()+
+                                 theme(axis.title.y=element_text(size=rel(1.25), vjust=2),
+                                       axis.title.x=element_text(size=rel(1.25), vjust=-1),
+                                       plot.margin=unit(c(0,0,1,1), 'lines'))
>
>
>
> ndata <- newdata
> ndata<- subset(ndata, Mesh=='73')
> Xmat <- contrMat(n=rep(1,nrow(ndata)), type="Tukey") %*% model.matrix(~Mesh*Cruise, ndata, xlev=list(Mesh=levels(kim$Mesh), Cruise=levels(kim$Cruise)
>  pairwise.comps <- coef %*% t(Xmat)
> comp.cruise_Mesh73<-adply(pairwise.comps,2, MCMCsum)
>
> ndata <- newdata
> ndata<- subset(ndata,Mesh=='150')
> Xmat <- contrMat(n=rep(1,nrow(ndata)), type="Tukey") %*% model.matrix(~Mesh*Cruise, ndata, xlev=list(Mesh=levels(kim$Mesh), Cruise=levels(kim$Cruise)
>  pairwise.comps <- coef %*% t(Xmat)
> comp.cruise_Mesh150<-adply(pairwise.comps,2, MCMCsum)
```

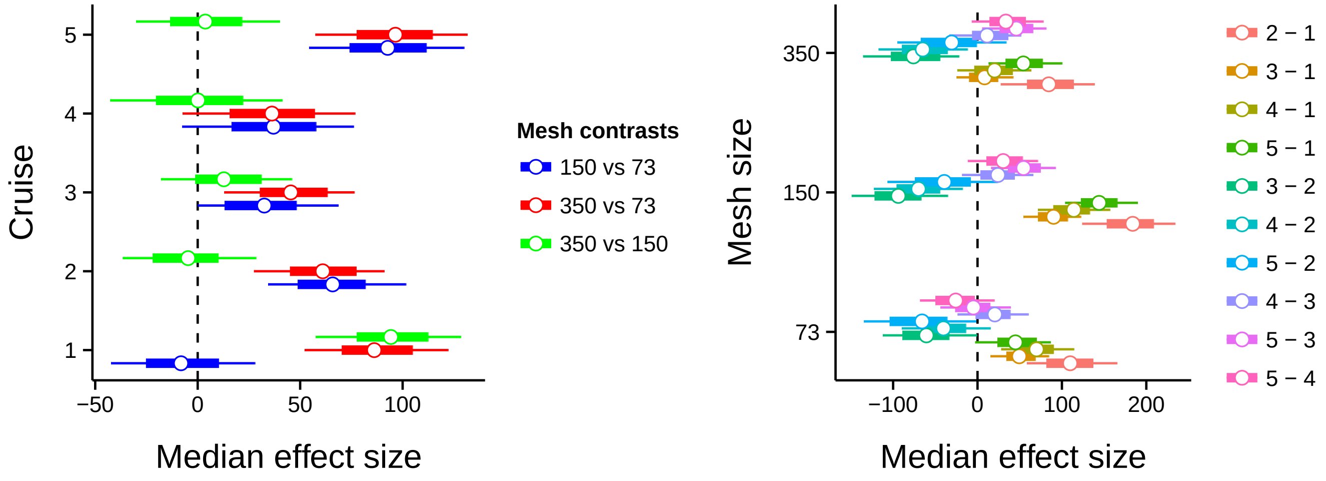

Figure U. plot of chunk AARSKimcontrasts

4. References

This document was produced from markdown using knitr on R version 3.1.0 (2014-04-10) on a x86_64-pc-linux-gnu system.

```
> sessionInfo()
```

```
R version 3.1.0 (2014-04-10)
Platform: x86_64-pc-linux-gnu (64-bit)

locale:
 [1] LC_CTYPE=en_AU.UTF-8       LC_NUMERIC=C               LC_TIME=en_AU.UTF-8
 [4] LC_COLLATE=en_AU.UTF-8     LC_MONETARY=en_AU.UTF-8    LC_MESSAGES=en_AU.UTF-8
 [7] LC_PAPER=en_AU.UTF-8       LC_NAME=C                  LC_ADDRESS=C
[10] LC_TELEPHONE=C             LC_MEASUREMENT=en_AU.UTF-8 LC_IDENTIFICATION=C

attached base packages:
[1] splines   grid      stats     graphics  grDevices utils     datasets  methods   base

other attached packages:
 [1] multcomp_1.3-7  TH.data_1.0-3   survival_2.37-7 mvtnorm_1.0-0   mgcv_1.8-6      nlme_3.1-118
 [7] GGally_0.4.8    xtable_1.7-4    plyr_1.8.1      R2jags_0.04-03  rjags_3-13      coda_0.16-1
[13] lattice_0.20-29 gridExtra_0.9.1 ggplot2_1.0.0   knitr_1.9

loaded via a namespace (and not attached):
 [1] abind_1.4-3         boot_1.3-13         colorspace_1.2-4    digest_0.6.4
 [5] evaluate_0.5.5      formatR_1.0         gtable_0.1.2        labeling_0.3
 [9] MASS_7.3-35         Matrix_1.1-4        munsell_0.4.2       parallel_3.1.0
[13] proto_0.3-10        R2WinBUGS_2.1-19    Rcpp_0.11.3         reshape_0.8.5
[17] reshape2_1.4.1.9000 sandwich_2.3-2      scales_0.2.4        stringr_0.6.2
[21] tools_3.1.0         zoo_1.7-11
```

Gelman A (2006) Prior distributions for variance parameters in hierarchical models. Bayesian Analysis:515–533

Gelman A, Hill J (2007) Data Analysis Using Regression and Multilevel/hierarchical Models. Cambridge University Press, Cambridge, UK

Plummer M (2003) JAGS A program for analysis of Bayesian graphical models using Gibbs sampling. In: Proceedings of the 3rd International Workshop on Distributed Statistical Computing.

R Core Team (2014) R A Language and Environment for Statistical Computing.

Su Y-S, Yajima M (2014) R2jags A Package for Running jags from R.

Team RC (2014) R A Language and Environment for Statistical Computing.
